# Supplementary material for: A metabolic map of the DNA damage response identifies PRDX1 in the control of nuclear ROS scavenging and aspartate availability
Source: Mol Syst Biol. 2023 Jun 1;19(7):e11267. doi: 10.15252/msb.202211267 (PMC10333845; doi:10.15252/msb.202211267)
Supplement: Supplementary file 1 — Appendix [file MSB-19-e11267-s004.pdf]

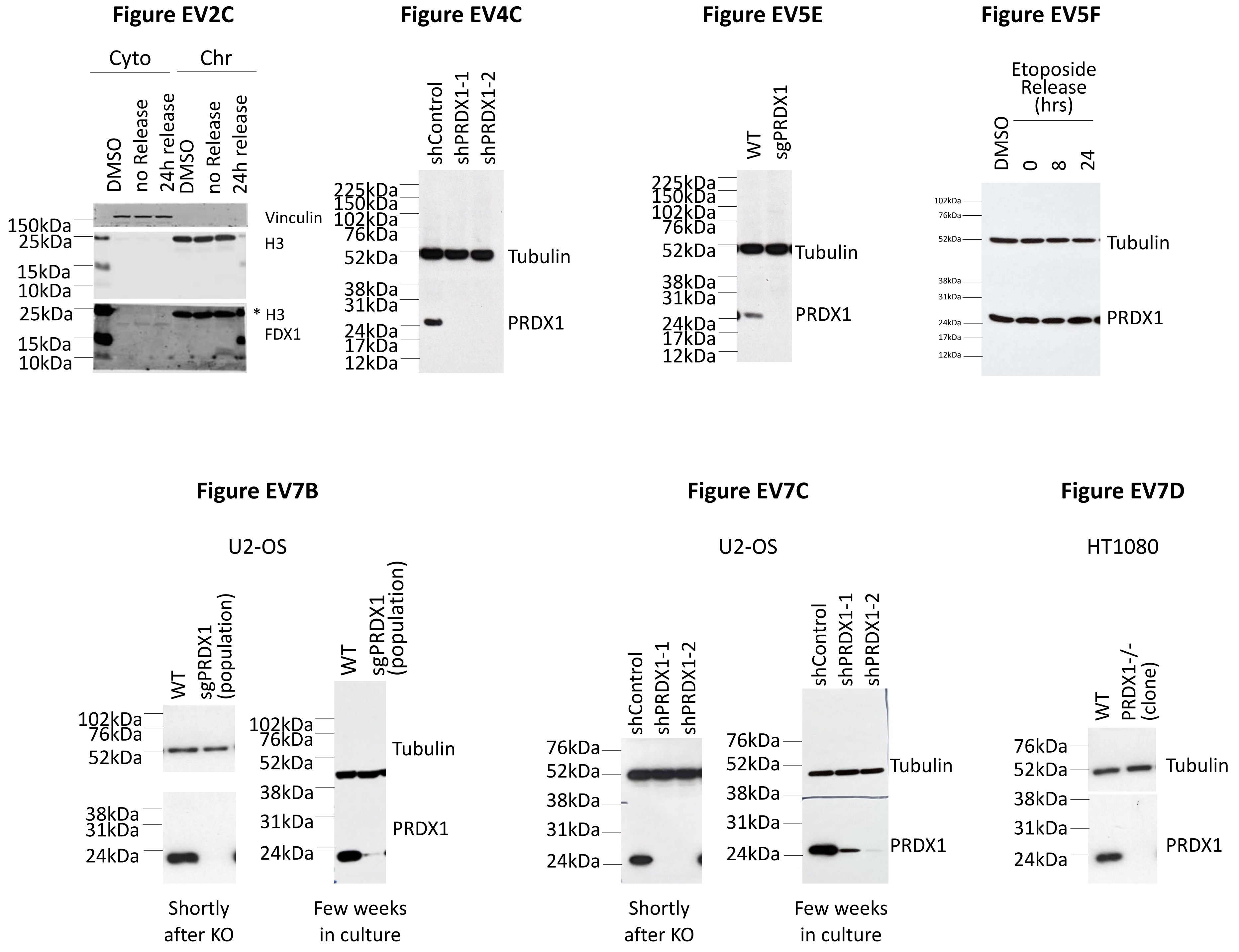

Appendix Figure S1: full size of the western blots presented in the manuscript. Cyto is cytoplasm, Chr is Chromatin
