# Supplementary material for: A metabolic map of the DNA damage response identifies PRDX1 in the control of nuclear ROS scavenging and aspartate availability
Source: Mol Syst Biol. 2023 Jun 1;19(7):e11267. doi: 10.15252/msb.202211267 (PMC10333845; doi:10.15252/msb.202211267)
Supplement: Supplementary file 2 — Expanded View Figures PDF [file MSB-19-e11267-s003.pdf]

Expanded View Figures

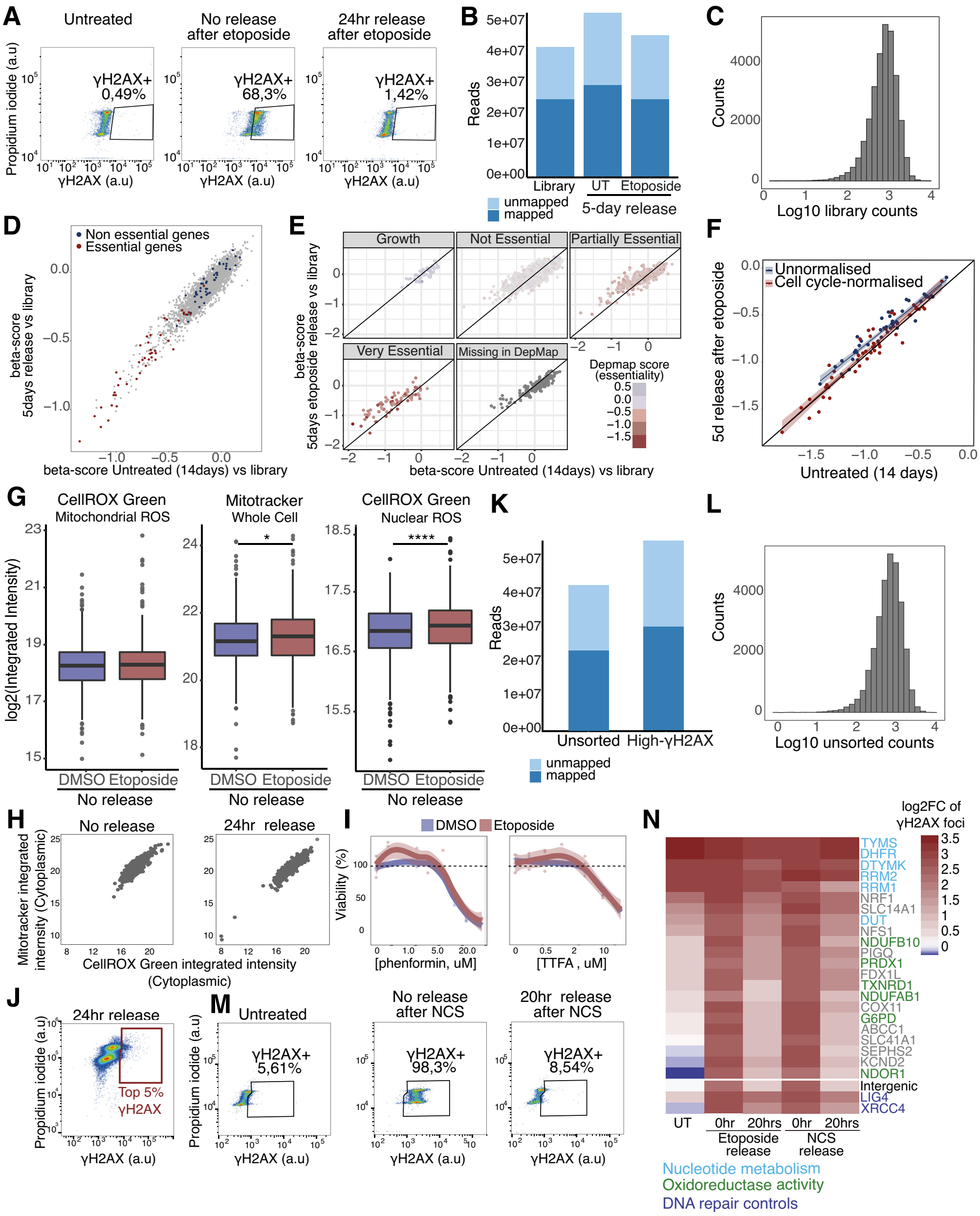

Figure EV1.

 © 2023 The Authors

Figure EV1. Etoposide-release CRISPR-Cas9 screens with a metabolic library.

A FACS monitoring of γH2AX levels following etoposide treatment and after 24 h of release in drug-free media.
B Mapped reads in the etoposide survival CRISPR screen.
C Distribution of reads in the etoposide survival CRISPR screen.
D, E Comparison of β scores separated by gene essentiality according to MaGECKFlute (D) or DepMap (E).
F MaGECKflute cell cycle normalization based on essential genes. Shaded area represents the 95% confidence interval around the median (central line).
G Quantification of CellROX Green and Mitotracker stained U2-OS WT cells in DMSO- and etoposide-treated conditions, represented as nuclear or cytoplasmic integrated intensities of CellROX Green signal and whole cell integrated intensities of Mitotracker. Three biological replicates were performed. A minimum of 1,000 cells were quantified for each condition, using Harmony. Boxplots represent the median within the IQR. *P*-values were calculated using linear regression on the log2 normalized values where ns: not significant ($P > 0.05$), *$P < 0.05$, **$P < 0.01$, ***$P < 0.001$, ****$P < 0.0001$.
H Correlation of cytoplasmic CellROX Green, representing mitochondrial ROS, and Mitotracker integrated intensities of no release and 24 h release after etoposide in U2-OS cells. Three biological replicates were performed. A minimum of 1,000 cells (from three biological-replicate wells) were quantified for each condition, using Harmony.
I Viability of U2-OS cells treated with etoposide and increasing concentrations of various ETC inhibitors. Three biological replicates were performed. Shaded area represents the 95% confidence interval around the median (central line).
J FACS gating strategy for the high-γH2AX CRISPR screen.
K Mapped reads in the high-γH2AX CRISPR screen.
L Distribution of reads in the high-γH2AX CRISPR screen.
M FACS monitoring of γH2AX levels following NCS treatment and after 20 h of release in compound-free media.
N Quantification of the validation arrayed CRISPR screen using CellProfiler and represented as the mean of the log2 fold change compared to the untreated intergenic control of three independent biological replicates.

© 2023 The Authors

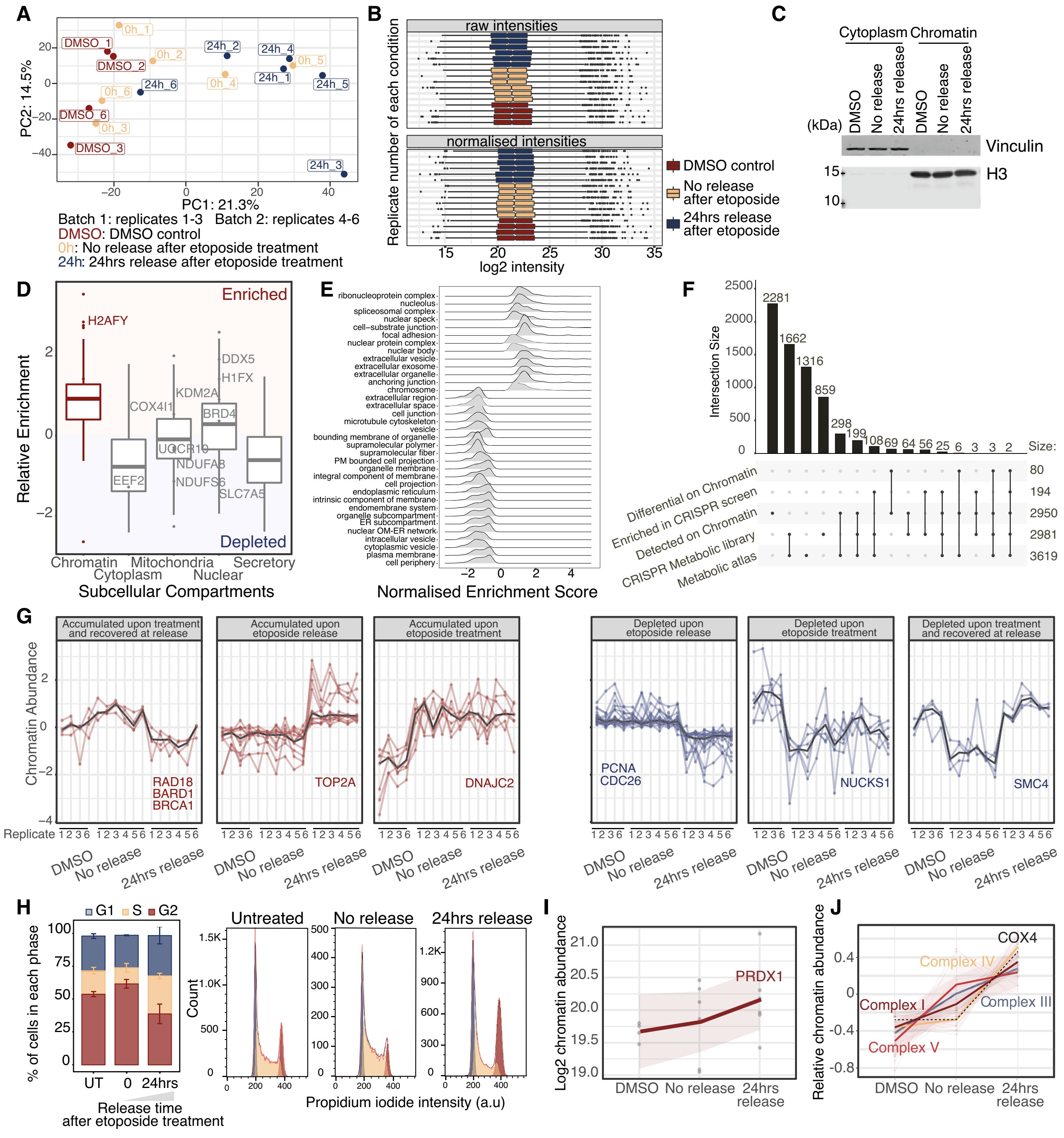

Figure EV2.

 © 2023 The Authors

◀ **Figure EV2. Etoposide-release chromatome proteomics.**

A PCA of batch-corrected chromatin proteomics samples.
B Median normalization of raw proteomics intensities. Boxplots represent IQR around the median value for the samples shown in (A).
C Western blot confirmation of cell fractionation and chromatin enrichment for one representative replicate.
D Enrichment of known chromatin proteins normalized to publicly available U2-OS whole cell extract, based on the mean of at least four biological replicates. Boxplots represent IQR around the median.
E GSEA-Cellular Components of relative enrichment against publicly available U2-OS whole cell extract.
F Overlap of detected and significant genes between the chromatin and CRISPR-Cas9 screen datasets.
G Behavioral clustering of significant proteins with distinct chromatin recruitment patterns. Shaded area represents the 95% confidence interval around the median (central line).
H FACS monitoring cell cycle profiles after etoposide treatment and release of U2-OS WT cells.
I Kinetics of PRDX1 chromatin recruitment upon etoposide release. Shaded area represents the 95% confidence interval around the median (central line) for the biological samples shown in (A).
J Relative kinetics of members of ETC complexes chromatin recruitment upon etoposide release. Each protein is centered to its mean value. Shaded area represents the 95% confidence interval around the median (central line) for the biological samples shown in (A).

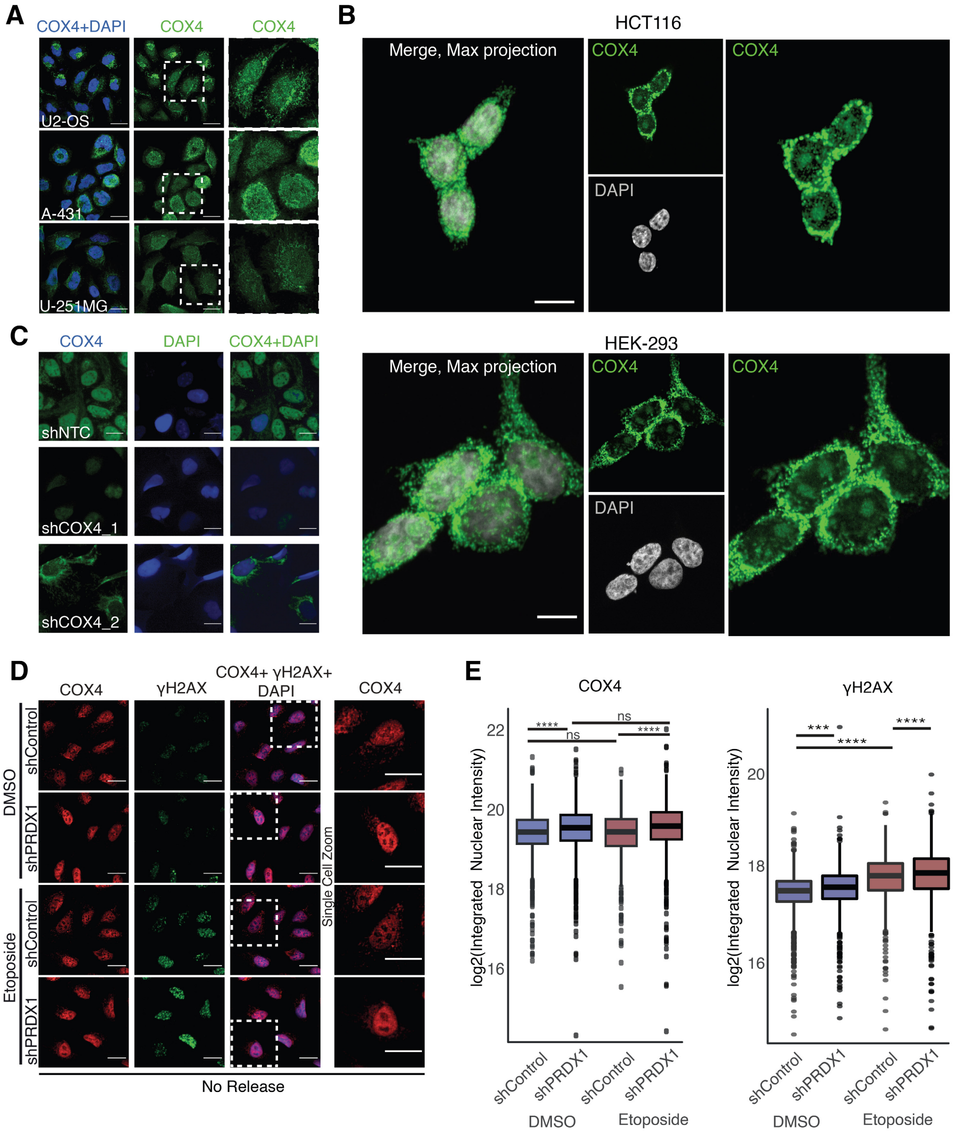

Figure EV3.

◀ **Figure EV3. COX4 nuclear presence.**

A Visualization of COX4 (in green) and within DAPI stained nuclei (in blue) in multiple cell lines. Data obtained from the Human Protein Atlas, available from v22.0.proteinatlas.org. Scale bar is 25 μm.

B Visualization of COX4 (in green) within DAPI stained nuclei (in gray) in HCT116 and HEK293 cells. Images were acquired with a Nikon A1R Ultra-Fast Spectral Scanning Confocal Microscope using a 60× objective. Scale bar is 25 μm.

C Visualization of COX4 (in green) within DAPI stained nuclei (in blue) in U2-OS shControl and shCOX4 cells. Images were acquired with the Operetta High Content Screening System in confocal mode, scale bar is 25 μm.

D Visualization of COX4 (in red) and γH2AX (in green) within DAPI stained nuclei (in blue) in U2-OS shControl and shPRDX1 cells at the indicated treatment conditions. Images were acquired with the Operetta High Content Screening System in confocal mode, scale bar is 25 μm.

E Quantification of images shown in (D), represented as nuclear integrated intensities of γH2AX and COX4 signals. Three biological replicates were perfomed. A minimum of 1,000 cells were quantified for each condition, using Harmony. Boxplots represent the median within the IQR. *P*-values were calculated using linear regression on the log2 normalized values and the interaction term *P*-value between PRDX1 background and etoposide treatment is shown in the plot, where ns: not significant ($P > 0.05$), $*P < 0.05$, $**P < 0.01$, $***P < 0.001$, $****P < 0.0001$.

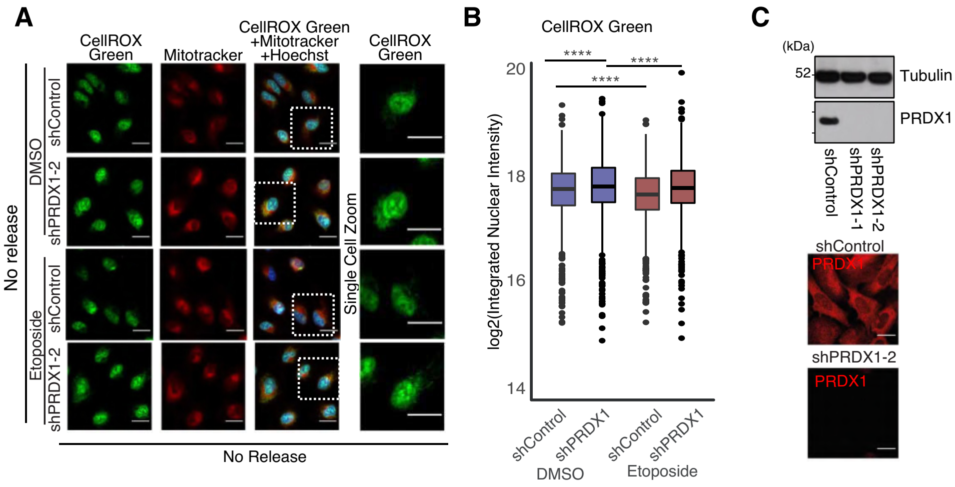

Figure EV4. Nuclear ROS accumulates in the absence of PRDX1.

A Visualization of ROS (CellROX Green, in green) and mitochondria (Mitotracker, in red) within Hoechst-stained nuclei (in blue) in U2-OS shControl and shPRDX1 cells at the indicated treatment conditions. Images were acquired with the Operetta High Content Screening System in confocal mode, scale bar is 25 μm.

B Quantification of images shown in (A), represented as log2 nuclear-integrated intensity of CellROX Green and Mitotracker without Etoposide release compared to DMSO control. Three biological replicates were perfomed. A minimum of 1,000 cells were quantified for each condition, using Harmony. *P*-values were calculated using linear regression on the log2 normalized values and the interaction term *P*-value between PRDX1 background and etoposide treatment is shown in the plot, where ns: not significant ($P > 0.05$), $*P < 0.05$, $**P < 0.01$, $***P < 0.001$, $****P < 0.0001$.

C Validation of shPRDX1 through Immunoblot (top) for PRDX1 and Tubulin on protein extracts from U2-OS shControl and shPRDX1 cell populations and visualization of PRDX1 (in red) in U2-OS shControl and shCOX4 cells (bottom). Images were acquired with the Operetta High Content Screening System in confocal mode, scale bar is 25 μm.

 © 2023 The Authors

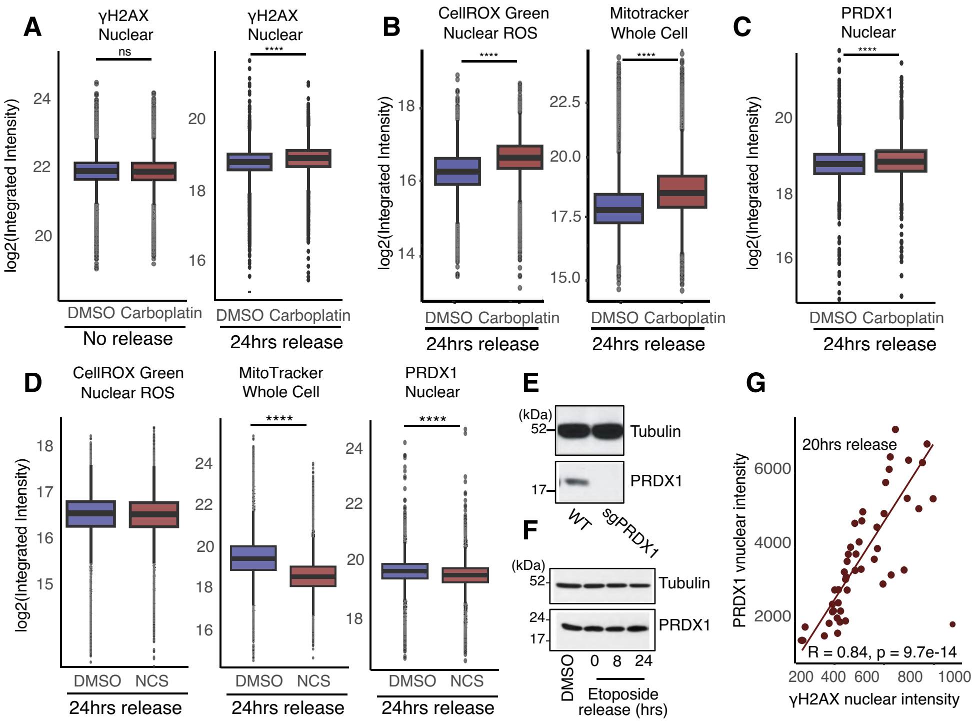

Figure EV5. PRDX1 nuclear abundance is related to DNA damage levels.

A Quantification of nuclear γH2AX integrated intensity without release or 24 h release from Carboplatin compared to DMSO control, in U2-OS WT cells. Three biological replicates were performed. A minimum of 1,000 cells were quantified for each condition, using Harmony. Boxplots represent the median within the IQR. *P*-values were calculated using the Student's *t*-test where ns: not significant ($P > 0.05$), *$P < 0.05$, **$P < 0.01$, ***$P < 0.001$, ****$P < 0.0001$.

B Quantification of nuclear ROS and whole-cell Mitotracker integrated intensity at 24 h release from Carboplatin compared to DMSO control, in U2-OS WT cells. Three biological replicates were performed. A minimum of 1,000 cells were quantified for each condition, using Harmony. Boxplots represent the median within the IQR. *P*-values were calculated using the Student's *t*-test where ns: not significant ($P > 0.05$), *$P < 0.05$, **$P < 0.01$, ***$P < 0.001$, ****$P < 0.0001$.

C Quantification of nuclear PRDX1 integrated intensity at 24 h of release from Carboplatin compared to DMSO control, in U2-OS WT cells. Three biological replicates were performed. A minimum of 1,000 cells were quantified for each condition, using Harmony. Boxplots represent the median within the IQR. *P*-values were calculated using the Student's *t*-test where ns: not significant ($P > 0.05$), *$P < 0.05$, **$P < 0.01$, ***$P < 0.001$, ****$P < 0.0001$.

D Quantification of nuclear ROS, whole-cell Mitotracker, and nuclear PRDX1 integrated intensity at 24 h of release from NCS compared to DMSO control, in U2-OS WT cells. Three biological replicates were performed. A minimum of 1,000 cells were quantified for each condition, using Harmony. Boxplots represent the median within the IQR. *P*-values were calculated using the Student's *t*-test where ns: not significant ($P > 0.05$), *$P < 0.05$, **$P < 0.01$, ***$P < 0.001$, ****$P < 0.0001$.

E Immunoblot for PRDX1 and Tubulin on protein extracts from U2-OS WT and sgPRDX1 cells.

F Immunoblot showing PRDX1 abundance in total extracts of U2-OS cells after etoposide treatment and release in drug-free media. Tubulin is used as a loading control.

G Correlation between γH2AX and PRDX1 nuclear-integrated intensities in U2-OS WT cells at 20 h etoposide release.

© 2023 The Authors

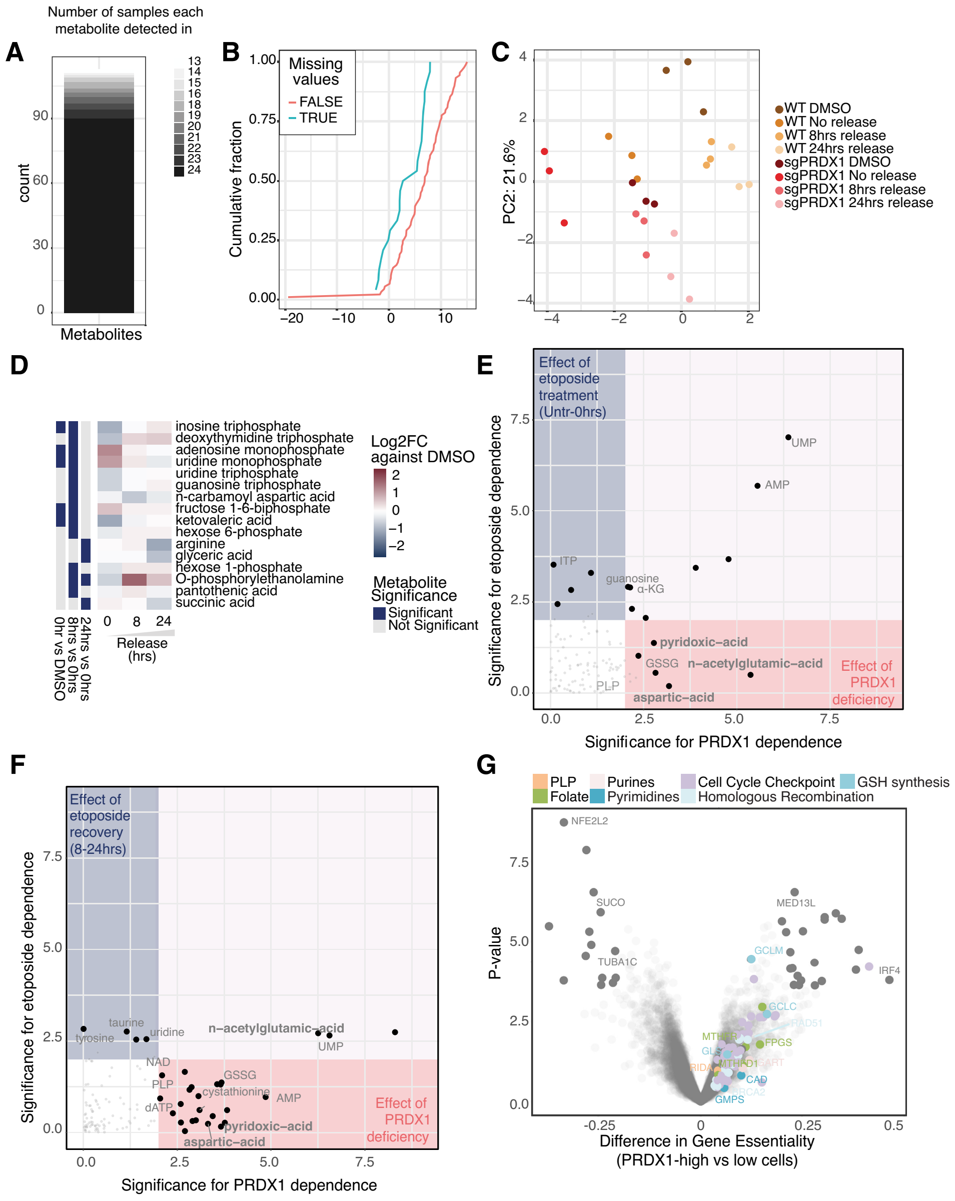

Figure EV6. Etoposide-release metabolomics.

- A Number of metabolites detected per sample in the targeted metabolomics experiment.
- B Distribution of intensities of consistently detected- and partially detected metabolites showing an intensity-dependent detection pattern.
- C PCA plot for all samples in the metabolomics experiment.
- D Significantly affected metabolites due to etoposide treatment and 24-h release in U2- OS sgPRDX1 cells.
- E, F PRDX1 deficiency-dependency and etoposide treatment-dependency of analyzed metabolites, based on linear regression analysis on the Untreated – 0 h (E) and 8–24 h release timepoints (F).
- G Differential gene essentiality between high and low PRDX1-expressing cell lines (CCLE) as in the Achilles dataset. Cell lines with low PRDX1 expression are significantly more sensitive to the depletion of genes represented in the right part of the x-axis as compared to cell lines with high PRDX1 expression.

 © 2023 The Authors

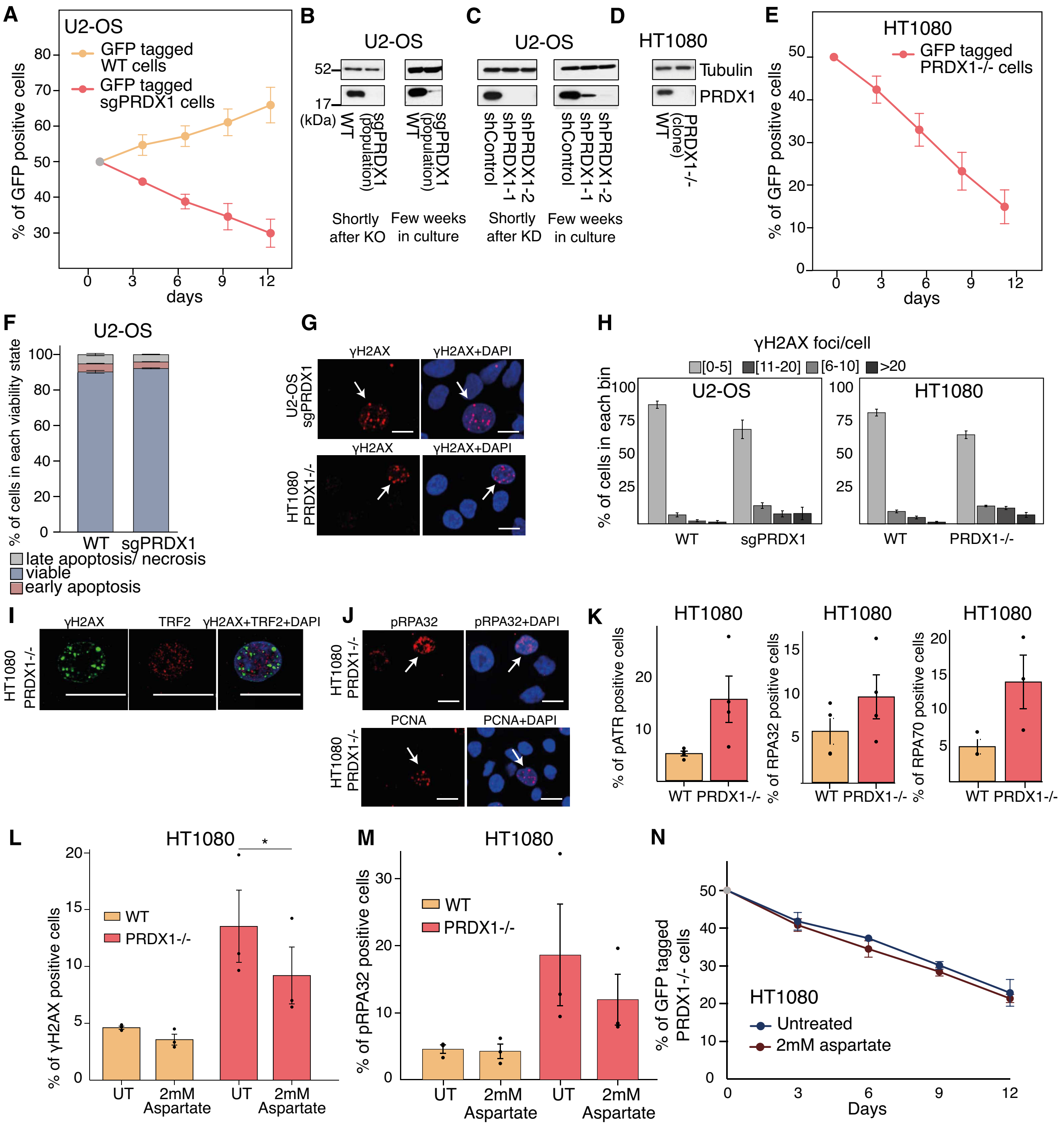
A
U2-OS
GFP tagged WT cells
GFP tagged sgPRDX1 cells
% of GFP positive cells
days
B
U2-OS
WT
sgPRDX1 (population)
Shortly after KO
Few weeks in culture
C
U2-OS
shControl
shPRDX1-1
shPRDX1-2
Shortly after KD
Few weeks in culture
D
HT1080
Tubulin
PRDX1
WT
PRDX1-/- (clone)
E
HT1080
GFP tagged PRDX1-/- cells
% of GFP positive cells
days
F
U2-OS
% of cells in each viability state
WT
sgPRDX1
late apoptosis/ necrosis
viable
early apoptosis
G
γH2AX
γH2AX+DAPI
U2-OS sgPRDX1
HT1080 PRDX1-/-
H
γH2AX foci/cell
[0-5] [11-20] [6-10] >20
% of cells in each bin
U2-OS
HT1080
WT
sgPRDX1
PRDX1-/-
I
γH2AX
TRF2
γH2AX+TRF2+DAPI
HT1080 PRDX1-/-
J
pRPA32
pRPA32+DAPI
PCNA
PCNA+DAPI
HT1080 PRDX1-/-
K
HT1080
% of pATR positive cells
% of RPA32 positive cells
% of RPA70 positive cells
WT PRDX1-/-
L
HT1080
% of γH2AX positive cells
WT
PRDX1-/-
UT
2mM Aspartate
M
HT1080
% of pRPA32 positive cells
N
HT1080
Untreated
2mM aspartate
% of GFP tagged PRDX1-/- cells
Days

Figure EV7.

Figure EV7. PRDX1 loss generates DNA damage that can be partially rescued by exogenous aspartate supplementation.

A Competitive growth assay of U2-OS WT and sgPRDX1 cells. Samples are normalized to Day 0, data represent the mean and SEM of three independent replicates.

B–D Immunoblot of Tubulin and PRDX1 in U2-OS WT and sgPRDX1 (B), U2-OS shControl and shPRDX1 cell populations (C), and HT1080 WT and PRDX1$^{-/-}$ cells (D).

E Competitive growth assay of HT1080 WT and PRDX1$^{-/-}$ cells. Samples are normalized to Day 0, data represent the mean and SEM of six independent replicates.

F Detection of apoptosis in U2-OS WT and sgPRDX1 cells using Annexin V-Propidium Iodide staining. Data represent the mean and SEM of three independent replicates.

G Visualization of γH2AX (in red) within DAPI stained nuclei (in blue) in U2-OS sgPRDX1 and HT1080 PRDX1$^{-/-}$ cells. Cells positive for γH2AX are indicated with a white arrow. Cells were untreated and images were acquired on an Opera High Content Screening System, the scale bar is 20 μm.

H Quantification of images shown in (G), represented as the percentage of cells in each bin of γH2AX foci number. A minimum of 445 cells were quantified for each condition and replicate, using CellProfiler. Data represent the mean and SEM of five or six independent replicates for U2-OS or HT1080 cells, respectively.

I Visualization of the DNA damage marker γH2AX (in green) and the telomere markers TRF2 in HT1080 PRDX1$^{-/-}$ cells, respectively (in red). Cells were untreated and images were acquired on an Olympus spinning disk confocal microscope, the scale bar is 20 μm.

J Visualization of the replication stress markers pRPA32 and PCNA in HT1080 PRDX1$^{-/-}$ cells. Cells positive for pRPA32 or PCNA are indicated with a white arrow. Cells were untreated and images were acquired on an Opera High Content Screening System, the scale bar is 20 μm.

K Quantification of immunofluorescence images after staining of the replication stress markers RPA32, RPA70, and pATR, in HT1080 WT and PRDX1$^{-/-}$ cells. A minimum of 375 cells for RPA32 staining, 900 cells for RPA70 staining, and 180 cells for pATR staining were quantified for each condition and replicate, using CellProfiler. Data represent mean and SEM of four independent replicates for RPA32 and pATR stainings and three independent replicates for RPA70 staining. *P*-values were calculated using paired *t*-test where ns: not significant ($P > 0.05$), *$P < 0.05$, **$P < 0.01$, ***$P < 0.001$, ****$P < 0.0001$.

L Quantification of percentage γH2AX positive cells in HT1080 WT or PRDX1$^{-/-}$ cells, either untreated (UT) or treated for 3 days with the 2 mM aspartate. Three independent biological replicates are represented. *P*-values were calculated using the Student's *t*-test where ns: not significant ($P > 0.05$), *$P < 0.05$, **$P < 0.01$, ***$P < 0.001$, ****$P < 0.0001$.

M Quantification of the percentage of pRPA32-positive cells in HT1080 WT or PRDX1$^{-/-}$ cells, either untreated (UT) or treated for 3 days with 2 mM aspartate. A minimum of 300 cells were quantified for each condition and replicate, using CellProfiler. Data represent the mean and SEM of three independent replicates. *P*-values were calculated using paired *t*-test where ns: not significant ($P > 0.05$), *$P < 0.05$, **$P < 0.01$, ***$P < 0.001$, ****$P < 0.0001$.

N Competitive growth assay of HT1080 WT and PRDX1$^{-/-}$ cells after 2 mM aspartate treatment. Samples are normalized to Day 0, data represent the mean and SEM of three independent replicates.

© 2023 The Authors
