# Supplementary material for: A metabolic map of the DNA damage response identifies PRDX1 in the control of nuclear ROS scavenging and aspartate availability
Source: Mol Syst Biol. 2023 Jun 1;19(7):e11267. doi: 10.15252/msb.202211267 (PMC10333845; doi:10.15252/msb.202211267)
Supplement: Supplementary file 11 — PDF+ [file MSB-19-e11267-s010.pdf]

Article

TRANSPARENT PROCESS
OPEN ACCESS

molecular systems biology

A metabolic map of the DNA damage response identifies PRDX1 in the control of nuclear ROS scavenging and aspartate availability

Amandine Moretton[1,2,†], Savvas Kourtis[3,†], Antoni Gañez Zapater[3,‡], Chiara Calabrò[1,‡], Maria Lorena Espinar Calvo[3], Frédéric Fontaine[2], Evangelia Darai[3], Etna Abad Cortel[4], Samuel Block[5], Laura Pascual-Reguant[3], Natalia Pardo-Lorente[3], Ritobrata Ghose[3], Matthew G Vander Heiden[5,6,7], Ana Janic[4], André C Müller[2], Joanna I Loizou[1,2,*] & Sara Sdelci[3,**]

Abstract

While cellular metabolism impacts the DNA damage response, a systematic understanding of the metabolic requirements that are crucial for DNA damage repair has yet to be achieved. Here, we investigate the metabolic enzymes and processes that are essential for the resolution of DNA damage. By integrating functional genomics with chromatin proteomics and metabolomics, we provide a detailed description of the interplay between cellular metabolism and the DNA damage response. Further analysis identified that Peroxiredoxin 1, PRDX1, contributes to the DNA damage repair. During the DNA damage response, PRDX1 translocates to the nucleus where it reduces DNA damage-induced nuclear reactive oxygen species. Moreover, PRDX1 loss lowers aspartate availability, which is required for the DNA damage-induced upregulation of *de novo* nucleotide synthesis. In the absence of PRDX1, cells accumulate replication stress and DNA damage, leading to proliferation defects that are exacerbated in the presence of etoposide, thus revealing a role for PRDX1 as a DNA damage surveillance factor.

Keywords aspartate metabolism; DNA damage response; electron transport chain; Peroxiredoxin 1; reactive oxygen species scavenging
Subject Categories DNA Replication, Recombination & Repair; Metabolism
DOI 10.15252/msb.202211267 | Received 1 August 2022 | Revised 8 May 2023 | Accepted 10 May 2023 | Published online 1 June 2023
Mol Syst Biol. (2023) 19: e11267

Introduction

Maintaining genome integrity via the repair of DNA damage is a key biological process required to suppress diseases, including growth retardation, malignancy, neurodegeneration, and congenital anomalies (Jackson & Bartek, 2009). DNA is continually subjected to both exogenous and endogenous mutagens and hence cells have evolved distinct DNA repair mechanisms to counter different types of DNA damage (Hoeijmakers, 2001). In response to DNA damage, cells elicit a signaling cascade to repair the damaged DNA and/or arrest the cell cycle. The cascade results in the activation of specific repair machinery, which is recruited to the relevant site on chromatin. If the damage is beyond repair, sustained signaling from the damaged site may promote cells to enter senescence or undergo apoptosis.

Recent years have seen remarkable progress in unraveling the mechanisms of the DNA damage response, broadening our knowledge of the diverse DNA damage response pathways. Through such work, it has emerged that cellular metabolism not only generates DNA damage but also affects DNA repair (Turgeon *et al*, 2018; Moretton & Loizou, 2020). Metabolic reactions give rise to diverse types of DNA damage. Reactive oxygen species (ROS), mainly produced by oxidative phosphorylation, induce oxidative DNA damage, which is prevented by antioxidant metabolites such as glutathione (GSH) and nicotinamide adenine dinucleotide phosphate (NADPH; Dizdaroglu, 1992; Harris *et al*, 2015). By-product metabolites such as aldehydes and alkylating agents can also form toxic adducts on DNA (Nakamura *et al*, 2014). Another aspect of the crosstalk between

1 Center for Cancer Research, Comprehensive Cancer Center, Medical University of Vienna, Vienna, Austria
2 CeMM Research Center for Molecular Medicine of the Austrian Academy of Sciences, Vienna, Austria
3 Centre for Genomic Regulation (CRG), The Barcelona Institute of Science and Technology, Barcelona, Spain
4 Department of Medicine and Life Sciences, Universitat Pompeu Fabra, Barcelona, Spain
5 Koch Institute for Integrative Cancer Research, Massachusetts Institute of Technology, Cambridge, MA, USA
6 Department of Biology, Massachusetts Institute of Technology, Cambridge, MA, USA
7 Dana-Farber Cancer Institute, Boston, MA, USA
*Corresponding author. Tel: +44 7385978020; E-mail: joanna_loizou@hotmail.com
**Corresponding author. Tel: +34 933160149; E-mail: sara.sdelci@crg.eu
†These authors contributed equally to this work as first authors
‡These authors contributed equally to this work as second authors

© 2023 The Authors. Published under the terms of the CC BY 4.0 license.

cellular metabolism and the DNA damage response is the tight control of the metabolic reactions involved in nucleotide synthesis. This is necessary for maintaining genomic integrity, thus avoiding replication stress and nucleotide misincorporations, and ensuring efficient DNA repair through the production of a local pool of nucleotides, within the vicinity of DNA double-strand breaks (DSBs; D'Angiolella *et al*, 2012; Buckland *et al*, 2014). The function and recruitment of DNA repair enzymes to chromatin can additionally be regulated by metabolic enzymes and metabolites. For instance, the dealkylases AlkB homologs 2 and 3 (ALKBH2/3), which repair DNA adducts, use α-ketoglutaric acid (α-KG)—produced from glutamine—as a key substrate and are inhibited by the oncometabolite 2-hydroxyglutarate (2HG; Wang *et al*, 2015; Tran *et al*, 2017). Finally, chromatin remodeling and epigenetic marks regulate the repair of DNA damage, especially DNA DSBs. Homologous recombination is promoted by histone acetylation, facilitated by the production of acetyl-CoA in the vicinity of DSBs (Sivanand *et al*, 2017). On the contrary, specific metabolites such as 2HG, fumarate, or succinate impair histone demethylation, preventing the recruitment of homologous recombination factors by inhibiting the lysine-specific demethylases 4A and 4B (KDM4A/B; Sulkowski *et al*, 2020).

Yet, despite accumulating evidence of the dynamic interplay between metabolic factors and the DNA damage response, there has not been a systematic, unbiased study aimed at addressing how metabolic perturbations affect DNA repair. Here, we have identified the consequences of metabolic alterations on DNA damage and repair using a range of systematic approaches. Metabolism-focused CRISPR-Cas9 functional genetic screens, chromatin proteomics, and targeted metabolomics following the induction of DNA damage using the chemotherapeutic Topoisomerase II inhibitor, etoposide, revealed the aspects of metabolism that are crucial for the maintenance of genome integrity. Our results indicate that loss of electron transport chain (ETC) enzymes is synthetically viable with etoposide and that some of the ETC enzymes are partially located on chromatin 24 h after etoposide release, concomitant with the increase in nuclear ROS. If nuclear ROS are generated following the induction of DSBs, Peroxiredoxin 1 (PRDX1) accumulates in the nucleus, where it is required for nuclear ROS clearance. Loss of PRDX1 alone increases nuclear Cytochrome c oxidase subunit 4 (COX4, subunit of the ETC complex IV), nuclear ROS, and γH2AX foci, features that are exacerbated in the presence of exogenous DNA damage. The cellular metabolome is also drastically perturbed following etoposide treatment and release, especially nucleosides and nucleoside-related metabolites. Additionally, the loss of PRDX1 substantially decreases aspartate levels, therefore limiting the ability of the cells to perform *de novo* nucleotide synthesis when required for DNA damage repair. Our multifaceted explorations identify PRDX1 as a DNA surveillance factor at the intersection of nuclear ROS scavenging and aspartate availability.

Results

Genetic map of metabolic factors that impact the DNA damage response

A thorough characterization of DNA damage response-associated metabolic requirements has not yet been achieved. To study the impact of metabolic alterations on the DNA damage response, we performed a CRISPR-Cas9 genetic screen to identify metabolic genes that affect cellular survival in response to DNA damage. We used a sgRNA library targeting metabolism-related genes, including metabolic enzymes, small molecule transporters, and metabolism-related transcription factors (Birsoy *et al*, 2015). We transduced the human cell line U2-OS with the sgRNA library and subsequently induced DSBs using etoposide, a common chemotherapeutic drug that inhibits Topoisomerase II (Hande, 1998). After 9 days, cells were exposed to 1 μM etoposide for 3 h followed by 5 days of release (denoted "survival CRISPR screen"), and untreated cells were cultured in parallel as a control (Fig 1A). The performed treatment allowed for the clearance of DNA damage 24 h postrelease, as shown by the restoration of γH2AX, a double-strand DNA damage marker (Sharma *et al*, 2012), to basal levels (Fig EV1A). DNA was extracted from treated and untreated cells and mapped to the reference genome (Fig EV1B–D, Dataset EV1). As part of the data analysis, a cell cycle normalization step was performed to compensate for cell cycle defects that might occur due to the etoposide treatment (Fig EV1E and F). Depleted sgRNAs allowed for the identification of metabolic genes that are required for cell survival upon etoposide treatment (synthetic lethal), while accumulated sgRNAs allowed for the identification of genes whose loss is synthetic viable with etoposide treatment (Fig 1B). Hypoxia-Inducible Factor 1 Subunit Alpha (HIF1A) and Aryl Hydrocarbon Receptor Nuclear Translocator (ARNT, also known as HIF1B), which interact to form the HIF1 heterodimeric transcription factor that promotes pro-glycolytic transcriptional states (Kim *et al*, 2006) were identified as potent synthetic lethal targets (Fig 1B). The formation of the HIF1A-HIF1B heterodimer depends on HIF1A stabilization, which is commonly driven by hypoxia (Semenza, 2007), accumulation of ROS (Movafagh *et al*, 2015), and nutrient deprivation (Nishimoto *et al*, 2014), among other conditions. It is noteworthy that HIF1A mediates etoposide resistance in hypoxia conditions (Hussein *et al*, 2006).

Conversely, unbiased KEGG-based gene set enrichment analysis (GSEA) of the survival CRISPR screen revealed that many genes of the Tricarboxylic Acid Cycle (KEGG term Citrate cycle [TCA cycle]) and the ETC (KEGG term Chemical Carcinogenesis—ROS), which are essential for oxidative phosphorylation and cellular respiration (Kanehisa & Goto, 2000; Wu *et al*, 2021), were synthetically viable upon etoposide treatment (Fig 1B). Etoposide treatment generates ROS, which contribute to the cytotoxicity of this drug and arise from increased mitochondrial mass and respiration (Shin *et al*, 2016). ROS are important signaling molecules (Sies & Jones, 2020) that are physiologically produced during oxygen-consuming reactions in the mitochondria due to leaking electrons in the ETC, which cause partial oxygen reduction into superoxide radicals that are converted into H_2O_2 and hydroxyl radicals (Giorgio *et al*, 2007). Increased mitochondrial mass and respiration can result in increased ROS levels and HIF1A stabilization, which in turn leads to the downregulation of mitochondrial respiration (Yao *et al*, 2019). Using a fluorogenic probe to measure DNA-associated ROS, we observed that upon treating cells with a low etoposide concentration there was increased mitochondrial ROS, especially 24 h after etoposide release (Figs 1C and D left and EV1G left). This effect was accompanied by an increase in mitochondrial mass, detected with Mitotracker, which was moderate after etoposide treatment (Fig EV1G middle) and clearly significant at 24 h postetoposide release (Fig 1C and D

© 2023 The Authors

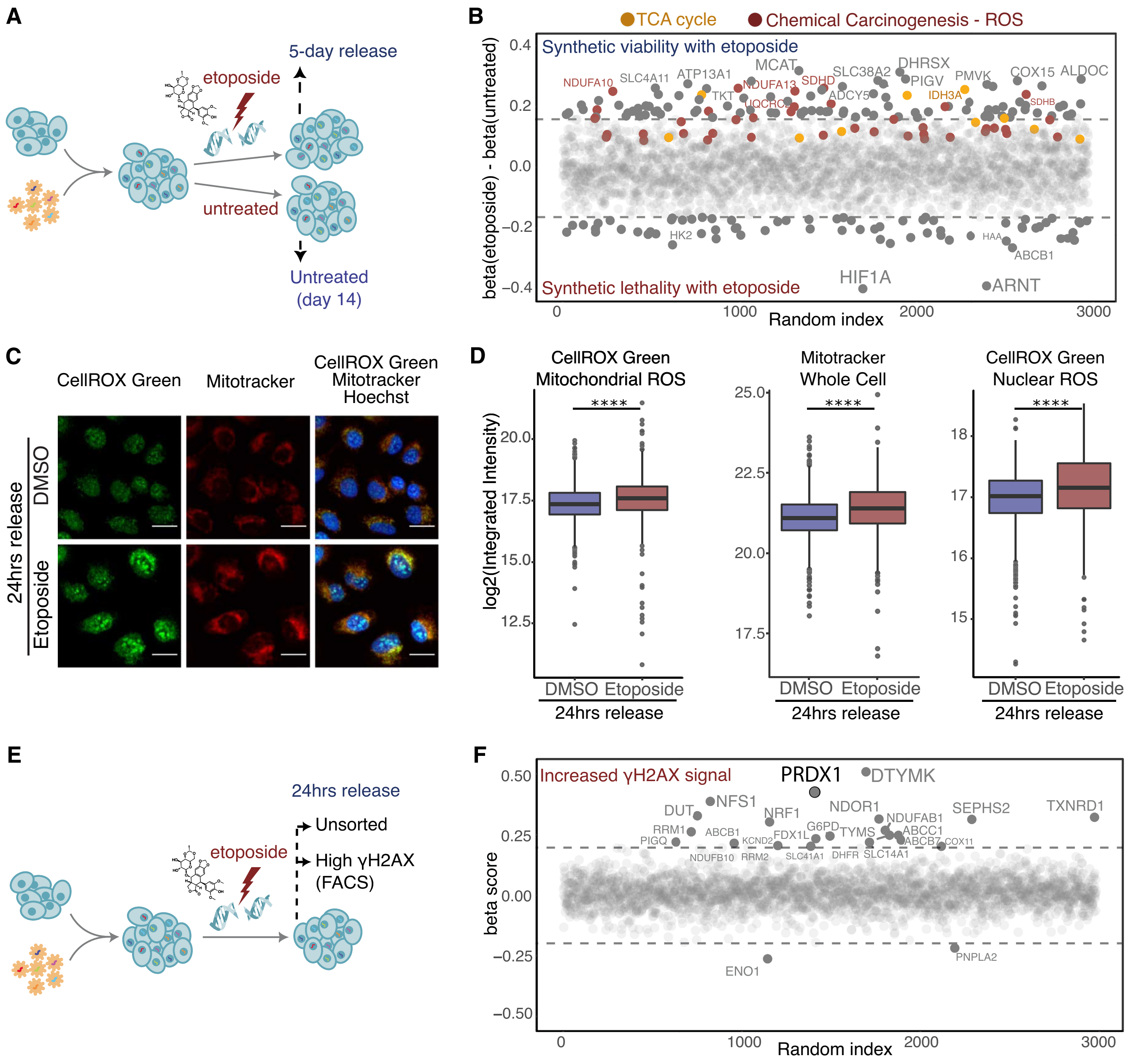

Figure 1. Metabolism-wide CRISPR-Cas9 screens identify ROS-related genes as synthetically viable with etoposide treatment.

A Schematic representation of the etoposide survival CRISPR-Cas9 screen. Cells were treated with 1 μM of etoposide for 3 h and allowed to recover for 5 days.

B Genes synthetic lethal with etoposide survival are represented by negative β scores. Genes contributing to significant enrichment of KEGG terms are colored. The sizes of the labels represent the relative significance of screen hits.

C Visualization of ROS (CellROX Green, in green) and mitochondria (Mitotracker, in red) within Hoechst-stained nuclei (in blue) in U2-OS WT cells in DMSO treated and 24 h etoposide release conditions. Images were acquired on an Operetta High Content Screening System in confocal mode, scale bar is 25 μm.

D Quantification of images shown in (C), represented as log2 integrated intensity. Three biological replicates were performed. A minimum of 1,000 cells were quantified for each condition, using Harmony. Boxplots represent the median within the IQR. *P*-values were calculated using linear regression on the log2 normalized values (ns: not significant ($P > 0.05$), *$P < 0.05$, **$P < 0.01$, ***$P < 0.001$, ****$P < 0.0001$).

E Schematic representation of the etoposide high-γH2AX CRISPR-Cas9 screen.

F Genes necessary for γH2AX clearance are represented by positive β scores. The sizes of the labels represent the relative significance of screen hits.

middle). The augmented mitochondrial ROS levels can be the direct consequence of the mitochondrial mass increase (Fig EV1H). Additionally, following etoposide treatment, we observed a significant increase in nuclear ROS, which was already present after etoposide treatment (Fig EV1G right) and became more pronounced at 24 h postetoposide release (Fig 1C and D right). Taken together, the results of this genetic screen indicate that cells with a heightened glycolytic phenotype better tolerate DNA damage. Indeed, the

treatment with low-dose of Phenformin and Thenoyltrifluoroacetone (TTFA; which target, respectively, ETC Complex 1 and Complex 2) tended to increase cell survival of etoposide-treated cells (Fig EV1I), thereby validating the results of our survival screening.

We reasoned that 5 days of release postetoposide treatment would hamper the identification of metabolic genes that function early in the DNA damage response. Thus, for identifying genes that affected levels of DNA damage, we FACS sorted high-γH2AX cells after 24 h of etoposide release (denoted "high-γH2AX CRISPR screen"; Figs 1E and EV1J) and extracted the DNA. The quality control for this approach was performed as for the survival-CRISPR screen (Fig EV1K and L). Enolase 1 (ENO1) and Patatin-Like Phospholipase Domain Containing 2 (PNPLA2) were the only two genes for which we found significantly depleted sgRNAs in the γH2AX high population (Fig 1F, Dataset EV1). This limited number of significantly depleted genes suggested that the lack of γH2AX clearance 24 h postetoposide treatment did not depend on the enzymatic activity of any particular metabolic process. In fact, ENO1 downregulation attenuates DNA damage induced by doxorubicin independently of its enzymatic activity (Gao *et al*, 2015).

Our high-γH2AX CRISPR screen revealed that the loss of several genes involved in nucleotide metabolism, such as Deoxythymidylate Kinase (DTYMK), Deoxyuridine Triphosphatase (DUT), Ribonucleotide Reductase Catalytic Subunit M1/2 (RRM1/2), Dihydrofolate Reductase (DHFR), and Thymidylate Synthetase (TYMS) was associated with a lack of γH2AX clearance. sgRNAs targeting membrane transporters with known multidrug-resistance functions, such as ATP Binding Cassette Subfamily C Member 1 (ABCC1), ATP Binding Cassette Subfamily C Member 7 (ABCB7), and ATP Binding Cassette Subfamily B Member 1 (ABCB1) also induced retention of γH2AX. Additionally, we observed that the depletion of genes with oxidoreductase activity, such as Peroxiredoxin 1 (PRDX1), Thioredoxin Reductase 1 (TXNRD1), NADPH Dependent Diflavin Oxidoreductase 1 (NDOR1), and Glucose-6-Phosphate Dehydrogenase (G6PD), which have a fundamental role in ROS balancing, were also associated with a lack of γH2AX clearance. In particular, PRDX1 displayed the most pronounced phenotype, indicating a strong connection between this enzyme and γH2AX clearance (Fig 1F, Dataset EV1).

To validate the results of the high-γH2AX CRISPR screen, we performed an arrayed CRISPR screen using a library targeting the top genes whose depletion led to the retention of γH2AX 24 h after etoposide or radiomimetic compound neocarzinostatin (NCS) release. We treated cells with NCS (60 ng/ml) for 1 h to allow DNA damage clearance, as shown by the clearance of γH2AX staining following 20 h of release (Fig EV1M). As expected, targeting nucleotide metabolism-related genes strongly promoted the accumulation of γH2AX foci even in the absence of exogenous DNA damage. Targeting selected oxidoreductases (NDOR1, G6PD, TXNRD1, and PRDX1) did not induce a dramatic increase in γH2AX foci but impeded the clearance of DNA damage 20 h post-DSBs induction, indicating that these proteins might function in the DNA damage response (Fig EV1N).

Metabolic enzymes involved in DNA damage response localize on chromatin

We observed a marked increase in ROS within the cell nucleus 24 h postetoposide release (Fig 1C and D right). We hypothesized that metabolic enzymes involved in ROS scavenging must be required in the nucleus to allow ROS clearance. To test this hypothesis, we studied changes in the composition of the chromatin-associated proteome in response to DNA damage. U2-OS cells were treated with DMSO or 1 μM etoposide for 3 h. Treated cells were either harvested or released into drug-free media to allow for the monitoring of proteins bound to chromatin up to 24 h postrelease (Fig 2A). Chromatin-bound proteins (chromatome) were extracted and analyzed by mass spectrometry (MS). Data analysis included batch correction (Fig EV2A) and normalization (Fig EV2B). The purity of the chromatomes was assessed by checking the relative enrichment of protein in different cellular compartments, showing strong enrichment for chromatin-related proteins and depletion in cytoplasmic and secretory proteins (Fig EV2C–E).

We identified in total 2,950 chromatin-bound proteins, of which 600 were metabolic factors, as annotated by the metabolic CRISPR library (Birsoy *et al*, 2015) and the Metabolic Atlas (Robinson *et al*, 2020; Fig EV2F, Datasets EV2 and EV3). The metabolic CRISPR library comprises metabolic enzymes, small molecule transporters, and metabolism-related transcription factors, while the Metabolic Atlas dataset broadly encompasses proteins involved in human enzymatic reactions. Eighty proteins were differentially enriched or depleted on chromatin immediately after etoposide treatment (Fig 2B) and after 24 h of etoposide release (Fig 2C). The chromatome composition remained altered 24 h postrelease (Fig EV2A), despite the strong reduction in the γH2AX-positive cells at this time point (Fig EV1A). This observation indicated that regardless of the presence of γH2AX, 24 h after etoposide release chromatin-associated alterations did not recover to their basal state. Validating our results, several known DNA repair factors, (e.g., RAD18, BRCA1, BARD1, and DNAJC2), were differentially recruited to chromatin following etoposide treatment and release. Among these, Topoisomerase II alpha (TOP2A), the target of etoposide that forms covalent TOP2-DNA cleavage complexes, accumulated on chromatin upon etoposide release (Figs 2B and C, and EV2G), supporting the relevance of our chromatome- DNA damage response proteomics dataset. Additionally, cell cycle genes (e.g., PCNA and CDC26) were depleted from chromatin 24 h postrelease (Figs 2B and C, and EV2G), potentially due to a reduction in cellular proliferation and partial cell cycle arrest following DSB induction (Fig EV2H).

Among the significantly altered proteins, we identified 11 metabolic enzymes (Dataset EV2 "chromatin- diff_Metabolic" sheet). We observed that several metabolic factors identified as differentially enriched or depleted in our genetic screens were found on chromatin (Dataset EV4), or are known to have chromatin interactors (Fig 2D), suggesting that in response to DNA damage, these proteins may have nuclear functions. Among them, Holocytochrome C Synthase (HCCS) that is required for the maturation of cytochrome C and the transfer of electrons between the ETC Complexes, Carbamoyl-Phosphate Synthetase 2-Aspartate Transcarbamylase-Dihydroorotase (CAD) that is essential for *de novo* pyrimidine synthesis, and PRDX1 that scored highly in our functional screen (Fig 1F), and whose depletion resulted in the lack of γH2AX clearance following etoposide and NCS treatments (Fig EV1N). PRDX1 is a thiol-specific peroxidase that prevents the accumulation of ROS in cells and the generation of oxidative damage, thus functioning in H_2O_2-mediated signaling and cell growth upon oxidative stress (Neumann *et al*, 2009). Notably, we observed that the chromatin

 © 2023 The Authors

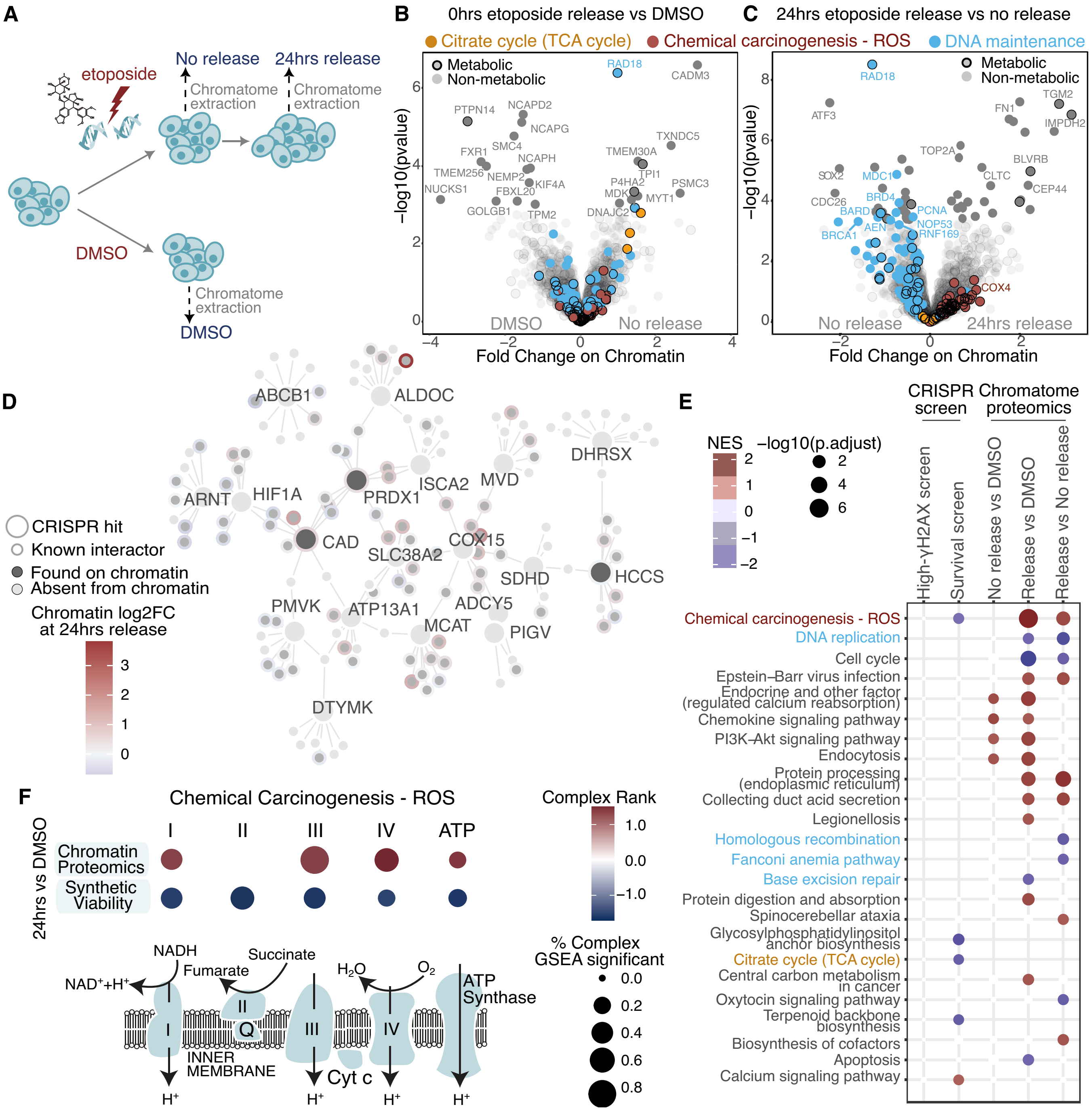

Figure 2. Chromatin proteomics reveals widespread accumulation of ROS-related genes on chromatin during etoposide release.

A Schematic representation of etoposide treatment and release of U2-OS cells followed by chromatin extraction and DIA-MS acquisition.

B, C Significant changes in protein abundance on chromatin upon etoposide treatment (B) compared to DMSO, and upon 24-h release (C) compared to no release. Genes contributing to significant enrichment of KEGG terms are colored. Genes are considered to have a metabolic function if they are either in the CRISPR metabolic library or in the Metabolic Atlas. Proteins with an adjusted *P*-value lower than 0.05, were considered significant hits. More than three biological replicates were performed.

D Protein–protein interaction network for top 5% gene hits in the CRISPR-Cas9 screens and their fold change on chromatin upon etoposide release. Proteins detected on chromatin are shown in dark gray if they were also CRISPR hits, or in lighter gray, if they are interactors of CRISPR hits.

E Overlap of significant KEGG terms between the CRISPR-Cas9 screens and chromatin proteomics. The directionality of the screen was reversed for the red to represent essential genes for etoposide release survival.

F Mitochondrial electron transport chain genes significantly contributing to "Chemical Carcinogenesis – ROS" KEGG term in chromatin proteomics and survival CRISPR screen.

abundance of PRDX1 was slightly increased following etoposide treatment (Figs 2D and EV2I).

Finally, we performed KEGG-based GSEA with the datasets of the metabolism-focused CRISPR-Cas9 screens and chromatome proteomics (Fig 2E). DNA maintenance remained altered 24 h postetoposide release, as highlighted by the enrichment of terms related to DNA replication, homologous recombination, Fanconi Anemia, and base excision repair (grouped together under the term of DNA maintenance in Fig 2B and C). The "Chemical Carcinogenesis – ROS" term was shared between the survival CRISPR screen and the chromatome and was primarily defined by enzymes of the different ETC complexes. Of note, no enzyme from complex 2 was detected on chromatin (Fig 2F and Dataset EV5). Intriguingly, the majority of ETC enzymes that we found as chromatin-enriched upon etoposide treatment were also etoposide-synthetic viable (Dataset EV6) and their chromatin accumulation was highest 24 h after etoposide release (Figs 2C and EV2J), similar to nuclear ROS increase (Fig 1C and D right). Among the ETC complexes, Complex IV increased the most, with the subunit COX4 showing a clear chromatin increase following etoposide release (Fig EV2J).

PRDX1 depletion leads to augmented nuclear γH2AX, COX4 and ROS

Unexpectedly, we have detected mitochondrial COX4 on chromatin (Fig EV2D) and a clear chromatin COX4 increase 24-h postetoposide release (Fig EV2J). Leveraging on the Human Protein Atlas, we noticed a consistent COX4 nuclear localization in every tested cell line, in addition to its mitochondrial localization (Fig EV3A), counter-validating our observation (Thul *et al*, 2017). Using confocal microscopy, we detected COX4 in the nucleus of HCT116 and HEK-293 cells, even in the absence of exogenous DNA damage (Fig EV3B). Similarly, COX4 was found in the nucleus of U2-OS cells, where it was significantly increased 24 h after etoposide release (Fig 3A and B), as observed for nuclear ROS levels (Fig 1C and D right). The antibody used in the Human Protein Atlas (Sigma-Aldrich) is different from the ones used by us for either HCT116 and HEK-293 staining (Abcam), or U2-OS staining (Thermofisher). The latter showed a marked nuclear localization, possibly indicating that it recognizes a variation of the COX4 protein that tends to localize in the nuclear compartment. To check for the specificity of this antibody, we used two independent shRNA-targeting COX4 and with each, we detected a strong reduction of the COX4 signal (Fig EV3C).

Given the increase in nuclear ROS observed 24 h after etoposide release (Fig 1C and D right) and the concomitant requirement for PRDX1 to eliminate etoposide-induced nuclear γH2AX foci (Fig 1F), we hypothesized that nuclear localization of PRDX1 may be necessary to reduce nuclear ROS levels after etoposide treatment, thus enabling DNA damage repair. Indeed, we observed that U2-OS PRDX1-depleted cells (shPRDX1) showed increased γH2AX even in absence of etoposide, and a significant increase in nuclear COX4 that was enhanced upon etoposide treatment (Fig EV3D and E) and release (Fig 3C and D). As previously observed (Egler *et al*, 2005), and in line with our hypothesis, PRDX1 depletion also triggered the accumulation of nuclear ROS, and mitochondrial mass increase, which, similar to COX4 (Fig 1A and B), significantly increased 24 h after etoposide release (Fig 3E and F). Smaller changes were observed immediately post-treatment (Fig EV4A and B). PRDX1 depletion was validated by Western blot and immunofluorescence (Fig EV4C). Together, these data suggest the presence of a functional connection between nuclear ROS accumulation, PRDX1 nuclear localization, and the presence of COX4 in the cellular nucleus.

Nuclear ROS drives PRDX1 nuclear recruitment

Next, we asked whether different DNA-damaging agents would induce nuclear PRDX1, COX4, and ROS accumulation. We answered this question by treating U2-OS cells with either the alkylating agent carboplatin (80 μM/3 h) or NCS (60 ng/ml/1 h). Carboplatin showed a delayed γH2AX increase that was not visible immediately after release, but was still present 24-h postrelease (Fig EV5A). However, similar to etoposide, it induced nuclear ROS and mitochondrial mass increase 24-h postrelease (Fig EV5B), which was accompanied by nuclear PRDX1 increase (Fig EV5C). NCS treatment failed to increase nuclear ROS, mitochondrial mass, and nuclear PRDX1 24-h post-treatment (Fig EV5D), even though it triggered γH2AX dynamics comparable to etoposide (Fig EV1M). These data indicate that an increase in nuclear ROS can drive PRDX1 nuclear recruitment, suggesting that it may act as a nuclear ROS scavenger.

We next observed that U2-OS PRDX1-depleted cells had elevated levels of γH2AX foci in basal conditions, and retained more γH2AX foci overtime, following etoposide treatment (Fig 4A). Interestingly, U2-OS PRDX1-depleted cells were slightly more sensitive to etoposide treatment, in agreement with the observed retention of DNA damage. Increased sensitivity was also observed when treating U2-OS PRDX1-depleted cells with carboplatin (Fig 4B).

Our results indicate that PRDX1 is important for nuclear ROS scavenging and that its depletion leads to γH2AX accumulation. It has been shown that PRDX1 loss leads to the inhibition of telomerase activity because of increased ROS-induced damage at telomeres (Ahmed & Lingner, 2018). We, therefore, investigated whether the loss of PRDX1 induced DNA damage specifically at telomeres. Co-staining of γH2AX with the telomere markers TRF1 and TRF2 in U2-OS PRDX1-deficient cells (sgPRDX1, Fig EV5E), showed that DNA damage was not restricted to telomeric regions (Fig 4C and D), indicating a broader role for PRDX1 in the DNA damage response.

To further validate PRDX1 nuclear localization and further study its association with DNA damage, we quantified γH2AX nuclear intensity together with PRDX1 nuclear intensity following etoposide treatment. We observed that γH2AX intensity increased immediately after treatment and nearly returned to baseline levels at 20 h of release (Fig 4E and F), which was in line with what we previously observed (Fig EV1A). In comparison, PRDX1 continued to accumulate in the nucleus (Fig 4E and F), potentially due to its role in scavenging etoposide-induced nuclear ROS, which reached a maximum after 24 h (Fig 1C and D right). The nuclear accumulation of PRDX1 was associated with a neglectable increase in the expression of the enzyme (Fig EV5F), suggesting relocalization rather than overall upregulation. Moreover, at 20 h of etoposide release, there was a correlation between nuclear accumulation of PRDX1 and high levels of γH2AX (Pearson coefficient of 0.84, Fig EV5G), indicating that cells with more damage also recruit more PRDX1 to the nucleus.

We did not observe PRDX1 nuclear foci that would confirm a direct interaction between PRDX1 and DNA damage on chromatin (Fig 4E). Therefore, the slight increase in PRDX1 abundance on chromatin following etoposide treatment (Fig EV2I) could instead

© 2023 The Authors

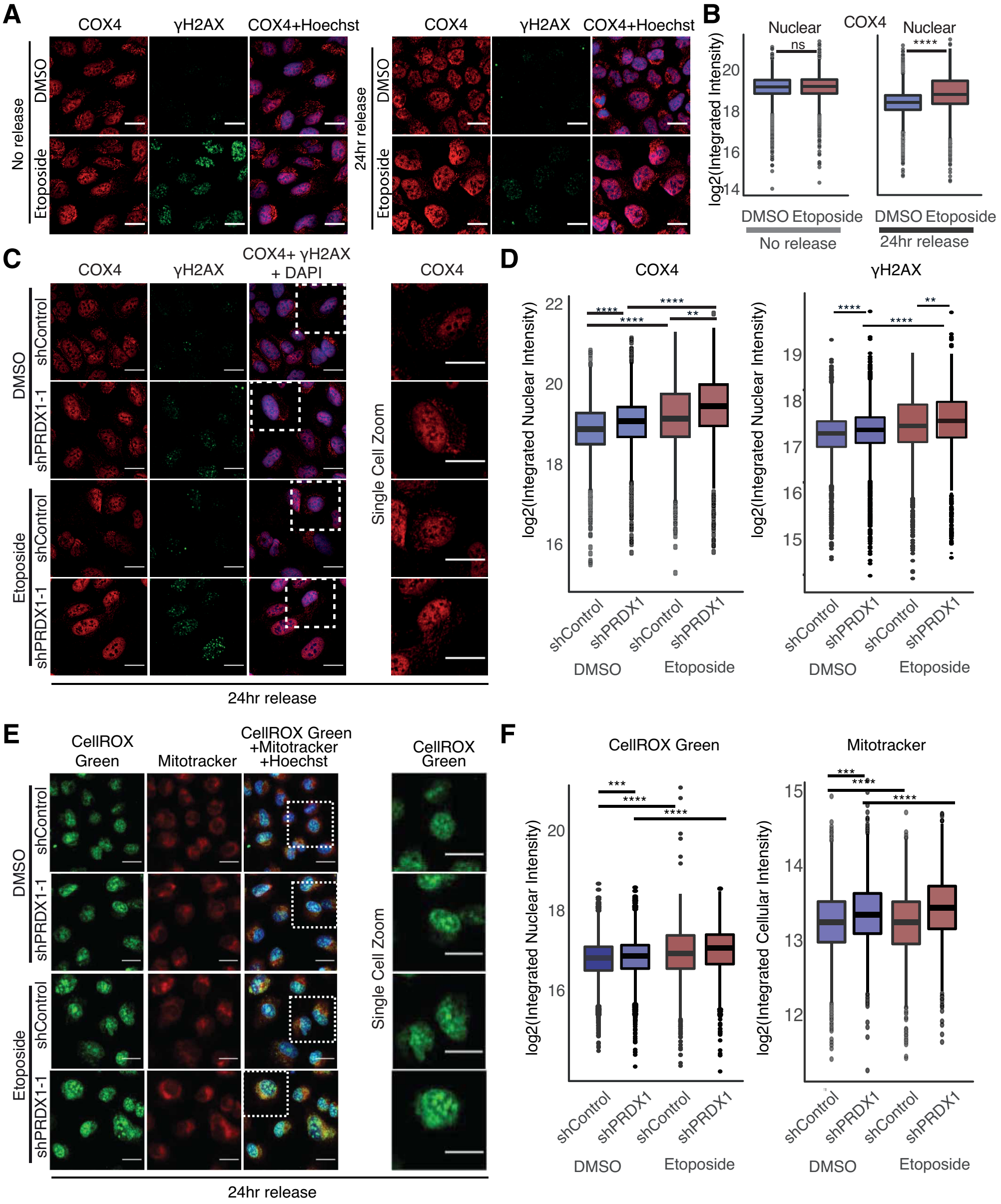

Figure 3. Nuclear COX4 and ROS accumulate in the absence of PRDX1.

A Visualization of COX4 (in red) and γH2AX (in green) within Hoechst stained nuclei (in blue) in U2-OS WT cells at the indicated treatment conditions. Images were acquired with the Operetta High Content Screening System in confocal mode, scale bar is 25 μm.

B Quantification of images shown in (A), represented as nuclear integrated intensities of COX4 signals. Three biological replicates were performed. A minimum of 1,000 cells were quantified for each condition, using Harmony. Boxplots represent the median with the IQR. *P*-values were calculated using a Student's *t*-test on the log2 normalized values (ns: not significant ($P > 0.05$), *$P < 0.05$, **$P < 0.01$, ***$P < 0.001$, ****$P < 0.0001$).

C Visualization of COX4 (in red) and γH2AX (in green) within DAPI stained nuclei (in blue) in U2-OS shControl and shPRDX1 cells at the indicated treatment conditions. Images were acquired with the Operetta High Content Screening System in confocal mode, scale bar is 25 μm.

D Quantification of images shown in (C), represented as nuclear integrated intensities of γH2AX and COX4 signals. Three biological replicates were performed. A minimum of 1,000 cells were quantified for each condition, using Harmony. Boxplots represent the median with the IQR. *P*-values were calculated using linear regression on the log2 normalized values (ns: not significant ($P > 0.05$), *$P < 0.05$, **$P < 0.01$, ***$P < 0.001$, ****$P < 0.0001$). The interaction term P-value between PRDX1 background and etoposide treatment is shown in the plot.

E Visualization of ROS (CellROX Green, in green) and mitochondria (Mitotracker, in red) within Hoechst-stained nuclei (in blue) in U2-OS shControl and shPRDX1 cells at the indicated treatment conditions. Images were acquired with the Operetta High Content Screening System in confocal mode, scale bar is 25 μm.

F Quantification of images shown in (E), represented as log2 nuclear-integrated intensity of CellROX Green and Mitotracker immediately at 24 h release compared to DMSO control. Three biological replicates were performed. A minimum of 1,000 cells were quantified for each condition, using Harmony. Boxplots represent the median with the IQR. *P*-values were calculated using linear regression on the log2 normalized values (ns: not significant ($P > 0.05$), *$P < 0.05$, **$P < 0.01$, ***$P < 0.001$, ****$P < 0.0001$). The interaction term *P*-value between PRDX1 background and etoposide treatment is shown in the plot.

© 2023 The Authors

represent a strong enrichment of PRDX1 in the nucleus. The relocalization of PRDX1 to the nucleus following etoposide treatment (Fig 4E and F), as well as the fact that PRDX1 levels influence the sensitivity to etoposide and carboplatin treatments (Fig 4B), strongly support the role of PRDX1 in the DNA damage response following exogenous DNA damage.

Since the DNA damage response is tightly linked to DNA replication and cell cycle progression, we queried when during the cell cycle PRDX1 localizes to the nucleus. We hypothesized that if PRDX1 is required for DNA damage surveillance, it would most likely be abundant in the nucleus during the G2 phase of the cell cycle when cells evaluate replication errors and eventually repair them. Using an adapted U2-OS FUCCI4 (Bajar *et al*, 2016) cell line stained for PRDX1, we observed that under basal conditions nuclear-PRDX1 was significantly higher in G2 cells (high green/high turquoise cells; Fig 4G and H) than in G1 cells (low green/high turquoise cells; Fig 4I), which is consistent with our hypothesis. Interestingly, it has been recently shown that ROS levels increase in a cell cycle-dependent manner, reaching maximum levels in G2 (Kirova *et al*, 2022). Contrastingly, cytoplasmic-PRDX1 followed an opposite trend, suggesting a cell cycle-dependent PRDX1 subcellular translocation (Fig 4J).

In summary, our results showed that PRDX1 accumulates in the nucleus when nuclear ROS levels are elevated, either after generation of DNA damage by etoposide or carboplatin treatment or during the G2 phase of the cell cycle, indicating that its nuclear localization is required for nuclear ROS scavenging.

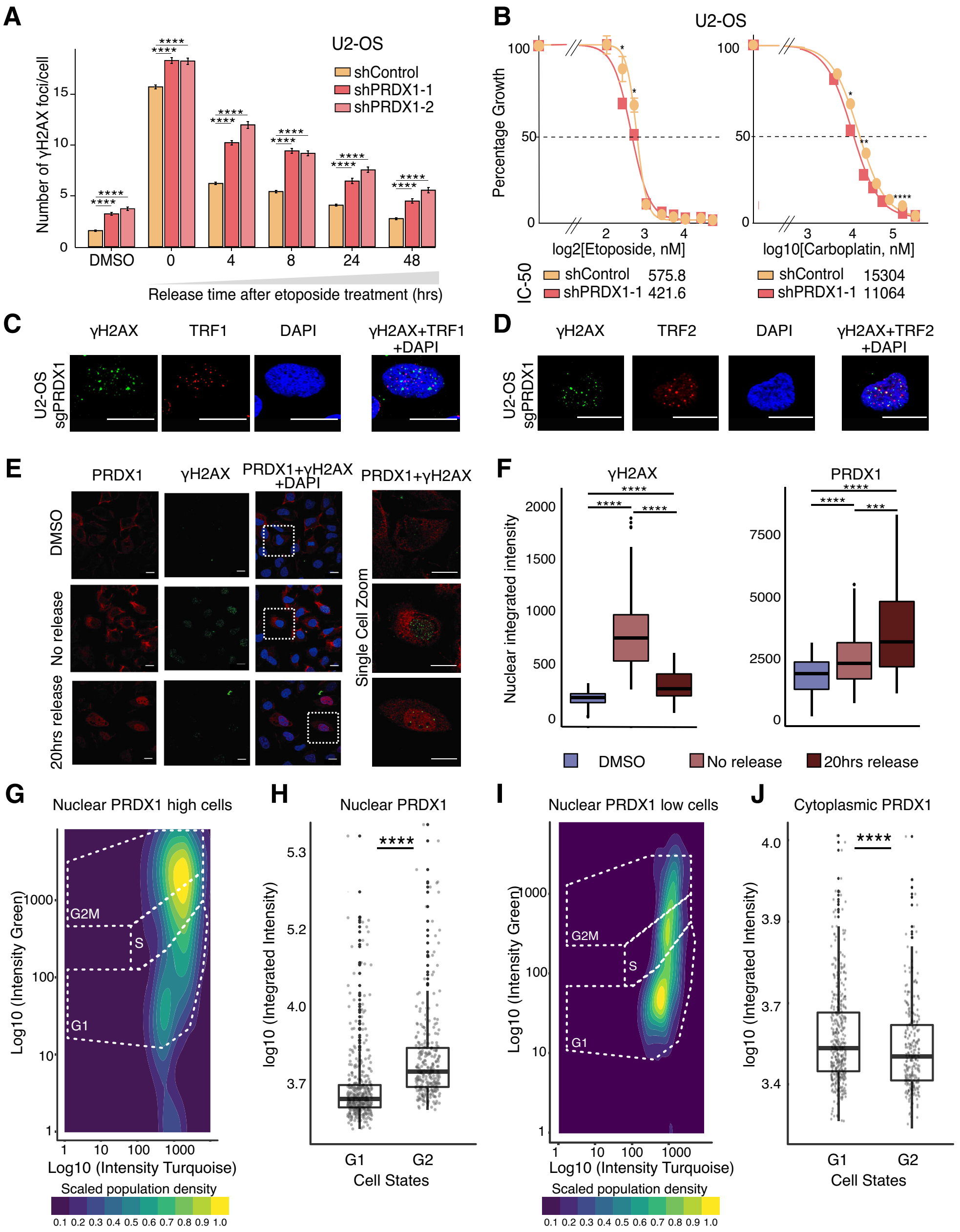

Figure 4.

 © 2023 The Authors

◀ **Figure 4. Nuclear ROS accumulates in the absence of PRDX1.**

A Kinetics of recovery after etoposide treatment in U2-OS shControl and shPRDX1 cells. Quantification of γH2AX immunofluorescence images represented as the mean number of γH2AX foci per nucleus. A minimum of 1,700 cells were quantified for each condition, using CellProfiler, from images acquired with an Opera High Content Screening System. Error bars represent SEM. *P*-values were calculated using the non-parametric Wilcoxon test where ns: not significant ($P > 0.05$), $*P < 0.05$, $**P < 0.01$, $***P < 0.001$, $****P < 0.0001$.

B Viability to increasing etoposide and carboplatin concentrations in U2-OS shControl and shPRDX1 cells. Three biological replicates were performed. Error bars represent SD. *P*-values were calculated using the *t*-test on three biological replicates where ns: not significant ($P > 0.05$), $*P < 0.05$, $**P < 0.01$, $***P < 0.001$, $****P < 0.0001$.

C, D Visualization of the DNA damage marker γH2AX (in green) and the telomere markers TRF1 (C) or TRF2 (D) in U2-OS sgPRDX1 (in red). Cells were untreated and images were acquired on an Olympus spinning disk confocal microscope, the scale bar is 20 μm.

E Visualization of PRDX1 (in red) and γH2AX (in green) within DAPI-stained nuclei (in blue) in U2-OS WT cells at the indicated treatment conditions. Images were acquired on a confocal Zeiss LSM800 microscope, scale bar is 20 μm.

F Quantification of images shown in (E), represented as nuclear integrated intensities of γH2AX and PRDX1 signals. Boxplots represent the median within the IQR of a minimum of 47 cells, quantified for each condition using CellProfiler. *P*-values were calculated using the non-parametric Wilcoxon test where ns: not significant ($P > 0.05$), $*P < 0.05$, $**P < 0.01$, $***P < 0.001$, $****P < 0.0001$.

G Cell cycle profile using the FUCCI4 system, with the scaled density of nuclear PRDX1 high cells.

H Quantification of nuclear integrated intensities of PRDX1 signals of images acquired with the Operetta High Content Screening System in confocal mode, and quantified using Harmony. Boxplots represent the median within the IQR for a minimum of 1,000 cells. Three biological replicates were performed. Cells were divided based on cell state and compared. *P*-values were calculated using a Student's *t*-test where ns: not significant ($P > 0.05$), $*P < 0.05$, $**P < 0.01$, $***P < 0.001$, $****P < 0.0001$.

I Cell cycle profile using the FUCCI4 system, with the scaled density of nuclear PRDX1 low cells.

J Quantification of cytoplasmic integrated intensities of PRDX1 signals of images acquired with the Operetta High Content Screening System in confocal mode, and quantified using Harmony. Boxplots represent the median within the IQR for a minimum of 1,000 cells. Three biological replicates were performed. Cells were divided based on cell state and compared. *P*-values were calculated using a Student's *t*-test where ns: not significant ($P > 0.05$), $*P < 0.05$, $**P < 0.01$, $***P < 0.001$, $****P < 0.0001$.

Metabolomics in the presence of DNA damage reveals that loss of PRDX1 compromises aspartate-dependent nucleotide synthesis

Since there is accumulating evidence linking metabolism and DNA damage, we assessed how the metabolic profile of cells is altered during the DNA damage response. To that end, we performed targeted metabolomics in U2-OS cells following etoposide treatment and release at different time points (Fig 5A). In total, 198 metabolites were measured, with a particular focus on nucleotide metabolism, amino acids, and organic acids (Dataset EV7). A total of 128 metabolites were detected in at least one sample, while 90 were consistently quantified in all samples (Fig EV6A), with missing values being more common for less abundant metabolites (Fig EV6B). The principal component analysis (PCA) plot showed a good clustering of biological replicates and indicated that, despite the reduction in the γH2AX signal (Fig EV1A), the cellular metabolome remained altered at 24 h of etoposide release compared to the basal state (Fig EV6C), in line with the findings from the chromatome dataset (Fig EV2A).

When comparing DMSO and etoposide-treated cells (Fig 5B), we observed that nucleosides and nucleoside-related metabolites were drastically perturbed. In particular, we identified that triphosphate nucleosides decrease immediately after etoposide treatment, and increase at 8 and 24 h of release. Conversely, monophosphate nucleoside levels were significantly increased at all time points but more pronounced immediately after etoposide treatment, suggesting that DSB induction and the activation of DNA damage response triggered *de novo* nucleotide synthesis. Similarly, ribose and xylulose 5-phosphate, which are required for the synthesis of nucleoside sugar rings, rapidly increased following etoposide treatment and returned to basal levels during release into drug-free media. This data suggested that nucleotides were acutely used upon DSB induction, probably as an outcome of repairing DNA damage, while during release into drug-free media, the pools of nucleotides were replenished via *de novo* nucleotide synthesis. Successful *de novo* nucleotide synthesis additionally requires glutamine and aspartate. Specifically, aspartate alone is required for *de novo* purine synthesis (Pareek *et al*, 2021), while glutamine is the precursor of carbamoyl-phosphate, which, together with aspartate, is required for carbamoyl-aspartate production and *de novo* pyrimidine synthesis (Del Cano-Ochoa *et al*, 2019). Moreover, carbamoyl-phosphate and aspartate also contribute to citrulline synthesis (Shi *et al*, 2018). We did not observe significant changes in aspartate or carbamoyl-aspartate levels, and carbamoyl-phosphate was not among the measured metabolites in our targeted approach. However, the reduction in citrulline observed at all given time points suggested that as an outcome of the DNA damage response, aspartate, and carbamoyl-phosphate are preferentially used for nucleotide synthesis.

The analysis of metabolites at the pathway level corroborated our observations. Pyrimidine synthesis was clearly upregulated 24 h after release. Conversely, the pentose phosphate pathway, which is required to synthesize the sugar backbone of nucleosides, was rapidly upregulated following etoposide treatment and decreased after release. Aspartate metabolism showed a similar behavior (Fig 5C). Overall, targeted metabolomics suggested that etoposide treatment activates *de novo* nucleotide synthesis, which 24 h after etoposide release still appears to be upregulated.

Given the fact that in our study PRDX1 was identified as having a central role in the DNA damage response, we investigated how the loss of PRDX1 might affect the cellular metabolic state following DNA damage. Therefore, we performed targeted metabolomics comparing U2-OS wild-type (WT) with the PRDX1-deficient cell population. In the PCA, PRDX1-deficient cells treated with DMSO overlapped with WT cells treated with etoposide, suggesting that loss of PRDX1 has an impact on the targeted metabolites (Fig EV6C). When investigating significantly altered metabolites by comparing DMSO-treated and etoposide-treated PRDX1-depleted

© 2023 The Authors

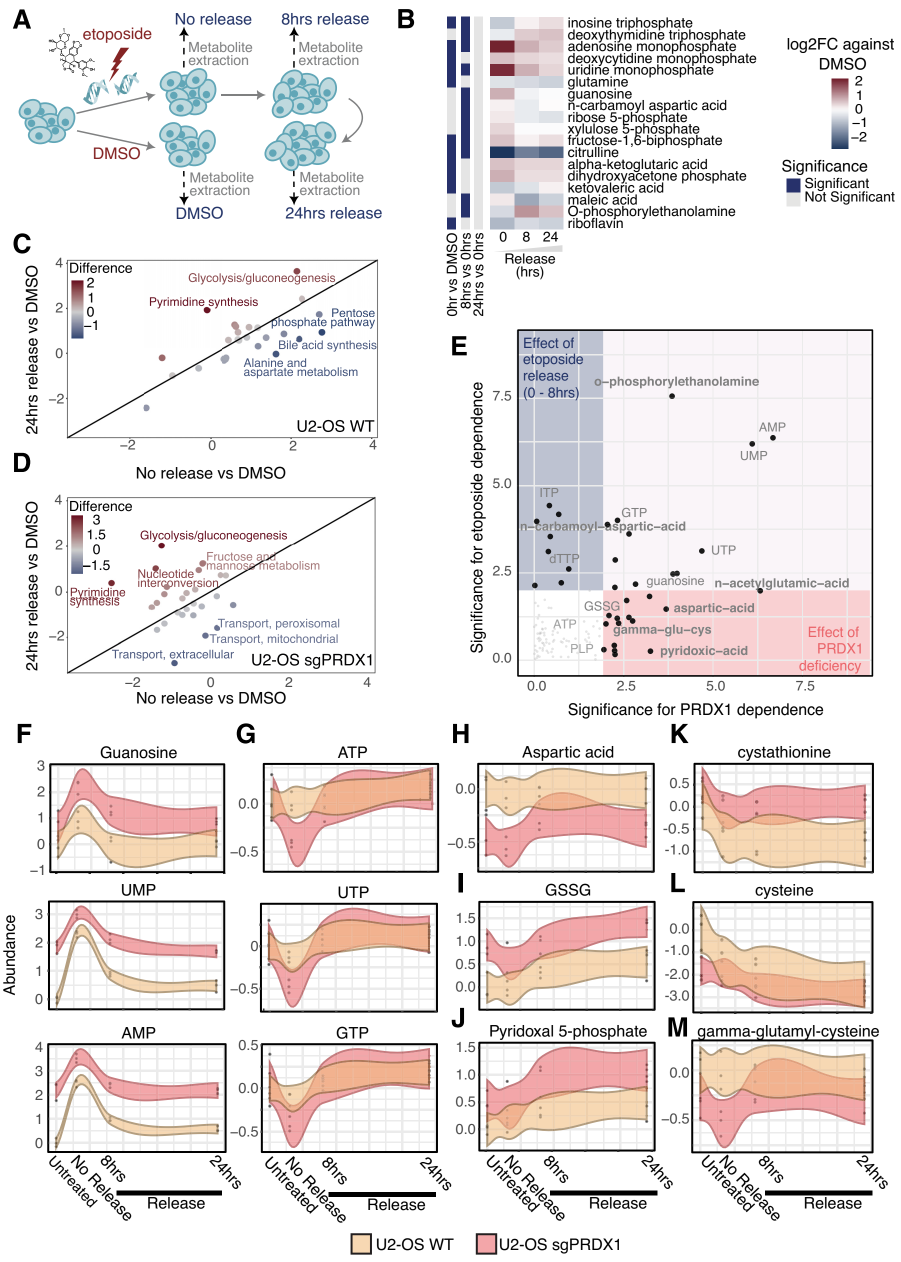

Figure 5. Cellular metabolome is drastically perturbed following etoposide treatment or PRDX1 loss.

A Schematic representation of etoposide treatment and release of U2-OS cells followed by metabolite extraction and LC–MS/MS acquisition.

B Relative abundances of metabolites that are significantly perturbed in at least one timepoint, represented as the log2 fold change compared to DMSO control.

C Metabolic pathways altered in U2-OS WT cells at 24 h of etoposide release vs DMSO compared to no release vs DMSO.

D Metabolic pathways altered in U2-OS sgPRDX1 cells at 24 h etoposide release vs DMSO compared to no release vs DMSO.

E PRDX1 deficiency-dependency and etoposide treatment-dependency of analyzed metabolites, based on linear regression analysis on the 0–8 h release time points.

F–M Abundance variations of example metabolites during the etoposide-release timecourse. Three biological replicates were performed. Shaded area represents the 95% confidence interval.

cells, we detected overall minimal oscillation (Fig EV6D), especially when compared with those observed in WT cells (Fig 5B). Interestingly, while triphosphate nucleotides decreased similarly to the WT cells upon etoposide treatment, mononucleotide increase was much smaller in the PRDX1-depleted cells, perhaps indicating that PRDX1-depleted cells are less proficient in replenishing their nucleotide pool. According to this, at the pathway level we observed that upon etoposide release, PRDX1-depleted cells did not upregulate pyrimidine synthesis at the same level as WT cells (Fig 5D).

 © 2023 The Authors

We, therefore, compared the metabolic contribution of either etoposide or PRDX1 loss in untreated or treated cells released for 0, 8, and 24 h. Guanosine, uridine, and adenosine monophosphate levels were influenced by both etoposide treatment and PRDX1 loss (Figs 5E and EV6E and F). However, upon etoposide treatment, their fluctuation in U2-OS WT cells was more pronounced suggesting a more efficient synthesis (Fig 5F). Monophosphate nucleotides in U2-OS PRDX1-deficient cells were consistently more abundant across time points; however, they did not fluctuate as much. Triphosphate nucleotide levels seemed to be mainly affected by etoposide treatment (Figs 5E and EV6E and F). However, the loss of PRDX1 clearly induced a sharper decrease upon DNA damage induction (Fig 5G). When looking at differential essential genes (preprint: Dempster *et al*, 2019) in PRDX1 low- and high-expressing cells, we observed that several genes of the folate pathway (green), the *de novo* purine metabolism (light pink), and the *de novo* pyrimidine metabolism (blue) were much more essential in the former. Thus, corroborating the hypothesis that PRDX1 loss may impact *de novo* nucleotide synthesis (Fig EV6G, Dataset EV8). Interestingly, aspartate levels, which are crucial for *de novo* synthesis of purines and pyrimidines, appeared to be strongly dependent on PRDX1 loss independently of etoposide treatment and release (Figs 5E and EV6E and F), being considerably decreased in PRDX1-depleted cells (Fig 5H).

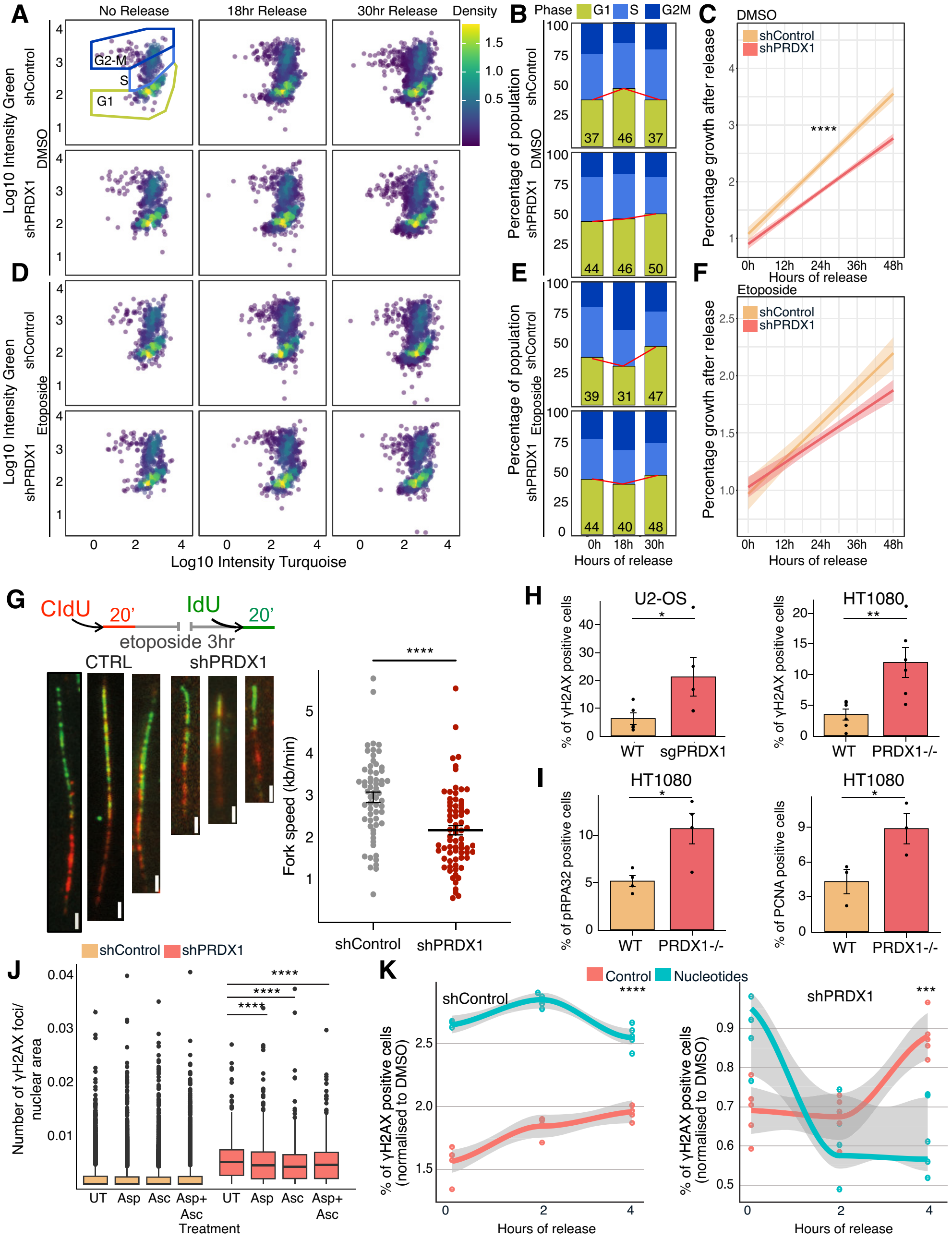

Figure 6.

Figure 6. PRDX1 loss-induced DNA damage is partially rescued by aspartate, ascorbic acid, and nucleotide supplementation.

A Cell cycle profile using the FUCCI4 system, with the density of cells, in U2-OS shControl and shPRDX1 cells, treated with DMSO or etoposide.

B Quantitation of percentages of cells in each cell cycle phase from (A), at the different DMSO-release time points.

C Quantitation of the number of U2-OS shControl or shPRDX1 cells in Fig 6A upon DMSO release, normalized to cells with 0hr release. *P*-values were calculated based on three biological replicates using the *t*-test where ns: not significant ($P > 0.05$), $^*P < 0.05$, $^{**}P < 0.01$, $^{***}P < 0.001$, $^{****}P < 0.0001$. Shaded area represents the 95% confidence interval and central line the median.

D Cell cycle profile using the FUCCI4 system, with the density of cells, in U2-OS shControl and shPRDX1 cells, treated with etoposide.

E Quantitation of percentages of cells in each cell cycle phase from (D), at the different etoposide-release time points.

F Quantitation of the number of U2-OS shControl or shPRDX1 cells in Fig 5D upon etoposide release, normalized to cells without release. *P*-values were calculated based on three biological replicates using the *t*-test where ns: not significant ($P > 0.05$), $^*P < 0.05$, $^{**}P < 0.01$, $^{***}P < 0.001$, $^{****}P < 0.0001$. Shaded area represents the 95% confidence interval and central line the median.

G Visualization and quantification of DNA replication fiber assay for U2-OS shControl and shPRDX1 cells pulsed with 25 μM CldU, treated with 1 μM etoposide for 3 h, and pulsed with 250 μM IdU. The scale bar is 2 μm. Data represent the mean and SD of cells combined from three biological replicates. *P*-values were calculated using paired *t*-test where ns: not significant ($P > 0.05$), $^*P < 0.05$, $^{**}P < 0.01$, $^{***}P < 0.001$, $^{****}P < 0.0001$.

H Quantification of images shown in (EV6G). A minimum of 445 cells were quantified for each condition and replicate, using CellProfiler. Data represent the mean and SEM of five or six biological replicates for U2-OS or HT1080 cells, respectively. *P*-values were calculated using paired *t*-test where ns: not significant ($P > 0.05$), $^*P < 0.05$, $^{**}P < 0.01$, $^{***}P < 0.001$, $^{****}P < 0.0001$.

I Quantification of stainings shown in (EV6H). A minimum of 500 cells for pRPA32 staining and 900 cells for PCNA staining were quantified for each condition and replicate, using CellProfiler. Data represent the mean and SEM of four or three biological replicates for pRPA32 or PCNA stainings, respectively. *P*-values were calculated using paired *t*-test where ns: not significant ($P > 0.05$), $^*P < 0.05$, $^{**}P < 0.01$, $^{***}P < 0.001$, $^{****}P < 0.0001$.

J Quantification of γH2AX foci/area of the nucleus in U2OS shControl or shPRDX1 cells, either untreated (UT) or treated with aspartate and/or ascorbate. Three biological replicates were performed. A minimum of 1,000 cells were quantified for each condition, using Harmony. Boxplots represent the median within the IQR. *P*-values were calculated using the Student's *t*-test where ns: not significant ($P > 0.05$), $^*P < 0.05$, $^{**}P < 0.01$, $^{***}P < 0.001$, $^{****}P < 0.0001$.

K Quantification of the percentage of γH2AX-positive cells in U2OS shControl or shPRDX1 cells, either treated with water or nucleotides (each at 100 μM). A minimum of 1,000 cells (from three biological-replicate wells) were quantified for each condition, using Harmony. *P*-values were calculated using the Student's *t*-test where ns: not significant ($P > 0.05$), $^*P < 0.05$, $^{**}P < 0.01$, $^{***}P < 0.001$, $^{****}P < 0.0001$. Shaded area represents the 95% confidence interval, and the central line the median.

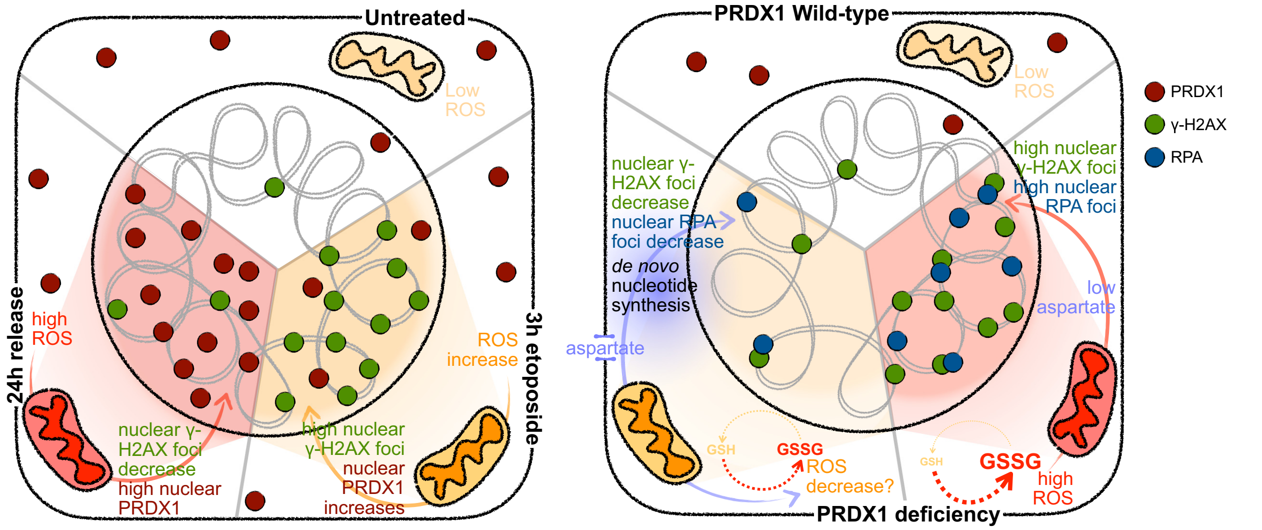

Figure 7. Schematic of PRDX1, DNA damaged-induced nuclear relocalisation.

Schematics of identified roles of PRDX1 in the DNA damage response.

Our experiments indicated that PRDX1-depleted cells have higher levels of mitochondrial and nuclear ROS in basal conditions and that ROS tend to accumulate even more upon etoposide treatment when PRDX1 is missing (Figs 3E and F, and EV4A and B). We, therefore, reasoned that in this scenario aspartate may be used for glutamate synthesis, which is essential for glutathione (GSH) synthesis and, consequently, cellular redox potential. Even though GSH was not among the detected metabolites in our dataset, its oxidized form, GSSG, was increased in the absence of PRDX1. Interestingly, GSSG levels further raised upon etoposide treatment and release, reaching their maximum at 24 h (Fig 5I), in agreement with the observed increase in mitochondrial and nuclear ROS levels (Fig 1C and D). The synthesis of GSH depends on transsulfuration reactions which mediate the interconversion of amino acids in the presence of the pyridoxal-5′-phosphate cofactor (PLP). One such reaction is, for example, the conversion of aspartate into glutamate mentioned above. While we did not observe changes in glutamate levels (Dataset EV7), PLP levels were PRDX1-status-dependent and always higher in PRDX1-deficient cells (Figs 5E and J, and EV6E and F). GSH synthesis proceeds with the addition of glutamate to cysteine

 © 2023 The Authors

to form gamma-glutamyl-cysteine, which is then converted into GSH with the addition of glycine. No changes were observed in glycine levels (Dataset EV7). However, the cysteine precursor cystathionine was upregulated in PRDX1-deficient cells (Fig 5K), while cysteine (Fig 5L) and gamma-glutamyl-cysteine (Fig 5M) were downregulated suggesting a faster flux toward GSH synthesis in the PRDX1-deficient cells. Interestingly, the conversion of cystathionine into cysteine also requires PLP, and Reactive Intermediate Imine Deaminase A Homolog (RIDA), an enzyme whose putative function is to prevent the inactivation of pyridoxal 5′-phosphate (PLP)-containing enzymes (Shen *et al*, 2022), showed a high essentiality score in PRDX1-low expressing cells (Fig EV6G, salmon). In the same category, we also retrieved the Glutamate-Cysteine Ligase Modifier Subunit (GCLM) and Glutamate-cysteine ligase catalytic subunit (GCLC; sky blue), which are required for the biosynthesis of GSH, with GCLC catalyzing the first and rate-limiting step in this process.

Finally, many genes involved in the cell cycle checkpoints (lilac) and homologous recombination (baby blue) scored as significantly more essential in PRDX1 low-expressing cells (Fig EV6G), corroborating the functional connection between PRDX1 and the DNA damage response.

Together, this data suggested that PRDX1 function is required to control the breakdown of intracellular aspartate levels between GSH synthesis and *de novo* nucleotide metabolism, an equilibrium that gets compromised during the DNA damage response due to the increase in ROS levels.

Supplementation of ascorbic acid, aspartate, and nucleotides partly rescues PRDX1 loss

Thus far, our results suggested PRDX1 at the center of the interplay between *de novo* nucleotide synthesis and nuclear ROS levels.

We reasoned that a slowdown of *de novo* nucleotide synthesis should affect cell proliferation. By mixing U2-OS PRDX1-deficient cells with U2-OS WT cells in equal amounts, we performed a competitive growth assay and determined the percentage of each cell population over a period of 12 days. U2-OS PRDX1-deficient cells decreased over time to 30–35% (Fig EV7A). However, we observed that the U2-OS PRDX1-deficient population recovered PRDX1 expression over time after knock-out generation (Fig EV7B), probably due to natural selection of PRDX1 heterozygous knock-outs or in-frame deletion clones. Similarly, shPRDX1-treated U2-OS partially restored PRDX1 expression after a few weeks in culture (Fig EV7C). Therefore, we repeated the competitive growth assay with a stable HT1080 PRDX1 knock-out clone (PRDX1$^{-/-}$; Fig EV7D). Here, the percentage of PRDX1-deficient cells dropped to 15% (Fig EV7E), indicating that the milder effect observed in U2-OS cells was most probably due to population heterogeneity.

We next questioned whether PRDX1 deficiency might impair cell survival. Annexin V-Propidium Iodide staining indicated that apoptosis did not increase in PRDX1-deficient cells when compared to PRDX1-WT cells (Fig EV7F), suggesting a more cytostatic effect of PRDX1 loss rather than cytotoxic. To investigate whether PRDX1 depletion impacts the cell cycle phase distribution, we employed our U2-OS FUCCI4 cell line system and showed that PRDX1-depleted cells have a G1 delay (Fig 6A and B), which could be the result of a reduction in aspartate levels and nucleotide synthesis capacity that may eventually lead to replication stress. Interestingly, while it was clear that the monitored control population was cycling, showing a G1 phase increase at 18 h followed by a subsequent decrease at 30 h, the shPRDX1 population showed minimal phase percentage variations indicative of a more static condition that resulted in less proliferation capacity (Fig 6A–C). When control cells were treated with 1 μM etoposide for 3 h and released, they showed an S-G2 delay at 18 h but they were able to recover and double in approximately 36 h. However, PRDX1-depleted cells showed a persistent G1-delay following etoposide treatment which was retained longer, increasing the doubling time of this population much beyond 48 h (Fig 6D–F). We reasoned that if etoposide increases nuclear ROS levels, simultaneous PRDX1 depletion might result in increased ROS-induced replication stress, which would reduce cell proliferation. DNA replication fiber assay indeed showed that PRDX1 depleted cells treated with 1 μM etoposide for 3 h had a significant reduction of DNA replication velocity as compared to control cells (Fig 6G), corroborating our hypothesis.

By immunofluorescence, we observed that, in the absence of etoposide treatment, U2-OS PRDX1-depleted and HT1080 PRDX1-deficient cells significantly accumulated γH2AX foci (Figs 6H and EV7G and H), which, also in the case of HT1080 cells, was not specifically localized at telomeres (Fig EV7I), as previously observed in U2-OS cells (Fig 4C and D). We, therefore, investigated whether the observed increase in DNA damage levels following PRDX1 loss could be in part explained by an accumulation of replication stress that, if not resolved, can lead to DSBs (Cortez, 2015). When analyzing the accumulation of replication stress markers (PCNA, pATR, RPA70, pRPA32; Essers *et al*, 2005; Soniat *et al*, 2019) in HT1080 PRDX1-deficient cells, we observed that, a subset of cells accumulated high levels of replication stress even in absence of etoposide (Figs 6I and EV7J and K).

We, therefore, questioned whether supplementation with selected metabolites could rescue DNA damage accumulation in PRDX1-depleted cells. From our metabolomics data, we hypothesized that PRDX1-depleted cells use aspartate in the attempt to rescue their GSH-GSSG altered ratio, thus reducing their nucleotide synthesis capacity. As a result, supplementation with ascorbic acid (antioxidant), aspartate, or their combination should decrease the DNA damage basal levels of those cells. Indeed, U2-OS PRDX1-depleted cells showed a significant decrease in the number of γH2AX foci with all the treatments (Fig 6J). A similar decrease in γH2AX foci and pRPA-positive cells was observed when treating HT1080 PRDX1-deficient cells with aspartate for 72 h (Fig EV7L and M). However, similar supplementation with aspartate failed to rescue the cellular proliferation defects even when PRDX1-deficient cells were supplemented for up to 12 days (Fig EV7N), indicating that PRDX1 influences cell growth through a multitude of mechanisms.

Finally, we reasoned that if the decreased aspartate availability induced by PRDX1 depletion affects nucleotide synthesis in presence of DNA damage, nucleotide supplementation should provide a benefit. To test this hypothesis, we treated control and PRDX1-depleted U2-OS cells with etoposide in the presence or absence of nucleotide supplementation and followed γH2AX dynamics at 0-, 2-, and 4-h postrelease. As expected, in the control population, we observed that etoposide treatment, in the absence of nucleotides, increased γH2AX over time. Nucleotide supplementation further augmented

© 2023 The Authors

the number of γH2AX-positive cells within the population, likely provoking nucleotide imbalance and replication stress. Conversely, U2-OS PRDX1-depleted cells cultured in presence of nucleotides showed a greater capability of DNA damage recovery than in the absence of nucleotides. Indeed, in PRDX1-depleted cells, the signal of γH2AX in the presence of nucleotides showed a significant decrease between 2 and 4 h of release (Fig 6K).

Overall, our data identify PRDX1 as an important DNA damage surveillance factor, which is crucial for cellular proliferation. We report that PRDX1 contributes to the clearance of ROS generated in the nucleus following etoposide treatment. ROS clearance requires GSH synthesis and GSH-GSSG balancing. We observed that in the absence of PRDX1, cells accumulate GSSG, which indicates that they have a reduced ROS scavenging capability. We found that aspartate levels are compromised in PRDX1-deficient cells, which in turn reduces the ability of these cells to perform *de novo* nucleotide synthesis, finally causing replication stress and DNA damage (Fig 7). In line with this observation, aspartate supplementation helps PRDX1-depleted cells to reduce replication stress and DNA damage, while nucleotide supplementation promotes better recovery in the presence of etoposide.

Discussion

In this study, we took a variety of -omics approaches to evaluate the crosstalk between metabolism and the DNA damage response. By integrating metabolism-focused CRISPR-Cas9 genetic screens, chromatin proteomics, and targeted metabolomics in basal conditions and after the generation of DSBs by etoposide, we identified metabolic pathways that play a crucial role in maintaining genome integrity. First, several proteins from the ETC were synthetic viable with etoposide treatment and were found to be recruited to chromatin after DSB induction. Nuclear ROS were generated during etoposide treatment and persisted up to 24 h after treatment. This suggests an important role of the ROS signaling and scavenging processes in maintaining genomic integrity following the generation of DNA DSBs with etoposide. Second, etoposide treatment induced profound perturbations in the cellular metabolome that remained altered up to 24 h after drug release, in line with ROS nuclear levels. The main perturbed metabolites were nucleoside-related, indicating that during the DNA damage response, cells synthesize nucleotides to repair the DNA lesions.

The robustness of our data is confirmed by its intersection with the published literature and the identification of well-known DNA damage response factors in our genetic and proteomic datasets. Indeed, among the proteins differentially recruited to chromatin after etoposide treatment and release, RAD18, which signals DNA damage and functions as an adaptor to recruit homologous recombination proteins (Huang *et al*, 2009), was enriched on chromatin following etoposide treatment and returned to basal levels after 24-h release. BRCA1 and BARD1, which form a heterodimer involved in the DNA damage response to DSBs, followed a comparable recruitment pattern as RAD18 (Dai *et al*, 2021). Additionally, the chromatin regulator DnaJ homolog subfamily C member 2 (DNAJC2) binds monoubiquitylated histone H2A (Gracheva *et al*, 2016), an epigenetic mark that functions in DNA damage signaling and recruitment of DNA repair proteins early in the DNA damage response, which explains its accumulation on chromatin immediately after etoposide treatment. On the contrary, PCNA, involved in DNA synthesis, and CDC26, which is required to elicit anaphase (Jin *et al*, 2008), were depleted from chromatin at 24-h postetoposide release, potentially due to a reduction in cellular proliferation and partial cell cycle arrest following DSB induction (Fig EV2H). NUCKS1, involved in homologous recombination, is lost from chromatin upon etoposide treatment and at 24 h of release. This transcription factor binds chromatin in a cell cycle-dependent manner and its levels increase in late G1, thus explaining this enrichment on chromatin (Parplys *et al*, 2015; Hume *et al*, 2021). Similarly, SMC4, a component of the condensin complex facilitating the sister chromatid condensation and mitosis, which is also involved in DNA repair (Wang & Wu, 2021), is depleted from chromatin immediately after etoposide treatment, but unlike NUCKS1, it is fully restored to basal levels 24 h after release.

Data analysis has revealed that many metabolic enzymes and pathways are involved in the generation or the repair of DNA damage, and further investigation is needed to understand how each of them is specifically implicated in the convoluted cellular response to DNA damage. To further dissect which metabolic pathways are involved in DNA repair, the presented datasets could be complemented with additional approaches. First, chromatome proteomics is limited to the identification of proteins that directly bind DNA upon DNA damage. It does not account for secondary interactors which could play a crucial role in the DNA damage response. Performing mass-spectrometry on nuclear extracts including the soluble nuclear fraction could be a complementary approach to that end which would allow for the discovery of additional metabolic enzymes involved in DNA repair. Similarly, while the panel of metabolites measured in our targeted metabolomics approach is broad and comprises different kinds of metabolites, performing untargeted metabolomics, although more challenging, would allow for the identification of other metabolites perturbed by etoposide treatment (Schrimpe-Rutledge *et al*, 2016).

Among the metabolic pathways that we identified as linked with the DNA damage response, mitochondrial respiration plays an important role, which could be harnessed to design better anticancer regimens. In the genetic screen, genes of the TCA and ETC that are essential for cellular energy production were synthetically viable with DNA damage, while the HIF complex, which can induce downregulation of mitochondrial respiration, was synthetic lethal. Additionally, in the chromatome dataset, ETC proteins were enriched on chromatin after etoposide treatment, suggesting that this unexpected subcellular localization may participate in the etoposide-mediated nuclear ROS increase. Rapidly proliferating cancer cells have an increased demand in biomass synthesis to support cell growth and often face hypoxia due to the lack of oxygenation in tumors (Paredes *et al*, 2021). Therefore, tumors need to undergo metabolic adaptation and change their nutrient utilization during the different stages of malignancy, which can deregulate TCA and ETC processes. Our data suggest that cells with lower ETC activity and heightened glycolytic signaling would be more resistant to the induction of DSBs by etoposide treatment. This hypothesis is corroborated by a study showing that the cellular metabolism of colorectal cancer cells is activated following treatment with replication stress-inducing drugs, to provide biomolecules necessary for DNA repair and survival (Marx *et al*, 2022). This study also discovered

 © 2023 The Authors

that p53-proficient cells upregulate their metabolism more than p53-deficient cells, and therefore rely more heavily on glucose for their survival. Analyzing the metabolic status of tumors could thus be important to predict patient responses to DNA-damaging agents and to design the most appropriate anticancer therapies.

Similarly, cancer cells usually present higher levels of ROS in basal conditions due to their increased metabolic activity, but they adapt their antioxidant capacities to maintain redox homeostasis (Kim *et al*, 2019). Currently, anticancer therapies that manipulate ROS levels are being developed, either by inducing more ROS or by inhibiting antioxidant processes, in order to overwhelm cancer cells and disrupt the redox balance, leading to cell death. Prooxidants and antioxidant inhibitors are currently studied in clinical trials, as well as ROS-based repurposed drugs (Wang *et al*, 2021). Our study demonstrates that dual treatment with etoposide and drugs increasing ROS levels could be a potent strategy to kill cancer cells faster and overcome chemoresistance.

Another metabolic pathway that we found tightly connected with DNA damage and repair is nucleotide metabolism. Indeed, deletion of genes involved in this essential cellular process led to the accumulation of DNA damage in our genetic screens, probably because of nucleotide pool imbalance, which would result in nucleotide misincorporation, replication stress, and accumulation of DNA damage (Bester *et al*, 2011; Buckland *et al*, 2014; Diehl *et al*, 2022). In addition, nucleoside-containing metabolites were also drastically perturbed after etoposide treatment in our metabolomics dataset, suggesting that nucleotides were acutely depleted following DSB generation, which then triggered *de novo* nucleotide synthesis to replenish nucleotide pools. Therefore, we hypothesize that combining etoposide with inhibitors of nucleotide synthesis processes could potentiate the effect of etoposide, by preventing the repair of DNA damage. However, nucleotide synthesis is a key cellular process, and the development of inhibitors is limited by toxicity, which could be alleviated by identifying and targeting regulatory mechanisms specific to tumor cells or to tissue types. Few organ-specific metabolites in tumors have been discovered, and the development of compounds targeting enzymes producing these metabolites holds great promise for patient treatment (Feng *et al*, 2020; Ma *et al*, 2021).

Intersecting our datasets led to the identification of the peroxiredoxin PRDX1 as a key factor in the DNA damage surveillance processes. This enzyme has a dual function, as a peroxidase with a ROS scavenging function, and as a molecular chaperone that can modulate transcription factor activities upon oxidation (Morinaka *et al*, 2011; Mu *et al*, 2002). It has been shown to have a controversial role in cancer metabolism. On one hand, it is overexpressed in some malignant tumors, but on the other hand, PRDX1-deficient mice are prone to develop cancers (Neumann *et al*, 2003). PRDX1 regulates several transcription factors involved in tumorigenesis. In one example, it interacts with c-Myc and suppresses the regulation of some target genes, thus limiting tumor growth (Mu *et al*, 2002). In another example, cytoplasmic PRDX1 suppresses NF-κB activation by preventing peroxide accumulation, while nuclear PRDX1 enhances NF-κB activity (Hansen *et al*, 2007). Moreover, it has been shown that targeting PRDX1 sensitizes breast cancer cells to pro-oxidative agents (Bajor *et al*, 2018). The relationship between PRDX1 and cancer, therefore, appears to depend on many factors including tissue specificity. Hence, a better understanding of the functions of this protein is crucial.

In our study, we demonstrate that the role of PRDX1 in response to DNA damage is not restricted to telomeres, thus going beyond a previously published study (Ahmed & Lingner, 2018). We identify two main roles of PRDX1 in the DNA damage response. First, it scavenges nuclear ROS generated by etoposide treatment after translocating to the nucleus. Second, PRDX1 depletion induces perturbations in aspartate-related metabolites which results in impacting GSH-GSSG balance and nucleotide pools. PRDX1 loss severely affects cellular proliferation and leads to DNA damage and replication stress even in the absence of DNA damage inducers, which could be due to both accumulation of ROS and alteration of the nucleotide pool. Interestingly, while aspartate levels were reduced in PRDX1-deficient cells, several nucleotide monophosphate levels were upregulated. We hypothesize that nucleotide salvage pathways are compensating for the downregulated *de novo* nucleotide synthesis due to reduced aspartate. Moreover, while nucleotide monophosphates are elevated in PRDX1-deficient cells, nucleotide triphosphates do not follow this trend, perhaps suggesting a defect in the enzymes responsible for this conversion. It has been demonstrated that aspartate metabolism is perturbed in cancer cells to support proliferation. For example, arginosuccinate synthase (ASS1), which converts nitrogen from ammonia and aspartate to urea, is silenced in several cancers, thus leading to an accumulation of cytosolic aspartate and fostering *de novo* pyrimidine synthesis to support cancerous proliferation (Rabinovich *et al*, 2015). The ETC plays an essential role in aspartate synthesis (Birsoy *et al*, 2015; Sullivan *et al*, 2015). When the ETC is inhibited, for example in hypoxia, a common in tumors, aspartate synthesis becomes limiting and cancer cells need to import extracellular aspartate to maintain cellular growth (Garcia-Bermudez *et al*, 2018). In addition, endogenous aspartate is produced in the mitochondria but needs to be exported to the cytoplasm, where it can be used for nucleotide and amino acid synthesis. In low-glutamine conditions especially, sustaining cytosolic aspartate concentration is critical for cell survival (Alkan *et al*, 2018). Therefore, the controversial role of PRDX1 in cancer metabolism might also be linked with its regulation of aspartate metabolism. In our study, aspartate supplementation of PRDX1-deficient cells did not lead to a full rescue of the phenotypes associated with PRDX1 loss. We observed a decrease in the generation of DNA DSBs in basal conditions after treating HT1080 PRDX1-deficient cells with aspartate for 3 days, but we did not observe a rescue on the growth defect. Uptake capacities of exogenous aspartate are cell-type dependent because it requires the presence of specific transporters for cellular import, such as SLC1A3 (Garcia-Bermudez *et al*, 2018), but in most cells, endogenous aspartate is preferentially used (Sullivan *et al*, 2018). Analysis of publicly available transcriptomics data (Ghandi *et al*, 2019) indicated that the HT1080 cells used for this experiment only mildly express the transporter SLC1A3 as compared to cell lines with detectable aspartate import activity described by Garcia-Bermudez *et al*. Therefore, exogenous aspartate supplementation might not be sufficient to restore normal levels of aspartate in U2-OS-depleted and HT1080 PRDX1-deficient cells. Additionally, the impact of PRDX1 loss on ROS scavenging might require extra-aspartate for the synthesis of glutamate and GSH, thus being the principal cause of defects in cellular proliferation.

Our study sheds light on the interplay between cellular metabolism and the DNA damage response. This is particularly

© 2023 The Authors

relevant in cancer, which can be considered both a metabolic and a genetic disease, thus better understanding of this crosstalk better could help design more efficient and targeted therapies. While the role of PRDX1 in the DNA damage and repair processes as well as in tumorigenesis has been reported, future work will be needed to elucidate in which conditions it functions as a tumor suppressor or, on the contrary, facilitates tumor development.

Materials and Methods

Reagents and Tools table

Reagent/Resource	Reference or Source	Identifier or Catalog Number
**Experimental Models**		
U2-OS (H. Sapiens)	ATCC	
U2-OS sgPRDX1 (H. Sapiens)	This study	
U2-OS shPRDX1 (H. Sapiens)	This study	
HT1080 (H. Sapiens)	Joachim Lingner (Aeby *et al*, 2016)	
HT1080 PRDX1$^{-/-}$ (H. Sapiens)	Joachim Lingner (Aeby *et al*, 2016)	
HEK293T-xLenti	Oxgene	
**Recombinant DNA**		
Human metabolic knockout pooled CRISPR library	Addgene	Cat # 110066
psPAX2	Addgene	Cat # 12260
VSV.G	Addgene	Cat # 14888
plentiCRISPR v3	Horizon	Cross *et al* (2016)
pLKO.2	Sebastian Nijman	
pKAM-GFP	Addgene	Cat # 101865
pLL3.7m-mTurquoise2-SLBP(18-126)-IRES-H1-mMaroon1	Addgene	Cat # 83842
pLL3.7m-Clover-Geminin(1-110)-IRES-mKO2-Cdt(30-120)	Addgene	Cat # 83841
**Antibodies**		
COX4 polyclonal antibody, rabbit (1:200 IF)	ThermoFisher	PA5-19471
gH2AX monoclonal antibody, clone JBW301, mouse (1:1,000 IF)	Merck	05-636-I
Phospho-ATR (Ser428) polyclonal antibody, rabbit (1:100 IF)	Cell signaling	#2853
PCNA monoclonal antibody, clone PC10, mouse (1:1,000 IF)	Santa-Cruz	sc-56
PRDX1 recombinant monoclonal antibody, clone [EPR5434], rabbit (1:200 IF; 1:1,000 WB)	abcam	ab109506
Phospho-RPA32 (Ser4, Ser8) polyclonal antibody, rabbit (1:1,000 IF)	Bethyl	A300-245A
RPA32/RPA2 monoclonal antibody, clone 9H8, mouse (1:500 IF)	abcam	ab2175
RPA70 recombinant monoclonal antibody, clone [EPR3472], rabbit (1:500 IF)	abcam	ab79398
TRF1 polyclonal antibody, rabbit (1:500 IF)	abcam	ab1423
TRF2 recombinant monoclonal antibody, clone [EPR3517(2)], rabbit (1:300 IF)	abcam	ab108997
Alpha-tubulin monoclonal antibody, clone DM1A, mouse (1:10,000 WB)	Cell signaling	#3873
Vinculin monoclonal antibody, clone E1E9V, rabbit (1:1,000 WB)	Cell signaling	#13901
H3 polyclonal antibody, rabbit (1:10,000 WB)	abcam	ab1791
FDX1 polyclonal antibody, rabbit (1:500 WB)	ThermoFisher	PA5-59653
Anti-mouse and anti-rabbit HRP conjugated goat secondary antibodies (1:5,000 WB)	Jackson Immunochemicals	115-035-003/ 111-035-003
Anti-mouse AF568 goat secondary antibody (1:2,000 IF)	Molecular Probes	A11004
Anti-BrdU, rat	Bio-Rad	MCA6144
Anti-BrdU, mouse	Becton Dickinson	MAB7225

© 2023 The Authors

Reagents and Tools table (continued)

Reagent/Resource	Reference or Source	Identifier or Catalog Number
Anti-mouse AF555 goat secondary antibody	ThermoFisher	A21424
Anti-COX4 polyclonal antibody, rabbit (1:1,000 IF)	abcam	ab16056
Anti-rabbit AF555 goat secondary antibody	ThermoFisher	A32732
**Oligonucleotides and sequence-based reagents**		
shPRDX1-1	TRCN database (http://www.broadinstitute.org/rnai/public/gene/search)	5′-GATGAGACTTTGAGACTAGTT-3′
shPRDX1-2	TRCN database (http://www.broadinstitute.org/rnai/public/gene/search)	5′-CCAGATGGTCAGTTTAAAGAT-3′
shControl	TRCN database (http://www.broadinstitute.org/rnai/public/gene/search)	5′-CTTACGCTAGTACTTCGA-3′
sgPRDX1	Aeby *et al* (2016)	5′-GCCACAGCTGTTATGCCAGA-3′
**Chemicals, enzymes and other reagents**		
Etoposide	Sigma-Aldrich	E1383-25MG
Neocarzinostatin from Streptomyces carzinostaticus	Sigma-Aldrich	N9162-100UG
Phenformin	MedChem Express	HY-16397A
TTFA	abcam	ab223880
Aspartic acid	Sigma-Aldrich/ Merck	A1330000/ A7219
Carboplatin	MedChem Express	HY-17393
AgeI-HF	NEB	R3552S
EcoRI-HF	NEB	R3101S
BsmBI	NEB	R0580L
Lipofectamine 2000 transfection reagent	Thermo Fisher Scientific	11668019
Puromycin dihydrochloride	Gibco	A1113803
S.p. Cas9 nuclease V3	Integrated DNA Technology	#1081059
HiScribe	NEB	E2050S
SE Cell-Line Solution	Lonza	V4XC-1032
Ex Taq DNA polymerase	Takara	RR001A
Agencourt AMPure XP DNA beads	Beckman Coulter	A63880
Benzonase nuclease	VWR	E1014
cOmplete protease inhibitor cocktail	Roche	11836170001
Paramagnetic carboxylate modified particles (SpeedBeads)	GE Healthcare	45152105050250 and 65152105050250
Trifluoroacetic acid Uvasol	Merck	302031
ReproSil-Pur 120 C18-AQ, 3 μm	Dr. Maisch	r13.a.q.
NuPAGE LDS Sample Buffer	Invitrogen	NP0007
Amersham™ Protran nitrocellulose membrane	Cytiva	GE10600002
Hoechst 33342	Life Technologies	33342
CldU	Sigma-Aldrich	C6891
IdU	Sigma-Aldrich	I7125
Ascorbic acid	Merck	A4544
CellROX Green	Life Technologies	M22426
Mitotracker Deep Red FM	Life Technologies	P36935
ProLong™ Gold Antifade Mountant with DAPI	ThermoFisher	P36935
Nucleotides	ThermoFisher	R0441/ R0451/ R0461/ R0471

© 2023 The Authors

Reagents and Tools table (continued)

Reagent/Resource	Reference or Source	Identifier or Catalog Number
**Software**		
MAGeCK	Li *et al* (2014)	
MAGeCKFlute	Wang *et al* (2019)	
CellProfiler version 4.1.3	Stirling *et al* (2021)	
Xcalibur version 4.3.73.11	Thermo Scientific	
Tune version 3.4.3072.18	Thermo Scientific	
sva R package (version 3.12.0)	Leek *et al* (2012)	
DEP R package	Zhang *et al* (2018)	
proDA R package	(Ahlmann-Eltze (2022))	
imp4p R package	preprint: Gianetto *et al* (2020)	
SubCellularBarCode R package	Arslan (2021)	
tidyverse collection of packages	Wickham *et al* (2019)	
MassHunter 10.0 software	Agilent Technologies	
decoupleR R package	Badia-i-Mompel *et al* (2022)	
FlowJo version 10	Becton Dickinson	
ImageJ Fiji software	Schindelin *et al* (2012)	
Harmony software	Perkin Elmer	
clusterProfiler R package	Wu *et al* (2021)	
**Other**		
Illumina HiSeq 3000/4000	Illumina	
Orbitrap Fusion Lumos mass spectrometer	Thermo Fisher Scientific	
Dionex Ultimate 3000RSLC nanosystem	Thermo Fisher Scientific	
1290 Infinity II UHPLC system	Agilent Technologies	
6470 triple quadrupole mass spectrometer	Agilent Technologies	
LSR-Fortessa X-20	BD Bioscience	
FACS Melody	BD Bioscience	
Opera High Content Screening System	Perkin Elmer	
Operetta High Content Screening System	Perkin Elmer	
dmi6000b microscope	Leica	
IXplore SpinSR spinning disk confocal microscope	Olympus Life Science	
LSM700 confocal microscope	Zeiss	
A1R Ultra-Fast Spectral Scanning Confocal Microscope	Nikon	
4D-Nucleofector System X-Unit	Lonza	
Curix 60 tabletop processor	AGFA	
Bioruptor Pico	Diagenode	
BioAnalyzer 2100	Agilent Technologies	
Magnetic rack (DynaMag-2 Magnet)	Thermo Fisher Scientific	
C18 solid phase extraction spin column	Pierce Biotechnology	
Trap column Pepmap 100 5 μm, 5 × 0.3 mm	Thermo Fisher Scientific	
ZORBAX RRHD Extend-C18, 2.1 × 150 mm, 1.8 μm analytical column	Agilent Technologies	
384-well black plates (CellCarrier-Ultra)	Perkin Elmer	
QIAmp Blood Midi kit	QIAgen	
QIAgen Miniprep kit	QIAgen	
BCA protein assay kit	Applichem CmBH	
Pacific Blue™ Annexin V Apoptosis Detection Kit with PI	BioLegend	

© 2023 The Authors

Methods and Protocols

Plasmids and reagents

The human metabolic knockout pooled CRISPR library was a gift from David Sabatini (Addgene # 110066). The library consists of 29,790 sgRNAs targeting 2,981 metabolism-related genes, with ~10 sgRNA/gene, as well as 500 intergenic control sgRNAs in a Cas9-expressing lentiviral vector. For lentivirus production, the psPAX2 (a gift from Didier Trono; Addgene plasmid # 12260) and VSV.G (a gift from Tannishtha Reya; Addgene plasmid # 14888) packaging plasmids were used. plentiCRISPR v3 was bought from Horizon (Cross *et al*, 2016) and pLKO.2 was a kind gift from Sebastian Nijman (Ludwig Cancer Research, Oxford, UK). For the competition assay, the pKAM-GFP plasmid (a gift from Archibald Perkins, Addgene plasmid #101865) was used to tag the cells with GFP. The plasmids used for the FUCCI system, pLL3.7 m-mTurquoise2-SLBP (18-126)-IRES-H1-mMaroon1 and pLL3.7 m-Clover-Geminin(1-110)-IRES-mKO2-Cdt(30-120) were a gift from Michael Lin (Addgene plasmids #83842 and #83841, respectively).

Etoposide, NCS from Streptomyces carzinostaticus ≥ 90%, and aspartate were obtained from Sigma-Aldrich. Phenformin and Carboplatin were obtained from MedChem Express. TTFA was obtained from Abcam.

Human cell culture

All cells were grown at 37°C at 5% CO_2 and 3% O_2. Human bone osteosarcoma epithelial U2- OS cells were purchased from the ATCC cell repository. Human fibrosarcoma epithelial HT1080 cells, both WT and clonal deficient for PRDX1 ($PRDX1^{-/-}$), were a kind gift from Joachim Lingner (Swiss Institute for Experimental Cancer Research (ISREC), Ecole Polytechnique Fédérale de Lausanne [EPFL]; Aeby *et al*, 2016). Ablation of protein expression was confirmed by immunoblotting for PRDX1. All cells were cultured in Dulbecco's Modified Eagle Medium (DMEM, Gibco), supplemented with 10% Foetal Bovine Serum (FBS, Gibco) and 1% penicillin/streptomycin (Sigma-Aldrich). Cells were monthly tested for mycoplasma contamination.

Generation of U2-OS PRDX1-depleted cells

For shRNA-mediated depletion of PRDX1, two shRNAs (shPRDX1-1 and shPRDX1-2) targeting the coding region of the gene (5′-GATGA GACTTTGAGACTAGTT-3′ and 5′- CCAGATGGTCAGTTTAAAGAT-3′) and one non-targeting shRNA (shControl, 5′- CTTACGCTAGTAC TTCGA-3′) were used. The shRNA sequences were obtained from the TRCN database (http://www.broadinstitute.org/rnai/public/gene/search) and cloned into the lentiviral vector pLKO.2 using AgeI and EcoRI restriction sites. For sgRNA-mediated depletion of PRDX1, a sgRNA targeting PRDX1 (5′-GCCACAGCTGTTATGCC AGA-3′) was cloned into the lentiviral vector plentiCRISPRv3 using BsmB1 restriction sites. Insertion of shRNA and sgRNA sequences was verified by Sanger sequencing.

Lentiviral particles were produced by transfection of the shRNA-containing pLKO.2 or the sgRNA-containing plentiCRISPRv3 constructs along with the packaging plasmids psPAX2 and VSV.G into HEK-xLenti™ cells (Oxgene) cells using Lipofectamine 2000. Two and three days after transfection, the virus-containing supernatant was harvested and centrifuged to remove packaging cells from the supernatant. U2-OS cells were infected by spinfection with the virus-containing supernatant in the presence of polybrene (final concentration 8 μg/ml). Infected cells were selected using puromycin (1.5 μg/ml; Gibco) for 72 h.

To increase knock-out efficiency, the sgPRDX1 U2-OS population was additionally nucleofected with the purified S.p. Cas9 nuclease V3 (#1081059, Integrated DNA Technology) together with *in vitro* transcribed sgRNA targeting PRDX1 (5′-GCCACAGCTGTTATGCC AGA-3′). T7 *in vitro* transcription was performed using HiScribe (NEB E2050S), using PCR-generated DNA as a template. The 4D-Nucleofector System X-Unit (Lonza) was used for nucleofection, with the SE Cell-Line Solution (V4XC-1032, Lonza) and the CM-104 program, in Nucleocuvette™ strips (Lonza).

A decrease in protein expression in the whole population was confirmed by immunoblotting for PRDX1.

CRISPR screens

Pooled CRISPR screen

Library amplification: The metabolic CRISPR pooled library was amplified following the distributor's instructions (Addgene), with a coverage of around 200×.

Virus production: HEK-xLenti™ cells (Oxgene) were seeded in 12xT225 flasks 10-cm dishes and transfected 24 h later, with the metabolic CRISPR pooled library, pVSVG, and psPAX2 packaging plasmids, using polyethyleneimine (PEI) in OptiMeM (Gibco). The medium was changed 10 h later. Twenty four and 48 h later, the supernatant containing virus was harvested and centrifuged at 600 *g* for 5 min to remove cell debris. The two batches were pooled together and the virus was concentrated 20× using PEG-8000 and stored at −80°C.

Cell infection and harvest: U2-OS cells were spinfected for 3 h at 2,000 rpm and 37°C in 12-well plates with the lentiviral metabolic library at a multiplicity of infection (MOI) of 0.3–0.5 in presence of polybrene (final concentration 8 μg/ml). Immediately after spinfection, cells were collected and seeded in 245-mm-square dishes with fresh medium. Puromycin-containing medium (1.5 μg/ml) was added the next day to select transductants. At 7 days of post-transduction, cells were re-seeded, and at 9 days of post-transduction, they were either treated with 1 μM etoposide for 3 h or left untreated. Treated cells were washed with PBS and released in drug-free media, and after 24 h of release, both untreated and treated cells were harvested. Part of the harvested cells was re-seeded to be harvested at a later timepoint, maintaining 1,000× coverage, while the rest of the cells were fixed with ice-cold 90% methanol in PBS at a density of 8 million cells/ml, and stored in methanol at −20°C. At 14 days after transduction, both untreated and treated cells were harvested, methanol-fixed, and stored at −20°C.

Immunofluorescence staining and FACS: 300 million cells harvested at 24 h of release after etoposide treatment, fixed in methanol and stored at −20°C, were stained for γH2AX and with propidium iodide (PI) as described in the flow cytometry section, except that γH2AX antibody was diluted 1:300, using 100 μl/10 million cells, and AF488-anti-mouse antibody was diluted 1:250, using 100 μl/10 million cells. After PI staining in batches, cells were filtered and sorted on a SONY SH800 sorter, for the top 5% and the lowest 10% γH2AX populations. Sorted cells of different batches were pooled and stored as cell pellets at −80 until DNA extraction. For consistency, unsorted samples stored in methanol were also

© 2023 The Authors

washed with PBS, FACS buffer, and PBS, and stored as pellets at −80 until DNA extraction.

Genomic DNA extraction and sgRNA amplification: Genomic DNA from all samples was extracted using the QIAmp DNA Blood Midi kit using a protocol from the Broad Institute, treated with RNaseA, and then ethanol precipitated to concentrate the DNA. The sgRNA library was prepared using a one-step PCR with ExTaq polymerase (Takara) and a mixture of P5 forward primers with staggers from 1 to 8 bp and barcoded P7 reverse primers. Cell cycle number was optimized for each sample to ensure that there was no overamplification and the used DNA input for each sample corresponded to a coverage of ~500×. PCR products were purified by size exclusion using magnetic AMPure XP DNA beads (Beckman Coulter) until DNA electrophoresis profiles showed clean peaks (BioAnalyzer 2100, Agilent).

NGS analysis: Barcoded samples were pooled in equal quantities after measurement of DNA concentrations by fluorometric quantification (Qubit, ThermoFisher Scientific), and sequenced on one lane of an Illumina HiSeq 3000/4000 machine using single-read sequencing. After de-multiplexing, sgRNA sequences were retrieved by trimming all sequences 5′ to the adapter sequence (5′- GACGAAACA CC-3′) and 20 nucleotides 3′ following this. MAGeCK was used for alignment, gRNA count, copy number variation (CNV) correction, and gene-level depletion scores (Li *et al*, 2014). MAGeCKFlute (Wang *et al*, 2019) was additionally used to correct for cell cycle-related effects between etoposide-treated and untreated samples. sgRNA counts were normalized to million counts, for each sequencing sample, and gene log2(fold-change) was calculated by taking the average of the log2(fold-change) for all sgRNAs targeting the same gene. The next-generation sequencing raw data from this publication have been deposited to the European Nucleotide Archive (ENA) database and assigned the identifier [ERA16463919] (https://www.ebi.ac.uk/ena/browser/view/PRJEB54700).

Arrayed CRISPR screen

Library cloning: The arrayed library was designed to target the top genes whose depletion led to increased γH2AX levels at 24 h release postetoposide treatment in the pooled metabolic CRISPR screen, excluding the transporters ABCB1 and ABCB7, which have known roles in multidrug resistance. Each gene was targeted by 4 sgRNAs: 2 that were showing the strongest phenotype in the pooled screen and 2 that had the highest score in Toronto KnockOut Library v3 (TKOv3, https://crispr.ccbr.utoronto.ca/crisprdb/public/library/TKOv3/). Additionally, 4 intergenic controls were selected from the pooled library and 3 sgRNAs targeting the DNA repair genes LIG4 and XRCC4 were selected from the TKOv3 library, as positive controls.

sgRNAs were cloned in plentiCRISPRv3 using the BsmBI restriction sites, in a 96-well plate format. To amplify the plasmids, Stbl3 bacteria were transformed with the ligation reaction in 96-well deep well plates until OD is approximately 0.1. Then bacteria expressing sgRNAs targeting the same genes were pooled and plasmid DNA extraction was performed using the QIAgen Miniprep kit following the manufacturer's instructions. Representation of sgRNAs and cross-contamination between wells was checked by NGS sequencing after one-step PCR to amplify sgRNA sequences, both P5 forward primers and P7 reverse primers being barcoded.

Virus production The virus was produced following the same protocol as for the pooled screen except that HEK-xLenti™ cells (Oxgene) were seeded in six-well plates and each well was transfected with the mixture of sgRNA-containing plentiCRISPRv3 constructs targeting the same gene using Lipofectamin2000 (ThermoFisher). Virus-containing supernatant was aliquoted and frozen at −80°C.

Screen setup: U2-OS cells were spinfected for 2 h at 2,000 rpm and 32°C in 96-well plates with the arrayed library at a high MOI in presence of polybrene (final concentration 8 μg/mL). Puromycin-containing medium (1.5 μg/ml) was added the next day to select transductants. At 6 days of post-transduction, selected cells were seeded in 384-well plates, with duplicated wells for each targeted gene. One day later, they were either treated with 1 μM etoposide for 3 h or 60 ng/mL NCS for 1 h or left untreated. Treated plates were either fixed with 2% PFA in PBS immediately after treatment or after 20 h of release in drug-free media. Untreated plates were fixed at the same time as 20 h release plates. γH2AX and DAPI staining was performed as described in the immunofluorescence microscopy section. Images were acquired on an Opera High Content Screening System (Perkin Elmer) using x40 magnification. Quantification of the number of foci per cell was done using CellProfiler software version 4.1.3 (Stirling *et al*, 2021). To account for interexperiment variability, the number of foci in each condition was normalized to the number of foci in the untreated intergenic condition for each biological replicate.

Chromatome proteomics

Sample preparation: 5 million U2-OS cells were incubated in CHAPS buffer for 20 min on ice (0.5% CHAPS in PBS 1×) and centrifuged for 5 min at 720 *g* at 4°C. The supernatant was saved as "Cytoplasmic fraction" and the nuclei were resuspended in Cytoplasmic Lysis Buffer (0.1% IGEPAL, 10 mM Tris–HCl ph 7, 150 mM NaCl). The dirty nuclei were placed on Sucrose Buffer (10 mM Tris–HCl ph 7, 150 mM NaCl, 25% Sucrose) and centrifuged for 15 min at 10,000 *g* and 4°C. The nuclei were washed three times by resuspending with Nuclei Washing Buffer (0.1% IGEPAL and 1 mM EDTA in PBS 1×) and spinning for 5 min at 1,200 *g* and 4°C. The clean nuclei were resuspended in Nuclei Resuspension Buffer (20 mM Tris–HCl ph 8, 75 mM NaCl, 1 mM EDTA, 50% Sucrose) and lysed by adding Nuclei Lysis Buffer (0.1% IGEPAL, 20 mM HEPES pH 7.5, 300 mM NaCl, 0.2 mM EDTA), vortexing and incubating for 2 min on ice. The nuclei extract was centrifuged for 2 min at 16,000 *g* and 4°C and the chromatin pellet resuspended in Benzonase Digestion Buffer (0.1% IGEPAL, 15 mM HEPES pH 7.5, 5 μg/μl TPCK). The chromatin was sonicated on a Bioruptor Pico for 15 cycles 30 s ON and 30 s OFF in 1.5-ml Diagenode tubes, the DNA was digested with 2.5 U Benzonase (VWR) for 30 min at RT and the resulting extract was saved as "Chromatome fraction." All buffers contained "Complete" proteinase inhibitor (Roche) according to the manufacturer's directions.

Liquid chromatography coupled to tandem mass spectrometry (LC–MS/MS): The protein concentrations from chromatin-enriched samples were determined using the BCA protein assay kit (Applichem CmBH, Darmstadt, Germany), and 10 μg per sample was processed using an adapted Single-Pot solid-phase-enhanced sample preparation (SP3) methodology (Hughes *et al*, 2014). Briefly, equal volumes (125 μL containing 6,250 μg) of two different kinds of paramagnetic carboxylate modified particles (SpeedBeads

© 2023 The Authors

45152105050250 and 65152105050250; GE Healthcare) were mixed, washed three times with 250 µl water and reconstituted to a final concentration of 50 µg/µl with LC–MS grade water (LiChrosolv; MERCK KgaA). Samples were filled up to 100 µl with stock solutions to reach a final concentration of 2% SDS, 100 mM HEPES, pH 8.0, and proteins were reduced by incubation with a final concentration of 10 mM DTT for 1 h at 56°C. After cooling down to room temperature, reduced cysteines were alkylated with iodoacetamide at a final concentration of 55 mM for 30 min in the dark. For tryptic digestion, 400 µg of mixed beads was added to reduced and alkylated samples, vortexed gently, and incubated for 5 min at room temperature. The formed particles–protein complexes were precipitated by the addition of acetonitrile to a final concentration of 70% [V/V] and mixed briefly via pipetting before incubating for 18 min at room temperature. Particles were then immobilized using a magnetic rack (DynaMag-2 Magnet; Thermo Fisher Scientific) and the supernatant was discarded. SDS was removed by washing two times with 200 µl 70% ethanol and one time with 180 µl 100% acetonitrile. After the removal of the organic solvent, particles were resuspended in 100 µl of 50 mM NH4HCO3, and samples were digested by incubating with 2 µg of Trypsin overnight at 37°C. Samples were acidified to a final concentration of 1% Trifluoroacetic acid (Uvasol; MERCK KgaA) prior to immobilizing the beads on the magnetic rack. Peptides were desalted using C18 solid phase extraction spin columns (Pierce Biotechnology, Rockford, IL). Finally, eluates were dried in a vacuum concentrator and reconstituted in 10 µl of 0.1% TFA.

Mass spectrometry was performed on an Orbitrap Fusion Lumos mass spectrometer (ThermoFisher Scientific, San Jose, CA) coupled to a Dionex Ultimate 3000RSLC nanosystem (ThermoFisher Scientific, San Jose, CA) via nanoflex source interface. Tryptic peptides were loaded onto a trap column (Pepmap 100 5 µm, 5 × 0.3 mm, ThermoFisher Scientific, San Jose, CA) at a flow rate of 10 µL/min using 0.1% TFA as loading buffer. After loading, the trap column was switched in-line with a 50 cm, 75 µm inner diameter analytical column (packed in-house with ReproSil-Pur 120 C18-AQ, 3 µm, Dr. Maisch, Ammerbuch-Entringen, Germany). Mobile-phase A consisted of 0.4% formic acid in water and mobile-phase B of 0.4% formic acid in a mix of 90% acetonitrile and 10% water. The flow rate was set to 230 nl/min and a 90 min gradient was used (4–24% solvent B within 82 min, 24–36% solvent B within 8 min, and, 36–100% solvent B within 1 min, 100% solvent B for 6 min before bringing back solvent B at 4% within 1 min and equilibrating for 18 min). Analysis was performed in data-independent acquisition (DIA) mode using variable DIA windows. Full MS scans were acquired with a mass range of 375–1,250 *m/z* in the orbitrap at a resolution of 120,000 (at 200 *m/z*). The automatic gain control (AGC) was set to a target of 4 × 105, and a maximum injection time of 54 ms was applied, scanning data in profile mode. A single lock mass at *m/z* 445.120024 (Olsen *et al*, 2005) was employed. MS1 scans were followed by 41 × MS2 scans with variable isolation windows (variable DIA windows). The MS2 scans were acquired in the orbitrap at a resolution of 30,000 (at 200 *m/z*), with an AGC set to target 2 × 105, for a maximum injection time of 54 ms. Fragmentation was achieved with higher energy collision-induced dissociation (HCD) at a fixed normalized collision energy (NCE) of 35%. Xcalibur version 4.3.73.11 and Tune 3.4.3072.18 were used to operate the instrument. The mass spectrometry proteomics data have been deposited to the ProteomXchange Consortium via the PRIDE partner repository (Perez-Riverol *et al*, 2022) with the dataset identifier [PXD035532] (http://www.ebi.ac.uk/pride/archive/projects/PXD035532). Replicates 4 and 5 were removed from the acquisition as their chromatograms revealed the samples were compromised.

Data processing: Chromatin data were batched normalized using the ComBat algorithm from the sva R package (version 3.12.0, Leek *et al*, 2012) and normalized using the normalize_vsn and median_normalisation functions from the DEP (Zhang *et al*, 2018) and proDA (Ahlmann-Eltze, 2022) packages, respectively. The rest of the pipeline was followed according to the DEP package, with the inclusion of impute.mi function for protein-imputation from the imp4p package (preprint: Gianetto *et al*, 2020). Known subcellular localizations for proteins were obtained from the SubCellularBarCode R package (Arslan, 2021), and the normalization of proteins to their expected whole-cell extract (WCE) levels for untreated U2-OS cells was performed through the ProteomicRuler in Perseus and the U2-OS WCE were obtained from the CCLE proteomics dataset (Tyanova *et al*, 2016). Analysis was facilitated by the tidyverse (Wickham *et al*, 2019) collection of packages. Differential PRDX1-expression essentialities were conducted by comparing the Achilles gene essentialities between high and low PRDX1-expressing cell lines (preprint: Dempster *et al*, 2019; Ghandi *et al*, 2019).

Metabolomics

Sample preparation: U2-OS cells were seeded in six-well plates. Etoposide treatment (1 µM for 3 h) was performed at different times to be able to terminate the experiment and extract the metabolites simultaneously for all samples. At the last time point—treatment for the no-release samples—the medium was changed in all wells in order to have a growth medium of the same composition at the time of metabolite extraction. Each sample was prepared in triplicates. For metabolite collection, plates containing 0.2–0.4 million cells per well were gently washed with 75 mM ammonium carbonate buffer pH 7.4 at room temperature, transferred on ice, and metabolites were extracted with 80:20 ice-cold MeOH:H2O solution. Cells were scraped off and samples were collected in tubes, then snap-frozen in liquid nitrogen to stop all metabolic reactions. Once all wells have been collected, samples were thawed and centrifuged in a table-top centrifuge at a maximum speed at 4°C. Supernatants containing metabolites were transferred into an HPLC vial and stored at −80°C until processing by the metabolomics facility (Pro-Met, CeMM). Cleared extracts were dried under nitrogen. Samples were taken up in MS-grade water and mixed with the heavy isotope-labeled internal standard mix.

Liquid chromatography coupled to tandem mass spectrometry (LC–MS/MS): A 1290 Infinity II UHPLC system (Agilent Technologies) coupled with a 6470 triple quadrupole mass spectrometer (Agilent Technologies) was used for the LC–MS/MS analysis. The chromatographic separation for samples was carried out on a ZORBAX RRHD Extend-C18, 2.1 × 150 mm, 1.8 µm analytical column (Agilent Technologies). The column was maintained at a temperature of 40°C and 4 µl of the sample was injected per run. Mobile phase A was 3% methanol (v/v), 10 mM tributylamine, 15 mM acetic acid in water, and mobile phase B was 10 mM tributylamine, 15 mM acetic acid in methanol. The gradient elution with a flow rate of 0.25 ml/min was performed for a total time of 24 min. Afterward, back-flushing of the column using a 6port/2-position

© 2023 The Authors

divert valve was carried out for 8 min using acetonitrile, followed by 8 min of column equilibration with 100% mobile phase A. The triple quadrupole mass spectrometer was operated in negative electrospray ionization mode, spray voltage 2 kV, gas temperature 150°C, gas flow 1.3 l/min, nebulizer 45 psi, sheath gas temperature 325°C, and sheath gas flow 12 l/min. The metabolites of interest were detected using a dynamic MRM mode.

Data processing: The MassHunter 10.0 software (Agilent Technologies) was used for the data processing. Ten-point calibration curves with internal standardization were constructed for the absolute quantification of metabolites. Data were analyzed following the DEP R package for differential analysis between conditions and pathway level changes were inferred using the run_mean function from the decoupleR R package (Badia-i-Mompel *et al*, 2022). The metabolomics raw data from this publication have been deposited to the Metabolomics Workbench database (Hughes *et al*, 2014) and assigned the identifier [ST002234] (https://www.metabolomicsworkbench.org/data/DRCCMetadata.php?Mode = Study&StudyID = ST002234).

Immunoblotting

Cells were lysed in RIPA lysis buffer (New England Biolabs), sonicated and protein concentrations were measured using the Protein Assay Dye Reagent (Biorad). Samples were mixed with NuPAGE LDS Sample Buffer (Invitrogen), boiled for 5 min at 98°C and proteins were separated on SDS–PAGE gels and transferred onto Amersham™ Protran nitrocellulose membranes (0.45 µm, Cytiva). After 1 h of blocking in 5% milk in TBS-T (0.1% Tween 20 in 1× Tris-buffered saline), membranes were incubated with primary antibodies at 4°C overnight. Primary antibodies used were against PRDX1 (diluted 1:1,000, ab109506 abcam), Tubulin (diluted 1:10,000, DM1A Cell Signaling), Vinculin (diluted 1:1,000, #13901 Cell Signaling), H3 (diluted 1:10,000, ab1791 Abcam), and FDX1 (diluted 1:500, PA5-59653 Thermo Fisher Scientific). Anti-mouse and anti-rabbit HRP-conjugated goat secondary antibodies (Jackson Immunochemicals) were used at a final dilution of 1:5,000. Immunoblots were imaged using a Curix 60 (AGFA) tabletop processor. All the full size Western Blots are shown in Appendix Figure S1.

Cellular microscopy

For microscopy-based experiments, U2-OS and HT1080 cells were either seeded in 384-well plates (CellCarrier-Ultra, Perkin Elmer) or on coverslips to assess the subcellular localization of PRDX1 and COX4 by confocal microscopy. For staining of pRPA32, RPA32, RPA70, pATR, and PCNA, cells were pre-extracted with pre-extraction buffer (10 mM PIPES, 100 mM NaCl, 3 mM $MgCl_2$, 1 mM EGTA, 0.5% Triton X-100 and 300 mM Sucrose) for 10 min at 4°C, followed by Cytoskeleton Stripping Buffer B (10 mM Tris pH 7.5, 10 mM NaCl, 3 mM $MgCl_2$, 1% Tween20, 0.5% sodium deoxycholate) for additional 10 min at 4°C (O'Sullivan *et al*, 2021) to only visualize chromatin-bound proteins. All cells were fixed with 2% paraformaldehyde in PBS for 20 min at room temperature, washed twice with PBS, permeabilized with 0.5% Triton-X in PBS for 10 min at room temperature, washed twice with PBS, and blocked for 1 h with 5% BSA in PBST. Staining with first antibodies (Reagents and Tools Table) was performed overnight at 4°C in 5% BSA in PBST. After three washes with 3% BSA in PBS, staining with mouse-AF568 secondary antibody (diluted 1:2,000, A11004 Molecular Probes) was performed for 1 h at room temperature. After three washes with 3% BSA in PBS and one wash with PBS, followed by DAPI or 5 µg/ml Hoechst 33342 (Life Technologies) staining and washes with PBS, cells were imaged.

Intracellular ROS was measured with CellROX green (Life Technologies), which exhibits bright fluorescence after oxidation and binding to DNA, thus allowing detection of nuclear and mitochondrial ROS, and mitochondria were stained with Mitotracker Deep Red FM (Life Technologies) according to the manufacturer's directions.

Imaging was performed either with an Opera or Operetta High Content Screening System (Perkin Elmer), using the ×40 magnification for quantification, an Olympus IXplore SpinSR spinning disk confocal microscope, using the ×60 magnification, or a Zeiss LSM700 confocal microscope using the ×63 magnification, as indicated in the Figure legends. Segmentation of the nuclei using the DAPI or Hoechst channels and quantification of the number of foci per cell or integrated intensity of the nuclear signal was done using CellProfiler software version 4.1.3 or Harmony software (Perkin Elmer), as indicated in the Figure legends. Segmentation of the cytoplasm was done based on the Mitotracker signal using the "Find Cytoplasm" option in the Harmony software. When applicable, the threshold to identify positive cells was either the nuclear integrated intensity (pRPA32, pATR) or the number of foci (γH2AX, RPA32, RPA70, PCNA) of the top 5% untreated wild-type cells. Quantifications of immunofluorescence staining were performed blindly. Visualization was done with ImageJ Fiji (Schindelin *et al*, 2012) or the Harmony software.

For HCT116 and HEK293 imaging, cells were fixed on glass coverslips with 4% PFA in PBS for 10 min at 37°C, washed three times with TBS, and permeabilized for 10 min in PBS with 0.5% Triton X-100. The coverslips were then blocked with blocking buffer (PBS with 4% BSA and 0.1% Triton X-100) for 1 h at room temperature and stained overnight with anti-COX4 antibody ab16056 1:1,000 at 4°C in blocking buffer. The coverslips were then washed 3 times with PBST (PBS with 0.1% Triton X-100) and stained with secondary (Thermo A32732) at 1:400 for 1 h at room temperature. The slides were washed three times with PBST and mounted with ProLong™ Gold Antifade Mountant with DAPI (Thermo P36935). Images were taken with a Nikon A1R Ultra-Fast Spectral Scanning Confocal Microscope using a 60× objective.

Flow cytometry

For detection of γH2AX signal, trypsinized cells were fixed in 90% ice-cold methanol while vortexing and incubated for at least 30 min at 4°Cs on a rotation wheel before storage at −20°C or further processing. Cells were then washed with PBS, blocked in FACS buffer (PBS+ 2.5% FBS + 1 mM EDTA) and incubated with γH2AX antibody (1:600 in FACS buffer, 100 µl/1 million cells) overnight at 4°C on a rotation wheel. After washes with FACS buffer, cells were incubated with AF488-anti-mouse antibody (1:600 in FACS buffer, 100 µl/1 million cells) for 1 h at room temperature. After washes with FACS buffer, DNA content was stained by PI solution (25 µg/ml PI +200 µg/ml RNase A in PBS, 10–20 million cells/ml). Cells were incubated for 10–15 min at room temperature and stored on ice until flow cytometry acquisition within 3–4 h.

© 2023 The Authors

For determining cell cycle profiles only, methanol-fixed cells were directly washed with PBS and incubated with the PI solution.

For the detection of apoptosis, the Pacific Blue™ Annexin V Apoptosis Detection Kit with PI (BioLegend) was used, following the manufacturer's instructions.

Cells were analyzed using a BD LSR-Fortessa X-20. Gating and cell cycle analysis were performed using FlowJo (v10).

Competitive growth assay

U2-OS WT and sgPRDX1 (population) and HT1080 WT and PRDX1$^{-/-}$ (clone) cells were transduced with pKAM-GFP plasmid and the GFP^{+} population was sorted using a BD FACSMelody. WT untagged cells and PRDX1-deficient GFP-tagged cells, or the opposite, were mixed together in equal amounts, and the percentage of GFP-positive cells at Day 0 was assessed by analyzing an aliquot with flow cytometry. Then, cells were harvested and re-seeded every 3 days for 12 days, each time analyzing an aliquot with flow cytometry to measure the percentage of GFP-positive cells. For the competitive growth assay with aspartate treatment, treated cells were grown in a growth medium containing 2 mM aspartate from Day 0, which was renewed every 3 days. Results were normalized to Day 0. Each experiment was performed in technical duplicates or triplicates and biological triplicates.

Cell cycle analysis

A stable U2-OS cell line with a Fluorescent Ubiquitination-based Cell Cycle Indicator (FUCCI) system was generated. The FUCCI system used is an adaptation of FUCCI4, to show 3 cell cycle-regulated fusion proteins: Clover-Geminin, SLBP-Turquoise2, and Cdt1-mKO2 (Bajar *et al*, 2016). For PRDX1 tracking over the cell cycle, U2-OS FUCCI cells were seeded in 96-well plates at 5,000 cells/well confluence, incubated for 48 h, and fixed for 10 min with formaldehyde 4%. Immunofluorescence was performed with primary PRDX1 antibody (ab109506) and secondary Alexa-647 (ab150167). Fluorescence from live or immunofluorescence preparations was measured with Operetta High Content Screening System (Perkin Elmer), using the ×20 magnification for quantification. Cell cycle determination based on fluorescence from the FUCCI proteins was performed by using a custom R script.

DNA replication fiber assay

Cells were pulsed with 25 uM CIdU (Sigma, C6891) for 20 min, washed repeatedly with PBS, and treated with 1uM Etoposide for 3 h, washed with PBS and pulsed with 250 μM IdU (Sigma, I7125). Cells were resuspended in ice-cold PBS, and 2 μl of the cell solution was transferred to a microscope slide and incubated with 7 μl of spreading buffer (200 mM Tris–HCl pH 7.5, 50 mM EDTA and 0.5% SDS) for 2 min. DNA was spread by tilting the slides. Fixation was performed with methanol:acetic acid (3:1) for 10 min. DNA was denatured in 2.5 M HCl for 1 h at RT, rinsed in PBS, and blocked in 1% BSA, 0.1% Triton X-100 in PBS for 1 h at RT before staining with primary antibodies overnight at 4°C: Rat anti-BrdU (MCA6144) to detect CldU, and mouse anti-BrdU (Becton Dickinson, 347580) to detect IdU. Alexa Fluor-conjugated antibodies (Invitrogen) were incubated for 1,5 h at 37°C and after several washes, mounted in mounting media (Thermofisher 00-4958-02). Tracks were imaged on a Leica dmi6000b microscope at 63×, images were blinded and analyzed using Fiji software and fork rate was calculated using ((length (mm) × 2.59 kb/mm)/pulse time (min)). At least 60 tracks were analyzed in every experiment.

ETC drug treatments

U2-OS cells were seeded in black Cellcarrier-96-well plates at 3,000 cells/well rate and incubated overnight for attachment. The cells were treated with Phenformin (HY-16397A, MedChem Express) or TTFA (ab223880, Abcam) at the indicated concentrations, in combination either with 1 μM etoposide or DMSO (negative control, drug solvent) for 3 h. Then, the media was removed and cells were washed with PBS prior to adding fresh media with the same concentrations of Phenformin and TTFA. Cells were incubated for additional 96 h. Before quantifying cell numbers with the Harmony software, plates were fixed and DAPI staining was performed as described in the immunofluorescence microscopy section in order to calculate the drugs' IC50.

Etoposide and carboplatin IC50 calculation

U2-OS PRDX1-depleted and control cells (shPRDX1 & shNTC) were seeded in black Cellcarrier-96-well plates at 2,000 cells/well ratio and incubated overnight for attachment. The cells were treated with etoposide or carboplatin (HY-17393, MedChem Express) at the indicated concentrations for 96 h. Plates were fixed and DAPI stained as described in the immunofluorescence microscopy section prior to cell number quantification.

Metabolite treatments

Ascorbic acid and aspartate

U2-OS PRDX1-depleted and control cells (shPRDX1 & shNTC) were seeded in black Cellcarrier-96-well plates at 2,000 cells/well ratio and incubated overnight for attachment. Cells were supplemented with Aspartate 2 mM (A7219, Merck), Ascorbic acid 1 μM (A4544, Merck), alone or in combination. Plates were fixed for 15 min with formaldehyde 4%. DAPI staining and immunofluorescence were performed with primary γH2AX antibody (05-636, Merck) and secondary Alexa-555 (A-21424). Fluorescence was measured with Operetta High Content Screening System (Perkin Elmer), using the x20 magnification for quantification.

Nucleotides

U2-OS PRDX1-depleted and control cells (shPRDX1 & shNTC) were seeded in black Cellcarrier-96-well plates at 2,000 cells/well and incubated overnight for attachment. The cells were treated for 72 h with fresh media supplemented with nucleotides, each at 100 μM (R0451, R0471, R0461, R0441, Thermo Scientific). Next, 1 μM etoposide or DMSO (negative control, drug solvent) was added for 3 h following which cells were washed with PBS, and incubated with fresh media supplemented with nucleotides (100 μM each). Plates were fixed for 15 min with formaldehyde 4% at time points 0, 2, and 4 h. Immunofluorescence was performed with primary γH2AX antibody (05-636, Merck) and secondary Alexa-555 (A-21424). Nuclei were visualized with DAPI staining.

Gene ontology-term analysis

Statistical tests for enrichment were performed using the GSEA function in the clusterProfiler R package (Wu *et al*, 2021). To remove redundant terms, due to shared genes, terms were eliminated when they had a high Jaccard Index (larger than 0.3).

© 2023 The Authors

Statistical analysis

Statistical parameters including the exact value of n (e.g., the total number of experiments, measured cells), deviations, P-values, and type of statistical test are reported in the respective Figure captions. Statistical analysis was performed across biological replicates, by taking the average of the respective technical replicates, when appropriate. Error bars displayed in graphs represent the mean and standard error of the mean (SEM) of at least three biologically independent experiments. Statistical significance was analyzed using paired two-tailed Student's t-test after testing for normality (Shapiro test) and equal variance (Levene test) or nonparametric Wilcoxon test. $P < 0.05$ was considered significant. In all cases, ns: not significant ($P > 0.05$), $^{*}P < 0.05$, $^{**}P < 0.01$, $^{***}P < 0.001$, $^{****}P < 0.0001$. For the Harmony image quantification, linear regression models were fitted on the log2 integrated intensities to account for both variations in the technical variation between replicates and the biological differences between treatments. For the metabolomics PRDX1-etoposide dependency, a linear regression model was fit for each of the etoposide transitions (treatment, early release, recovery).

Data availability

The datasets and computer code produced in this study are available in the following databases:

- CRISPR screen next generation sequencing data: ENA ERA16463919 (https://www.ebi.ac.uk/ena/browser/view/PRJEB54700).
- Chromatome-MS data: PRIDE PXD035532 (http://www.ebi.ac.uk/pride/archive/projects/PXD035532)
- Chromatome-MS analysis code: https://github.com/SdelciLab/PRDX1_DDR
- Metabolomics data: Metabolomics Workbench ST002234 (https://www.metabolomicsworkbench.org/data/DRCCMetadata.php?Mode=Study&StudyID=ST002234).

Expanded View for this article is available online.

Acknowledgments

We are thankful to Prof J Lingner (Swiss Institute for Experimental Cancer Research (ISREC), Ecole Polytechnique Fédérale de Lausanne [EPFL]) for providing the HT1080 wild-type and PRDX1-deficient cell lines, and to Prof Sebastian M Nijman (Ludwig Cancer Research, Oxford, UK) for providing the pLKO.2 plasmid. We would like to thank the Biomedical Sequencing Facility (CeMM, Vienna, Austria) for all next-generation sequencing and the Metabolomics Facility (CeMM, Vienna, Austria) for the metabolomics analysis. We thank Gerald Timelthaler (Institute of Cancer Research, Imaging Facility) for assistance with the microscopy. We are grateful to Joana Ferreira da Silva and all other members of the Loizou lab, as well as all members of the Sdelci lab, for helpful discussions and feedback.

AM and CC were funded by the Austrian Science Fund (grant number P 33024 awarded to JIL). The Loizou lab is funded by an ERC Synergy Grant (DDREAMM Grant agreement ID: 855741). The Sdelci lab's contributions to this study were funded by an ERC Starting Grant (ERC-StG-852343-EPICAMENTE). This work was funded, in part, by a donation from Benjamin Landesmann. The funder was not involved in the study design, collection, analysis, interpretation of data, the writing of this article, or the decision to submit it for publication. CeMM is funded by the Austrian Academy of Sciences. MGVH acknowledges funding from R35CA242379, the Lustgarten Foundation, the Ludwig Center at MIT, and the MIT Center for Precision Cancer Medicine.

Author contributions

Amandine Moretton: Conceptualization; formal analysis; investigation; visualization; methodology; writing – original draft; writing – review and editing. **Savvas Kourtis:** Resources; data curation; formal analysis; validation; investigation; visualization; methodology; writing – original draft; writing – review and editing. **Antoni Gañez Zapater:** Formal analysis; validation; investigation; visualization; methodology; writing – original draft; writing – review and editing. **Chiara Calabrò:** Formal analysis; validation; investigation; writing – original draft. **Maria Lorena Espinar Calvo:** Validation; investigation; writing – original draft; writing – review and editing. **Frédéric Fontaine:** Data curation; formal analysis; investigation; writing – original draft. **Evangelia Darai:** Formal analysis; validation; investigation; writing – review and editing. **Etna Abad Cortel:** Formal analysis; investigation. **Samuel Block:** Formal analysis; investigation; visualization; writing – review and editing. **Laura Pascual-Reguant:** Formal analysis; investigation; writing – review and editing. **Natalia Pardo-Lorente:** Formal analysis; methodology; writing – review and editing. **Ritobrata Ghose:** Formal analysis; methodology; writing – review and editing. **Matthew G Vander Heiden:** Supervision. **Ana Janic:** Formal analysis; supervision; writing – review and editing. **André C Müller:** Data curation; formal analysis; investigation; methodology; writing – original draft. **Joanna I Loizou:** Conceptualization; supervision; funding acquisition; methodology; writing – original draft; project administration; writing – review and editing. **Sara Sdelci:** Conceptualization; supervision; funding acquisition; visualization; methodology; writing – original draft; project administration; writing – review and editing.

In addition to the CRediT author contributions listed above, the contributions in detail are:

AMo, SS, and JIL conceptualized the study. SS and JIL obtained funding. AMo, CC, and AGZ carried out all cell-based investigations. ED, EAC, SDB, LPR, NPL, and RG supported the cell-based investigation and its analysis. SK performed all bioinformatics investigations. MLEC cloned the FUCCI system and performed the metabolite treatment experiments. FF and AMü performed and analyzed the chromatome proteomics experiment. AMo and SK performed analysis and visualization. AJ and MVH supervised selected experiments. SS and JIL supervised the whole study. AMo and SS wrote the original draft and all authors reviewed and edited the final manuscript.

Disclosure and competing interests statement

The authors declare that they have no conflict of interest. MGVH discloses that he is a scientific advisor for Agios Pharmaceuticals, iTeos Therapeutics, Sage Therapeutics, Pretzel Therapeutics, Lime Therapeutics, Droia Ventures, and Auron Therapeutics. JIL is currently an employee of AstraZeneca.

References

Aeby E, Ahmed W, Redon S, Simanis V, Lingner J (2016) Peroxiredoxin 1 protects telomeres from oxidative damage and preserves telomeric DNA for extension by telomerase. *Cell Rep* 17: 3107–3114

Ahlmann-Eltze C (2022) proDA: differential abundance analysis of label-free mass spectrometry data. *Bioconductor* R package version 1.10.0

Ahmed W, Lingner J (2018) PRDX1 and MTH1 cooperate to prevent ROS-mediated inhibition of telomerase. *Genes Dev* 32: 658–669

 © 2023 The Authors

Alkan HF, Walter KE, Luengo A, Madreiter-Sokolowski CT, Stryeck S, Lau AN, Al-Zoughbi W, Lewis CA, Thomas CJ, Hoefler G *et al* (2018) Cytosolic aspartate availability determines cell survival when glutamine is limiting. *Cell Metab* 28: 706 – 720

Arslan T (2021) SubCellBarCode: integrated workflow for robust mapping and visualizing whole human spatial proteome. *Bioconductor* R package version 1.8.0

Badia-i-Mompel P, Vélez Santiago J, Braunger J, Geiss C, Dimitrov D, Müller-Dott S, Taus P, Dugourd A, Holland CH, Ramirez Flores RO *et al* (2022) decoupleR: ensemble of computational methods to infer biological activities from omics data. *Bioinformatics Adv* 2: vbac016

Bajar BT, Lam AJ, Badiee RK, Oh YH, Chu J, Zhou XX, Kim N, Kim BB, Chung M, Yablonovitch AL *et al* (2016) Fluorescent indicators for simultaneous reporting of all four cell cycle phases. *Nat Methods* 13: 993 – 996

Bajor M, Zych AO, Graczyk-Jarzynka A, Muchowicz A, Firczuk M, Trzeciak L, Gaj P, Domagala A, Siernicka M, Zagozdzon A *et al* (2018) Targeting peroxiredoxin 1 impairs growth of breast cancer cells and potently sensitises these cells to prooxidant agents. *Br J Cancer* 119: 873 – 884

Bester AC, Roniger M, Oren YS, Im MM, Sarni D, Chaoat M, Bensimon A, Zamir G, Shewach DS, Kerem B (2011) Nucleotide deficiency promotes genomic instability in early stages of cancer development. *Cell* 145: 435 – 446

Birsoy K, Wang T, Chen WW, Freinkman E, Abu-Remaileh M, Sabatini DM (2015) An essential role of the mitochondrial electron transport chain in cell proliferation is to enable aspartate synthesis. *Cell* 162: 540 – 551

Buckland RJ, Watt DL, Chittoor B, Nilsson AK, Kunkel TA, Chabes A (2014) Increased and imbalanced dNTP pools symmetrically promote both leading and lagging strand replication infidelity. *PLoS Genet* 10: e1004846

Cortez D (2015) Preventing replication fork collapse to maintain genome integrity. *DNA Repair (Amst)* 32: 149 – 157

Cross BC, Lawo S, Archer CR, Hunt JR, Yarker JL, Riccombeni A, Little AS, McCarthy NJ, Moore JD (2016) Increasing the performance of pooled CRISPR-Cas9 drop-out screening. *Sci Rep* 6: 31782

Dai L, Dai Y, Han J, Huang Y, Wang L, Huang J, Zhou Z (2021) Structural insight into BRCA1-BARD1 complex recruitment to damaged chromatin. *Mol Cell* 81: e2766

D'Angiolella V, Donato V, Forrester FM, Jeong YT, Pellacani C, Kudo Y, Saraf A, Florens L, Washburn MP, Pagano M (2012) Cyclin F-mediated degradation of ribonucleotide reductase M2 controls genome integrity and DNA repair. *Cell* 149: 1023 – 1034

Del Cano-Ochoa F, Moreno-Morcillo M, Ramon-Maiques S (2019) CAD, a multienzymatic protein at the head of de novo pyrimidine biosynthesis. *Subcell Biochem* 93: 505 – 538

Dempster JM, Rossen J, Kazachkova M, Pan J, Kugener G, Root DE, Tsherniak A (2019) Extracting biological insights from the project achilles genome-scale crispr screens in cancer cell lines. *bioRxiv* https://doi.org/10.1101/720243 [PREPRINT]

Diehl FF, Miettinen TP, Elbashir R, Nabel CS, Darnell AM, Do BT, Manalis SR, Lewis CA, Vander Heiden MG (2022) Nucleotide imbalance decouples cell growth from cell proliferation. *Nat Cell Biol* 24: 1252 – 1264

Dizdaroglu M (1992) Oxidative damage to DNA in mammalian chromatin. *Mutat Res* 275: 331 – 342

Egler RA, Fernandes E, Rothermund K, Sereika S, de Souza-Pinto N, Jaruga P, Dizdaroglu M, Prochownik EV (2005) Regulation of reactive oxygen species, DNA damage, and c-Myc function by peroxiredoxin 1. *Oncogene* 24: 8038 – 8050

Essers J, Theil AF, Baldeyron C, van Cappellen WA, Houtsmuller AB, Kanaar R, Vermeulen W (2005) Nuclear dynamics of PCNA in DNA replication and repair. *Mol Cell Biol* 25: 9350 – 9359

Feng X, Ma D, Zhao J, Song Y, Zhu Y, Zhou Q, Ma F, Liu X, Zhong M, Liu Y *et al* (2020) UHMK1 promotes gastric cancer progression through reprogramming nucleotide metabolism. *EMBO J* 39: e102541

Gao S, Li H, Feng XJ, Li M, Liu ZP, Cai Y, Lu J, Huang XY, Wang JJ, Li Q *et al* (2015) Alpha-enolase plays a catalytically independent role in doxorubicin-induced cardiomyocyte apoptosis and mitochondrial dysfunction. *J Mol Cell Cardiol* 79: 92 – 103

Garcia-Bermudez J, Baudrier L, La K, Zhu XG, Fidelin J, Sviderskiy VO, Papagiannakopoulos T, Molina H, Snuderl M, Lewis CA *et al* (2018) Aspartate is a limiting metabolite for cancer cell proliferation under hypoxia and in tumours. *Nat Cell Biol* 20: 775 – 781

Ghandi M, Huang FW, Jane-Valbuena J, Kryukov GV, Lo CC, McDonald ER 3rd, Barretina J, Gelfand ET, Bielski CM, Li H *et al* (2019) Next-generation characterization of the cancer cell line encyclopedia. *Nature* 569: 503 – 508

Gianetto QG, Wieczorek S, Couté Y, Burger T (2020) A peptide-level multiple imputation strategy accounting for the different natures of missing values in proteomics data. *bioRxiv* https://doi.org/10.1101/2020.05.29.122770 [PREPRINT]

Giorgio M, Trinei M, Migliaccio E, Pelicci PG (2007) Hydrogen peroxide: a metabolic by-product or a common mediator of ageing signals? *Nat Rev Mol Cell Biol* 8: 722 – 728

Gracheva E, Chitale S, Wilhelm T, Rapp A, Byrne J, Stadler J, Medina R, Cardoso MC, Richly H (2016) ZRF1 mediates remodeling of E3 ligases at DNA lesion sites during nucleotide excision repair. *J Cell Biol* 213: 185 – 200

Hande KR (1998) Etoposide: four decades of development of a topoisomerase II inhibitor. *Eur J Cancer* 34: 1514 – 1521

Hansen JM, Moriarty-Craige S, Jones DP (2007) Nuclear and cytoplasmic peroxiredoxin-1 differentially regulate NF-kappaB activities. *Free Radic Biol Med* 43: 282 – 288

Harris IS, Treloar AE, Inoue S, Sasaki M, Gorrini C, Lee KC, Yung KY, Brenner D, Knobbe-Thomsen CB, Cox MA *et al* (2015) Glutathione and thioredoxin antioxidant pathways synergize to drive cancer initiation and progression. *Cancer Cell* 27: 211 – 222

Hoeijmakers JH (2001) Genome maintenance mechanisms for preventing cancer. *Nature* 411: 366 – 374

Huang J, Huen MS, Kim H, Leung CC, Glover JN, Yu X, Chen J (2009) RAD18 transmits DNA damage signalling to elicit homologous recombination repair. *Nat Cell Biol* 11: 592 – 603

Hughes CS, Foehr S, Garfield DA, Furlong EE, Steinmetz LM, Krijgsveld J (2014) Ultrasensitive proteome analysis using paramagnetic bead technology. *Mol Syst Biol* 10: 757

Hume S, Grou CP, Lascaux P, D'Angiolella V, Legrand AJ, Ramadan K, Dianov GL (2021) The NUCKS1-SKP2-p21/p27 axis controls S phase entry. *Nat Commun* 12: 6959

Hussein D, Estlin EJ, Dive C, Makin GW (2006) Chronic hypoxia promotes hypoxia-inducible factor-1alpha-dependent resistance to etoposide and vincristine in neuroblastoma cells. *Mol Cancer Ther* 5: 2241 – 2250

Jackson SP, Bartek J (2009) The DNA-damage response in human biology and disease. *Nature* 461: 1071 – 1078

Jin L, Williamson A, Banerjee S, Philipp I, Rape M (2008) Mechanism of ubiquitin-chain formation by the human anaphase-promoting complex. *Cell* 133: 653 – 665

Kanehisa M, Goto S (2000) KEGG: Kyoto encyclopedia of genes and genomes. *Nucleic Acids Res* 28: 27 – 30

© 2023 The Authors

Kim JW, Tchernyshyov I, Semenza GL, Dang CV (2006) HIF-1-mediated expression of pyruvate dehydrogenase kinase: a metabolic switch required for cellular adaptation to hypoxia. *Cell Metab* 3: 177 – 185

Kim SJ, Kim HS, Seo YR (2019) Understanding of ROS-inducing strategy in anticancer therapy. *Oxid Med Cell Longev* 2019: 5381692

Kirova DG, Judasova K, Vorhauser J, Zerjatke T, Leung JK, Glauche I, Mansfeld J (2022) A ROS-dependent mechanism promotes CDK2 phosphorylation to drive progression through S phase. *Dev Cell* 57: 1712 – 1727

Leek JT, Johnson WE, Parker HS, Jaffe AE, Storey JD (2012) The sva package for removing batch effects and other unwanted variation in high-throughput experiments. *Bioinformatics* 28: 882 – 883

Li W, Xu H, Xiao T, Cong L, Love MI, Zhang F, Irizarry RA, Liu JS, Brown M, Liu XS (2014) MAGeCK enables robust identification of essential genes from genome-scale CRISPR/Cas9 knockout screens. *Genome Biol* 15: 554

Ma J, Zhong M, Xiong Y, Gao Z, Wu Z, Liu Y, Hong X (2021) Emerging roles of nucleotide metabolism in cancer development: progress and prospect. *Aging (Albany NY)* 13: 13349 – 13358

Marx C, Sonnemann J, Maddocks ODK, Marx-Blumel L, Beyer M, Hoelzer D, Thierbach R, Maletzki C, Linnebacher M, Heinzel T *et al* (2022) Global metabolic alterations in colorectal cancer cells during irinotecan-induced DNA replication stress. *Cancer Metab* 10: 10

Moretton A, Loizou JI (2020) Interplay between cellular metabolism and the DNA damage response in cancer. *Cancers (Basel)* 12: 2051

Morinaka A, Funato Y, Uesugi K, Miki H (2011) Oligomeric peroxiredoxin-I is an essential intermediate for p53 to activate MST1 kinase and apoptosis. *Oncogene* 30: 4208 – 4218

Movafagh S, Crook S, Vo K (2015) Regulation of hypoxia-inducible factor-1a by reactive oxygen species: new developments in an old debate. *J Cell Biochem* 116: 696 – 703

Mu ZM, Yin XY, Prochownik EV (2002) Pag, a putative tumor suppressor, interacts with the Myc box II domain of c-Myc and selectively alters its biological function and target gene expression. *J Biol Chem* 277: 43175 – 43184

Nakamura J, Mutlu E, Sharma V, Collins L, Bodnar W, Yu R, Lai Y, Moeller B, Lu K, Swenberg J (2014) The endogenous exposome. *DNA Repair (Amst)* 19: 3 – 13

Neumann CA, Krause DS, Carman CV, Das S, Dubey DP, Abraham JL, Bronson RT, Fujiwara Y, Orkin SH, Van Etten RA (2003) Essential role for the peroxiredoxin Prdx1 in erythrocyte antioxidant defence and tumour suppression. *Nature* 424: 561 – 565

Neumann CA, Cao J, Manevich Y (2009) Peroxiredoxin 1 and its role in cell signaling. *Cell Cycle* 8: 4072 – 4078

Nishimoto A, Kugimiya N, Hosoyama T, Enoki T, Li TS, Hamano K (2014) HIF-1alpha activation under glucose deprivation plays a central role in the acquisition of anti-apoptosis in human colon cancer cells. *Int J Oncol* 44: 2077 – 2084

Olsen JV, de Godoy LM, Li G, Macek B, Mortensen P, Pesch R, Makarov A, Lange O, Horning S, Mann M (2005) Parts per million mass accuracy on an orbitrap mass spectrometer via lock mass injection into a C-trap. *Mol Cell Proteomics* 4: 2010 – 2021

O'Sullivan J, Mersaoui SY, Poirier G, Masson JY (2021) Assessment of global DNA double-Strand end resection using BrdU-DNA labeling coupled with cell cycle discrimination imaging. *J Vis Exp* 170: 62553

Paredes F, Williams HC, San Martin A (2021) Metabolic adaptation in hypoxia and cancer. *Cancer Lett* 502: 133 – 142

Pareek V, Pedley AM, Benkovic SJ (2021) Human de novo purine biosynthesis. *Crit Rev Biochem Mol Biol* 56: 1 – 16

Parplys AC, Zhao W, Sharma N, Groesser T, Liang F, Maranon DG, Leung SG, Grundt K, Dray E, Idate R *et al* (2015) NUCKS1 is a novel RAD51AP1 paralog important for homologous recombination and genome stability. *Nucleic Acids Res* 43: 9817 – 9834

Perez-Riverol Y, Bai J, Bandla C, Hewapathirana S, García-Seisdedos D, Kamatchinathan S, Kundu D, Prakash A, Frericks-Zipper A, Eisenacher M *et al* (2022) The PRIDE database resources in 2022: a hub for mass spectrometry-based proteomics evidences. *Nucleic Acids Res* 50: D543 – D552

Rabinovich S, Adler L, Yizhak K, Sarver A, Silberman A, Agron S, Stettner N, Sun Q, Brandis A, Helbling D *et al* (2015) Diversion of aspartate in ASS1-deficient tumours fosters *de novo* pyrimidine synthesis. *Nature* 527: 379 – 383

Robinson JL, Kocabas P, Wang H, Cholley PE, Cook D, Nilsson A, Anton M, Ferreira R, Domenzain I, Billa V *et al* (2020) An atlas of human metabolism. *Sci Signal* 13: eaaz1482

Schindelin J, Arganda-Carreras I, Frise E, Kaynig V, Longair M, Pietzsch T, Preibisch S, Rueden C, Saalfeld S, Schmid B *et al* (2012) Fiji: an open-source platform for biological-image analysis. *Nat Methods* 9: 676 – 682

Schrimpe-Rutledge AC, Codreanu SG, Sherrod SD, McLean JA (2016) Untargeted metabolomics strategies-challenges and emerging directions. *J Am Soc Mass Spectrom* 27: 1897 – 1905

Semenza GL (2007) Life with oxygen. *Science* 318: 62 – 64

Sharma A, Singh K, Almasan A (2012) Histone H2AX phosphorylation: a marker for DNA damage. *Methods Mol Biol* 920: 613 – 626

Shen W, Borchert AJ, Downs DM (2022) 2-Aminoacrylate stress damages diverse PLP-dependent enzymes in vivo. *J Biol Chem* 298: 101970

Shi D, Caldovic L, Tuchman M (2018) Sources and fates of Carbamyl phosphate: a labile energy-rich molecule with multiple facets. *Biology (Basel)* 7: 34

Shin HJ, Kwon HK, Lee JH, Anwar MA, Choi S (2016) Etoposide induced cytotoxicity mediated by ROS and ERK in human kidney proximal tubule cells. *Sci Rep* 6: 34064

Sies H, Jones DP (2020) Reactive oxygen species (ROS) as pleiotropic physiological signalling agents. *Nat Rev Mol Cell Biol* 21: 363 – 383

Sivanand S, Rhoades S, Jiang Q, Lee JV, Benci J, Zhang J, Yuan S, Viney I, Zhao S, Carrer A *et al* (2017) Nuclear acetyl-CoA production by ACLY promotes homologous recombination. *Mol Cell* 67: 252 – 265

Soniat MM, Myler LR, Kuo HC, Paull TT, Finkelstein IJ (2019) RPA phosphorylation inhibits DNA resection. *Mol Cell* 75: 145 – 153

Stirling DR, Swain-Bowden MJ, Lucas AM, Carpenter AE, Cimini BA, Goodman A (2021) CellProfiler 4: improvements in speed, utility and usability. *BMC Bioinformatics* 22: 433

Sulkowski PL, Oeck S, Dow J, Economos NG, Mirfakhraie L, Liu Y, Noronha K, Bao X, Li J, Shuch BM *et al* (2020) Oncometabolites suppress DNA repair by disrupting local chromatin signalling. *Nature* 582: 586 – 591

Sullivan LB, Gui DY, Hosios AM, Bush LN, Freinkman E, Vander Heiden MG (2015) Supporting aspartate biosynthesis is an essential function of respiration in proliferating cells. *Cell* 162: 552 – 563

Sullivan LB, Luengo A, Danai LV, Bush LN, Diehl FF, Hosios AM, Lau AN, Elmiligy S, Malstrom S, Lewis CA *et al* (2018) Aspartate is an endogenous metabolic limitation for tumour growth. *Nat Cell Biol* 20: 782 – 788

Thul PJ, Åkesson L, Wiking M, Mahdessian D, Geladaki A, Ait Blal H, Alm T, Asplund A, Björk L, Breckels LM *et al* (2017) A subcellular map of the human proteome. *Science* 356: eaal3321

Tran TQ, Ishak Gabra MB, Lowman XH, Yang Y, Reid MA, Pan M, O'Connor TR, Kong M (2017) Glutamine deficiency induces DNA alkylation damage and sensitizes cancer cells to alkylating agents through inhibition of ALKBH enzymes. *PLoS Biol* 15: e2002810

Turgeon MO, Perry NJS, Poulogiannis G (2018) DNA damage, repair, and cancer metabolism. *Front Oncol* 8: 15

Tyanova S, Temu T, Sinitcyn P, Carlson A, Hein MY, Geiger T, Mann M, Cox J (2016) The Perseus computational platform for comprehensive analysis of (prote)omics data. *Nat Methods* 13: 731 – 740

 © 2023 The Authors

Wang Y, Wu Z (2021) The clinical significance and transcription regulation of a DNA damage repair gene, SMC4, in low-grade glioma via integrated bioinformatic analysis. *Front Oncol* 11: 761693

Wang P, Wu J, Ma S, Zhang L, Yao J, Hoadley KA, Wilkerson MD, Perou CM, Guan KL, Ye D *et al* (2015) Oncometabolite D-2-hydroxyglutarate inhibits ALKBH DNA repair enzymes and sensitizes IDH mutant cells to alkylating agents. *Cell Rep* 13: 2353 – 2361

Wang B, Wang M, Zhang W, Xiao T, Chen CH, Wu A, Wu F, Traugh N, Wang X, Li Z *et al* (2019) Integrative analysis of pooled CRISPR genetic screens using MAGeCKFlute. *Nat Protoc* 14: 756 – 780

Wang J, Sun D, Huang L, Wang S, Jin Y (2021) Targeting reactive oxygen species capacity of tumor cells with repurposed drug as an anticancer therapy. *Oxid Med Cell Longev* 2021: 8532940

Wickham H, Averick M, Bryan J, Chang W, McGowan L, François R, Grolemund G, Hayes A, Henry L, Hester J *et al* (2019) Welcome to the Tidyverse. *J Open Source Softw* 4: 1686

Wu T, Hu E, Xu S, Chen M, Guo P, Dai Z, Feng T, Zhou L, Tang W, Zhan L *et al* (2021) clusterProfiler 4.0: a universal enrichment tool for interpreting omics data. *Innovation (Camb)* 2: 100141

Yao Q, Khan MP, Merceron C, LaGory EL, Tata Z, Mangiavini L, Hu J, Vemulapalli K, Chandel NS, Giaccia AJ *et al* (2019) Suppressing mitochondrial respiration is critical for hypoxia tolerance in the fetal growth plate. *Dev Cell* 49: 748 – 763

Zhang X, Smits AH, van Tilburg GB, Ovaa H, Huber W, Vermeulen M (2018) Proteome-wide identification of ubiquitin interactions using UbIA-MS. *Nat Protoc* 13: 530 – 550

License: This is an open access article under the terms of the Creative Commons Attribution License, which permits use, distribution and reproduction in any medium, provided the original work is properly cited.

© 2023 The Authors

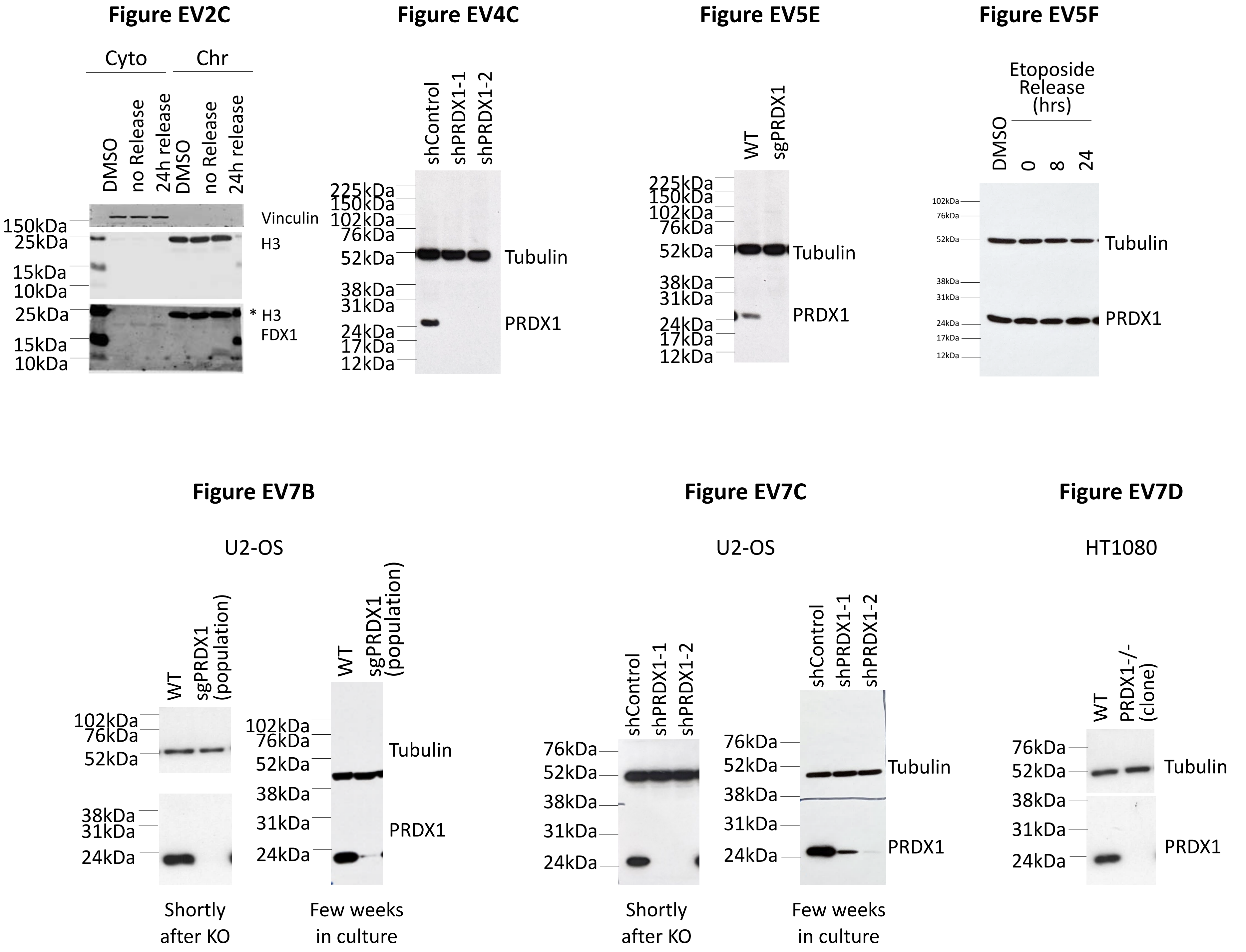

Appendix Figure S1: full size of the western blots presented in the manuscript. Cyto is cytoplasm, Chr is Chromatin

Expanded View Figures

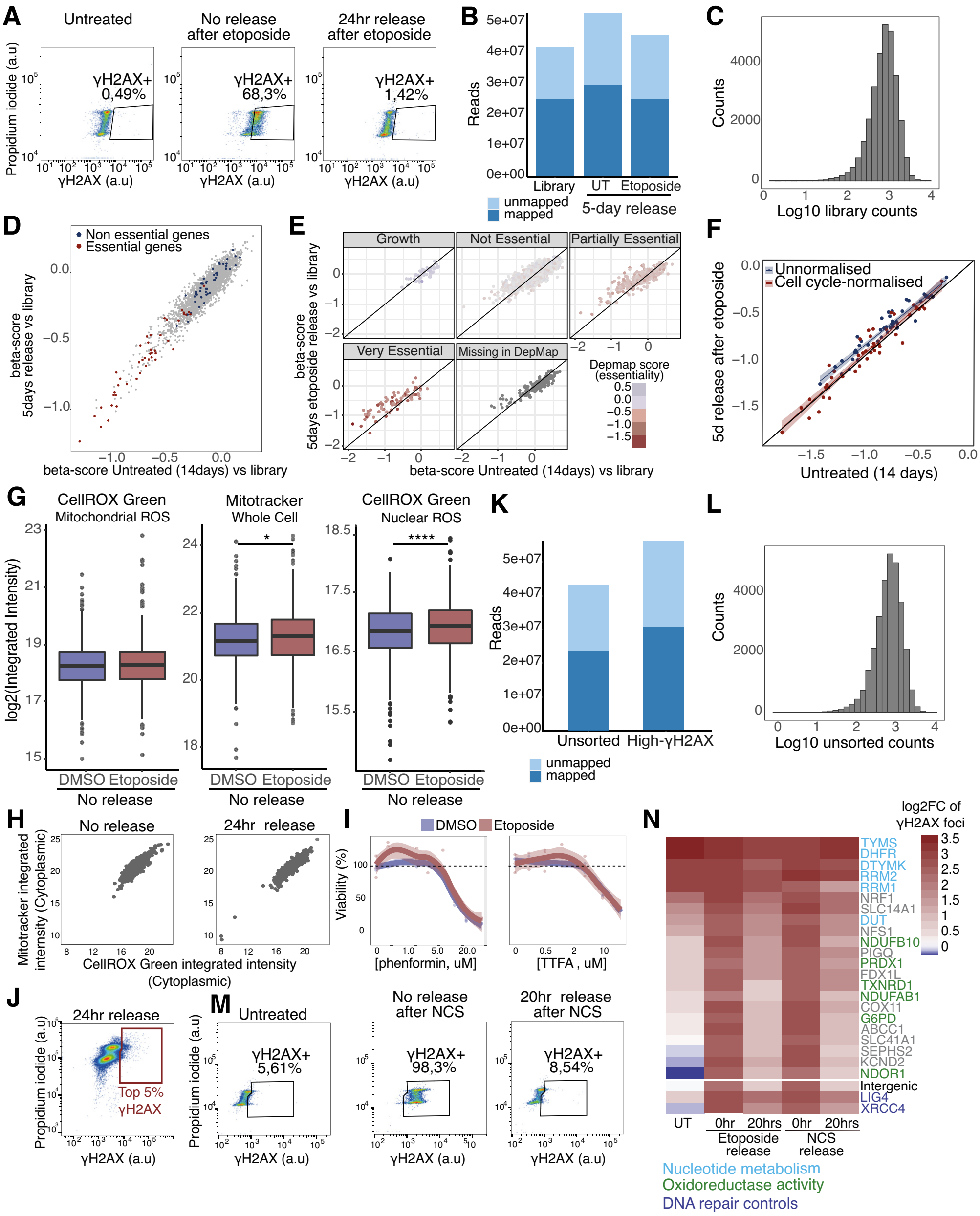

Figure EV1.

 © 2023 The Authors

◀ **Figure EV1. Etoposide-release CRISPR-Cas9 screens with a metabolic library.**

A FACS monitoring of γH2AX levels following etoposide treatment and after 24 h of release in drug-free media.

B Mapped reads in the etoposide survival CRISPR screen.

C Distribution of reads in the etoposide survival CRISPR screen.

D, E Comparison of β scores separated by gene essentiality according to MaGECKFlute (D) or DepMap (E).

F MaGECKflute cell cycle normalization based on essential genes. Shaded area represents the 95% confidence interval around the median (central line).

G Quantification of CellROX Green and Mitotracker stained U2-OS WT cells in DMSO- and etoposide-treated conditions, represented as nuclear or cytoplasmic integrated intensities of CellROX Green signal and whole cell integrated intensities of Mitotracker. Three biological replicates were performed. A minimum of 1,000 cells were quantified for each condition, using Harmony. Boxplots represent the median within the IQR. *P*-values were calculated using linear regression on the log2 normalized values where ns: not significant ($P > 0.05$), *$P < 0.05$, **$P < 0.01$, ***$P < 0.001$, ****$P < 0.0001$.

H Correlation of cytoplasmic CellROX Green, representing mitochondrial ROS, and Mitotracker integrated intensities of no release and 24 h release after etoposide in U2-OS cells. Three biological replicates were performed. A minimum of 1,000 cells (from three biological-replicate wells) were quantified for each condition, using Harmony.

I Viability of U2-OS cells treated with etoposide and increasing concentrations of various ETC inhibitors. Three biological replicates were performed. Shaded area represents the 95% confidence interval around the median (central line).

J FACS gating strategy for the high-γH2AX CRISPR screen.

K Mapped reads in the high-γH2AX CRISPR screen.

L Distribution of reads in the high-γH2AX CRISPR screen.

M FACS monitoring of γH2AX levels following NCS treatment and after 20 h of release in compound-free media.

N Quantification of the validation arrayed CRISPR screen using CellProfiler and represented as the mean of the log2 fold change compared to the untreated intergenic control of three independent biological replicates.

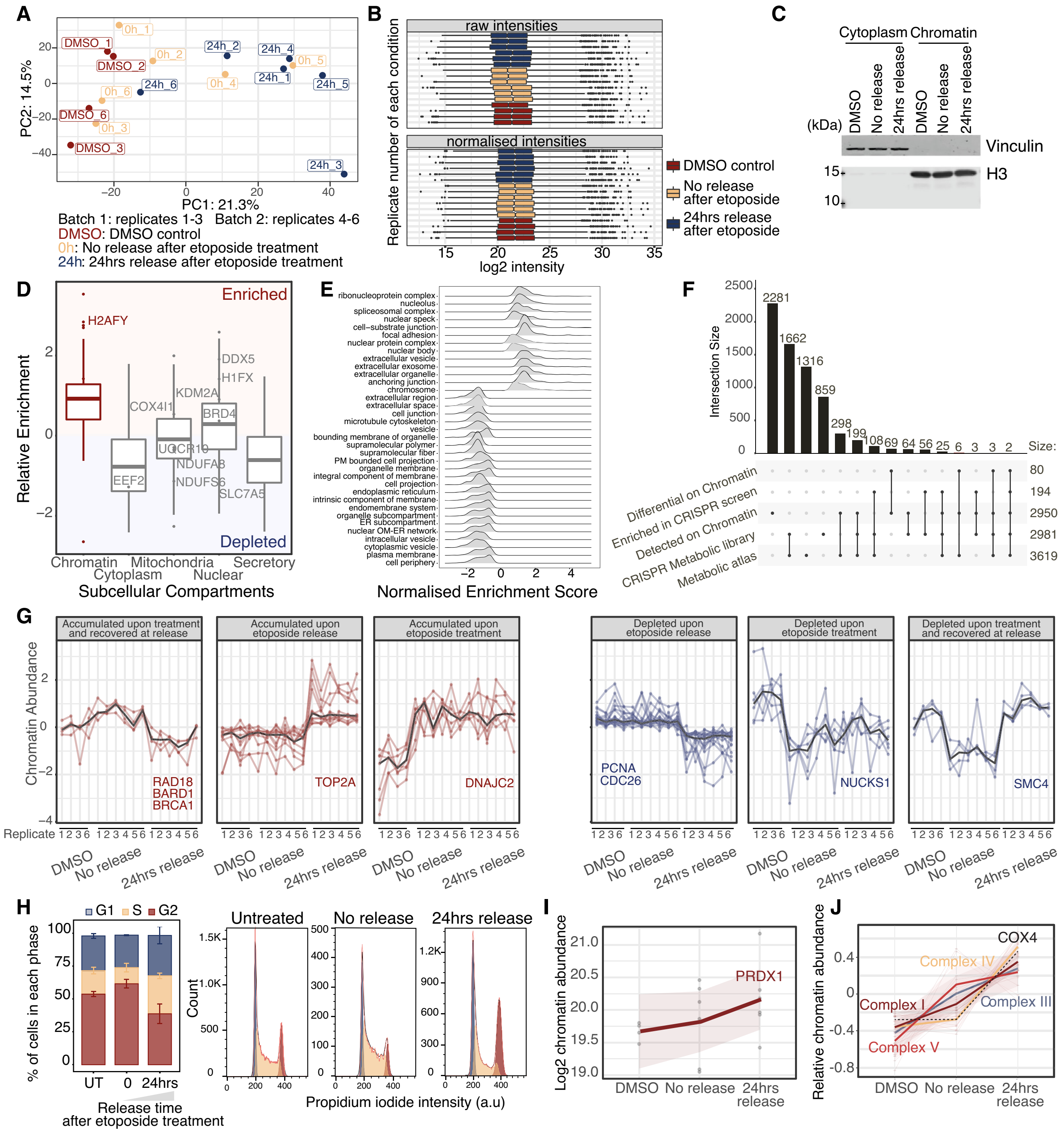

Figure EV2.

 © 2023 The Authors

Figure EV2. Etoposide-release chromatome proteomics.

A PCA of batch-corrected chromatin proteomics samples.
B Median normalization of raw proteomics intensities. Boxplots represent IQR around the median value for the samples shown in (A).
C Western blot confirmation of cell fractionation and chromatin enrichment for one representative replicate.
D Enrichment of known chromatin proteins normalized to publicly available U2-OS whole cell extract, based on the mean of at least four biological replicates. Boxplots represent IQR around the median.
E GSEA-Cellular Components of relative enrichment against publicly available U2-OS whole cell extract.
F Overlap of detected and significant genes between the chromatin and CRISPR-Cas9 screen datasets.
G Behavioral clustering of significant proteins with distinct chromatin recruitment patterns. Shaded area represents the 95% confidence interval around the median (central line).
H FACS monitoring cell cycle profiles after etoposide treatment and release of U2-OS WT cells.
I Kinetics of PRDX1 chromatin recruitment upon etoposide release. Shaded area represents the 95% confidence interval around the median (central line) for the biological samples shown in (A).
J Relative kinetics of members of ETC complexes chromatin recruitment upon etoposide release. Each protein is centered to its mean value. Shaded area represents the 95% confidence interval around the median (central line) for the biological samples shown in (A).

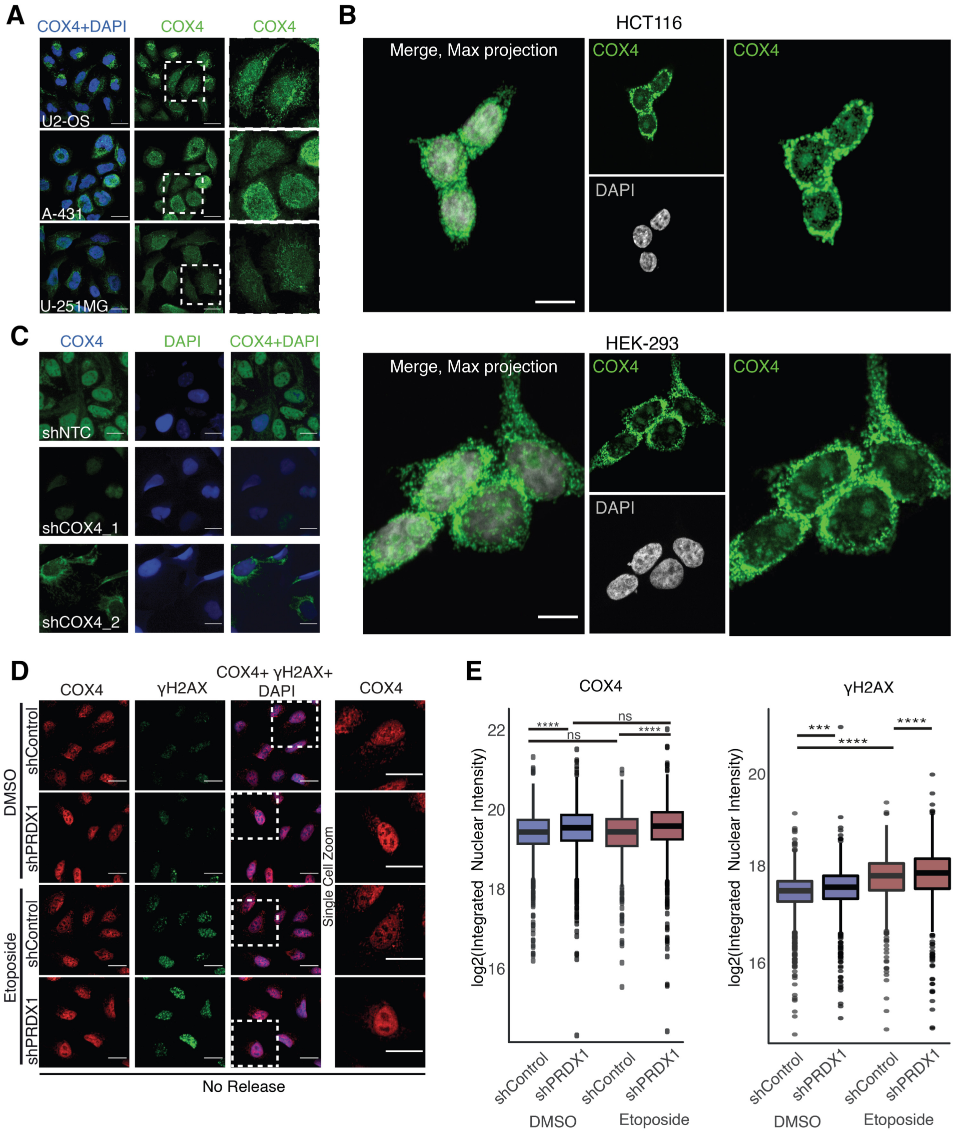

Figure EV3.

Figure EV3. COX4 nuclear presence.

A Visualization of COX4 (in green) and within DAPI stained nuclei (in blue) in multiple cell lines. Data obtained from the Human Protein Atlas, available from v22.0.proteinatlas.org. Scale bar is 25 μm.

B Visualization of COX4 (in green) within DAPI stained nuclei (in gray) in HCT116 and HEK293 cells. Images were acquired with a Nikon A1R Ultra-Fast Spectral Scanning Confocal Microscope using a 60× objective. Scale bar is 25 μm.

C Visualization of COX4 (in green) within DAPI stained nuclei (in blue) in U2-OS shControl and shCOX4 cells. Images were acquired with the Operetta High Content Screening System in confocal mode, scale bar is 25 μm.

D Visualization of COX4 (in red) and γH2AX (in green) within DAPI stained nuclei (in blue) in U2-OS shControl and shPRDX1 cells at the indicated treatment conditions. Images were acquired with the Operetta High Content Screening System in confocal mode, scale bar is 25 μm.

E Quantification of images shown in (D), represented as nuclear integrated intensities of γH2AX and COX4 signals. Three biological replicates were perfomed. A minimum of 1,000 cells were quantified for each condition, using Harmony. Boxplots represent the median within the IQR. *P*-values were calculated using linear regression on the log2 normalized values and the interaction term *P*-value between PRDX1 background and etoposide treatment is shown in the plot, where ns: not significant ($P > 0.05$), $^{*}P < 0.05$, $^{**}P < 0.01$, $^{***}P < 0.001$, $^{****}P < 0.0001$.

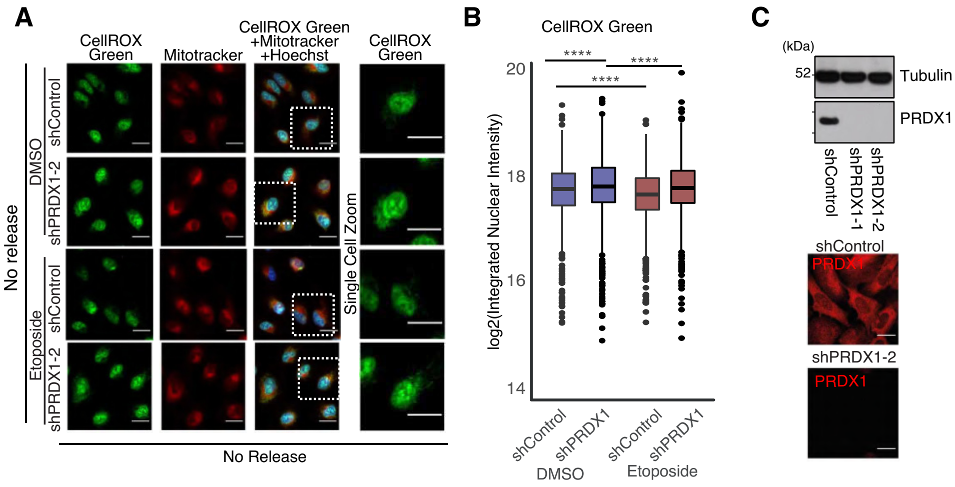

Figure EV4. Nuclear ROS accumulates in the absence of PRDX1.

A Visualization of ROS (CellROX Green, in green) and mitochondria (Mitotracker, in red) within Hoechst-stained nuclei (in blue) in U2-OS shControl and shPRDX1 cells at the indicated treatment conditions. Images were acquired with the Operetta High Content Screening System in confocal mode, scale bar is 25 μm.

B Quantification of images shown in (A), represented as log2 nuclear-integrated intensity of CellROX Green and Mitotracker without Etoposide release compared to DMSO control. Three biological replicates were perfomed. A minimum of 1,000 cells were quantified for each condition, using Harmony. *P*-values were calculated using linear regression on the log2 normalized values and the interaction term *P*-value between PRDX1 background and etoposide treatment is shown in the plot, where ns: not significant ($P > 0.05$), $^{*}P < 0.05$, $^{**}P < 0.01$, $^{***}P < 0.001$, $^{****}P < 0.0001$.

C Validation of shPRDX1 through Immunoblot (top) for PRDX1 and Tubulin on protein extracts from U2-OS shControl and shPRDX1 cell populations and visualization of PRDX1 (in red) in U2-OS shControl and shCOX4 cells (bottom). Images were acquired with the Operetta High Content Screening System in confocal mode, scale bar is 25 μm.

 © 2023 The Authors

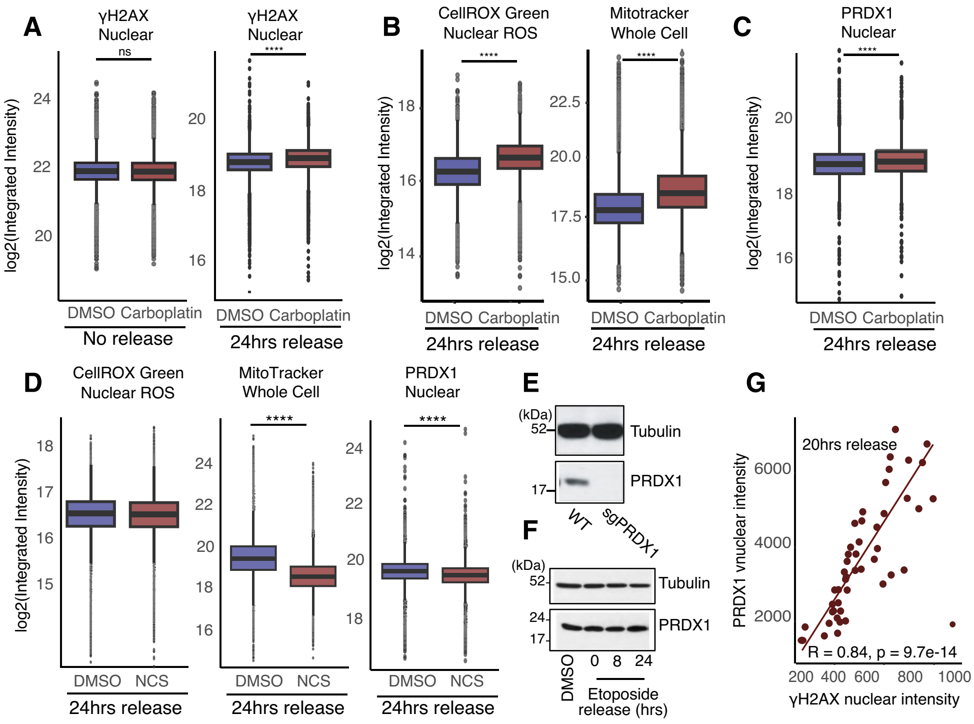

Figure EV5. PRDX1 nuclear abundance is related to DNA damage levels.

A Quantification of nuclear γH2AX integrated intensity without release or 24 h release from Carboplatin compared to DMSO control, in U2-OS WT cells. Three biological replicates were performed. A minimum of 1,000 cells were quantified for each condition, using Harmony. Boxplots represent the median within the IQR. *P*-values were calculated using the Student's *t*-test where ns: not significant ($P > 0.05$), *$P < 0.05$, **$P < 0.01$, ***$P < 0.001$, ****$P < 0.0001$.

B Quantification of nuclear ROS and whole-cell Mitotracker integrated intensity at 24 h release from Carboplatin compared to DMSO control, in U2-OS WT cells. Three biological replicates were performed. A minimum of 1,000 cells were quantified for each condition, using Harmony. Boxplots represent the median within the IQR. *P*-values were calculated using the Student's *t*-test where ns: not significant ($P > 0.05$), *$P < 0.05$, **$P < 0.01$, ***$P < 0.001$, ****$P < 0.0001$.

C Quantification of nuclear PRDX1 integrated intensity at 24 h of release from Carboplatin compared to DMSO control, in U2-OS WT cells. Three biological replicates were performed. A minimum of 1,000 cells were quantified for each condition, using Harmony. Boxplots represent the median within the IQR. *P*-values were calculated using the Student's *t*-test where ns: not significant ($P > 0.05$), *$P < 0.05$, **$P < 0.01$, ***$P < 0.001$, ****$P < 0.0001$.

D Quantification of nuclear ROS, whole-cell Mitotracker, and nuclear PRDX1 integrated intensity at 24 h of release from NCS compared to DMSO control, in U2-OS WT cells. Three biological replicates were performed. A minimum of 1,000 cells were quantified for each condition, using Harmony. Boxplots represent the median within the IQR. *P*-values were calculated using the Student's *t*-test where ns: not significant ($P > 0.05$), *$P < 0.05$, **$P < 0.01$, ***$P < 0.001$, ****$P < 0.0001$.

E Immunoblot for PRDX1 and Tubulin on protein extracts from U2-OS WT and sgPRDX1 cells.

F Immunoblot showing PRDX1 abundance in total extracts of U2-OS cells after etoposide treatment and release in drug-free media. Tubulin is used as a loading control.

G Correlation between γH2AX and PRDX1 nuclear-integrated intensities in U2-OS WT cells at 20 h etoposide release.

© 2023 The Authors

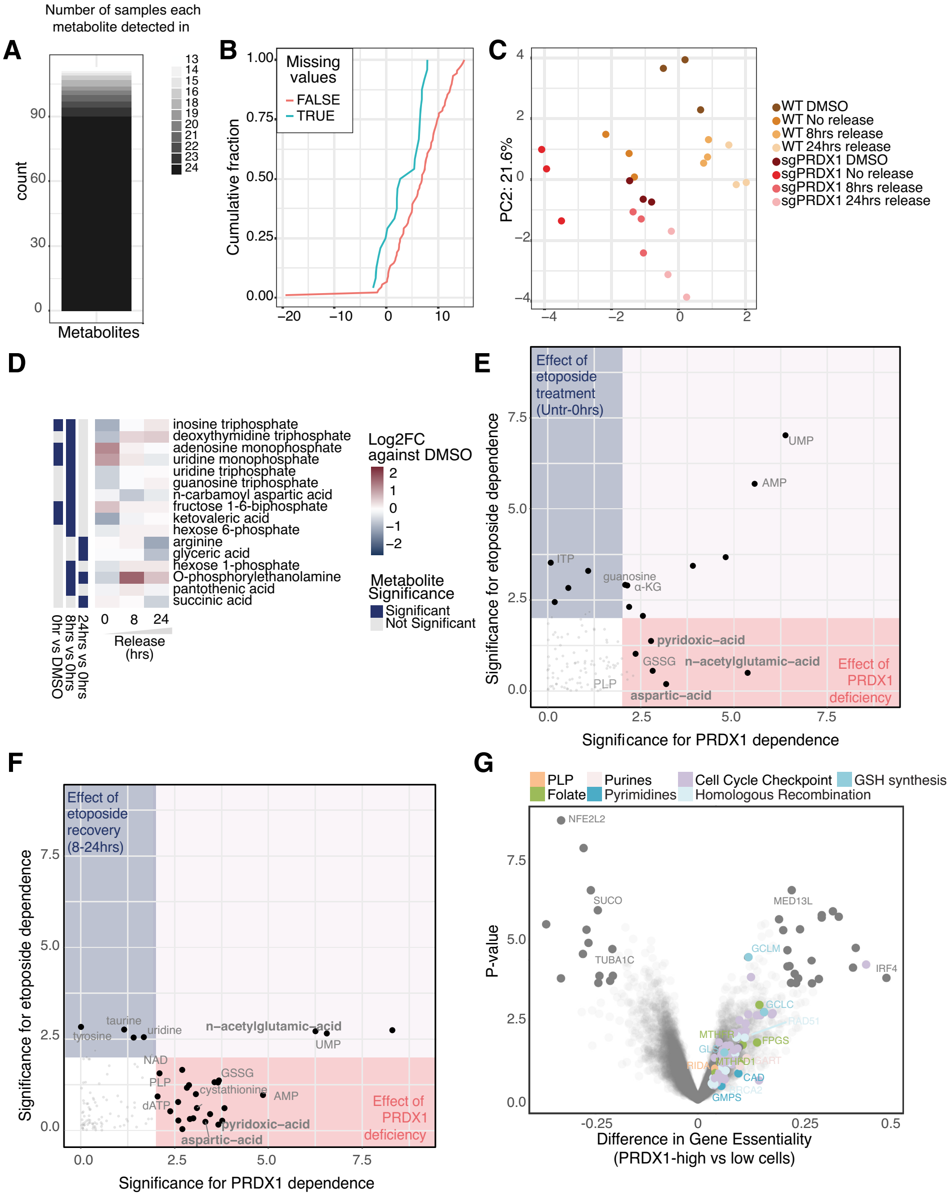

Figure EV6. Etoposide-release metabolomics.

A Number of metabolites detected per sample in the targeted metabolomics experiment.

B Distribution of intensities of consistently detected- and partially detected metabolites showing an intensity-dependent detection pattern.

C PCA plot for all samples in the metabolomics experiment.

D Significantly affected metabolites due to etoposide treatment and 24-h release in U2- OS sgPRDX1 cells.

E, F PRDX1 deficiency-dependency and etoposide treatment-dependency of analyzed metabolites, based on linear regression analysis on the Untreated – 0 h (E) and 8–24 h release timepoints (F).

G Differential gene essentiality between high and low PRDX1-expressing cell lines (CCLE) as in the Achilles dataset. Cell lines with low PRDX1 expression are significantly more sensitive to the depletion of genes represented in the right part of the x-axis as compared to cell lines with high PRDX1 expression.

 © 2023 The Authors

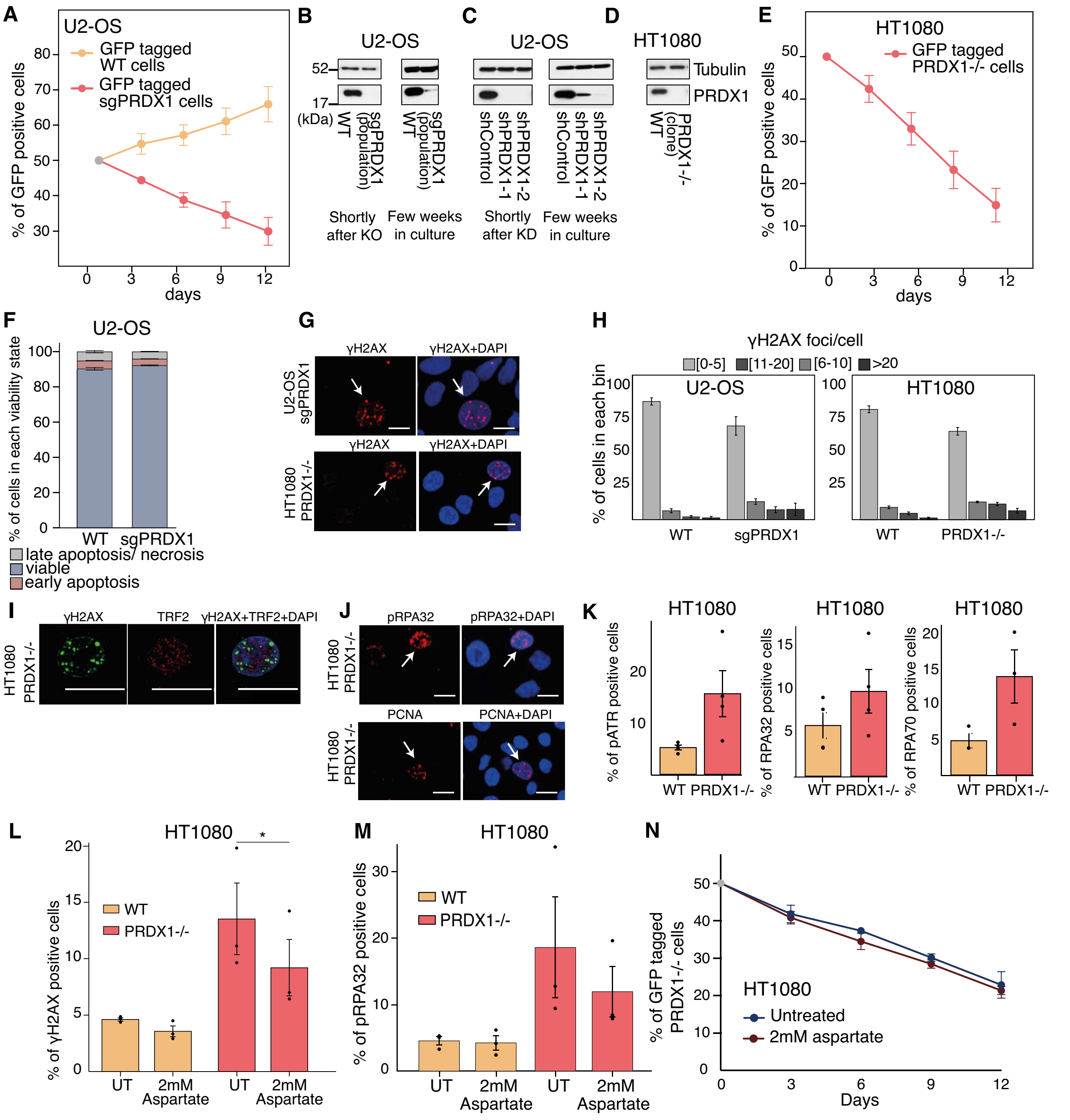
A
U2-OS
GFP tagged WT cells
GFP tagged sgPRDX1 cells
% of GFP positive cells
days
B
U2-OS
52
17
(kDa)
WT
sgPRDX1 (population)
Shortly after KO
Few weeks in culture
C
U2-OS
shControl
shPRDX1-1
shPRDX1-2
Shortly after KD
Few weeks in culture
D
HT1080
Tubulin
PRDX1
WT
PRDX1-/- (clone)
E
HT1080
GFP tagged PRDX1-/- cells
% of GFP positive cells
days
F
U2-OS
% of cells in each viability state
WT
sgPRDX1
late apoptosis/ necrosis
viable
early apoptosis
G
γH2AX
γH2AX+DAPI
U2-OS sgPRDX1
HT1080 PRDX1-/-
H
γH2AX foci/cell
[0-5]
[11-20]
[6-10]
>20
% of cells in each bin
U2-OS
HT1080
WT
sgPRDX1
PRDX1-/-
I
γH2AX
TRF2
γH2AX+TRF2+DAPI
HT1080 PRDX1-/-
J
pRPA32
pRPA32+DAPI
PCNA
PCNA+DAPI
HT1080 PRDX1-/-
K
HT1080
% of pATR positive cells
% of RPA32 positive cells
% of RPA70 positive cells
WT PRDX1-/-
L
HT1080
% of γH2AX positive cells
WT
PRDX1-/-
UT
2mM Aspartate
M
HT1080
% of pRPA32 positive cells
N
HT1080
Untreated
2mM aspartate
% of GFP tagged PRDX1-/- cells
Days

Figure EV7.

Figure EV7. PRDX1 loss generates DNA damage that can be partially rescued by exogenous aspartate supplementation.

A Competitive growth assay of U2-OS WT and sgPRDX1 cells. Samples are normalized to Day 0, data represent the mean and SEM of three independent replicates.
B–D Immunoblot of Tubulin and PRDX1 in U2-OS WT and sgPRDX1 (B), U2-OS shControl and shPRDX1 cell populations (C), and HT1080 WT and PRDX1$^{-/-}$ cells (D).
E Competitive growth assay of HT1080 WT and PRDX1$^{-/-}$ cells. Samples are normalized to Day 0, data represent the mean and SEM of six independent replicates.
F Detection of apoptosis in U2-OS WT and sgPRDX1 cells using Annexin V-Propidium Iodide staining. Data represent the mean and SEM of three independent replicates.
G Visualization of γH2AX (in red) within DAPI stained nuclei (in blue) in U2-OS sgPRDX1 and HT1080 PRDX1$^{-/-}$ cells. Cells positive for γH2AX are indicated with a white arrow. Cells were untreated and images were acquired on an Opera High Content Screening System, the scale bar is 20 μm.
H Quantification of images shown in (G), represented as the percentage of cells in each bin of γH2AX foci number. A minimum of 445 cells were quantified for each condition and replicate, using CellProfiler. Data represent the mean and SEM of five or six independent replicates for U2-OS or HT1080 cells, respectively.
I Visualization of the DNA damage marker γH2AX (in green) and the telomere markers TRF2 in HT1080 PRDX1$^{-/-}$ cells, respectively (in red). Cells were untreated and images were acquired on an Olympus spinning disk confocal microscope, the scale bar is 20 μm.
J Visualization of the replication stress markers pRPA32 and PCNA in HT1080 PRDX1$^{-/-}$ cells. Cells positive for pRPA32 or PCNA are indicated with a white arrow. Cells were untreated and images were acquired on an Opera High Content Screening System, the scale bar is 20 μm.
K Quantification of immunofluorescence images after staining of the replication stress markers RPA32, RPA70, and pATR, in HT1080 WT and PRDX1$^{-/-}$ cells. A minimum of 375 cells for RPA32 staining, 900 cells for RPA70 staining, and 180 cells for pATR staining were quantified for each condition and replicate, using CellProfiler. Data represent mean and SEM of four independent replicates for RPA32 and pATR stainings and three independent replicates for RPA70 staining. *P*-values were calculated using paired *t*-test where ns: not significant ($P > 0.05$), *$P < 0.05$, **$P < 0.01$, ***$P < 0.001$, ****$P < 0.0001$.
L Quantification of percentage γH2AX positive cells in HT1080 WT or PRDX1$^{-/-}$ cells, either untreated (UT) or treated for 3 days with the 2 mM aspartate. Three independent biological replicates are represented. *P*-values were calculated using the Student's *t*-test where ns: not significant ($P > 0.05$), *$P < 0.05$, **$P < 0.01$, ***$P < 0.001$, ****$P < 0.0001$.
M Quantification of the percentage of pRPA32-positive cells in HT1080 WT or PRDX1$^{-/-}$ cells, either untreated (UT) or treated for 3 days with 2 mM aspartate. A minimum of 300 cells were quantified for each condition and replicate, using CellProfiler. Data represent the mean and SEM of three independent replicates. *P*-values were calculated using paired *t*-test where ns: not significant ($P > 0.05$), *$P < 0.05$, **$P < 0.01$, ***$P < 0.001$, ****$P < 0.0001$.
N Competitive growth assay of HT1080 WT and PRDX1$^{-/-}$ cells after 2 mM aspartate treatment. Samples are normalized to Day 0, data represent the mean and SEM of three independent replicates.

© 2023 The Authors
