# Supplementary material for: A metabolic map of the DNA damage response identifies PRDX1 in the control of nuclear ROS scavenging and aspartate availability
Source: Mol Syst Biol. 2023 Jun 1;19(7):e11267. doi: 10.15252/msb.202211267 (PMC10333845; doi:10.15252/msb.202211267)
Supplement: Supplementary file 12 [file MSB-19-e11267-s012.pdf]

molecular systems biology

A metabolic map of the DNA damage response identifies PRDX1 in nuclear ROS scavenging and aspartate availability.

Amandine Moretton, Savvas Kourtis, Antoni Gañez Zapater, Chiara Calabrò, Lorena Espinar, Frederic Fontaine, Evangelia Darai, Etna Abad Cortel, Samuel Block, Laura Pascual, Natalia Pardo Lorente, Ritobrata Ghose, Matthew Vander Heiden, Ana Janic, André Müller, Joanna Loizou, and Sara Sdelci
DOI: 10.15252/msb.202211267

Corresponding author(s): Sara Sdelci (sara.sdelci@crg.eu) , Joanna Loizou (joanna_loizou@hotmail.com)

Review Timeline:

Submission Date:	1st Aug 22
Editorial Decision:	19th Sep 22
Appeal Received:	2nd Dec 22
Editorial Decision:	16th Dec 22
Revision Received:	22nd Mar 23
Editorial Decision:	12th Apr 23
Revision Received:	8th May 23
Accepted:	10th May 23

Editor: Maria Polychronidou

Transaction Report:

(Note: With the exception of the correction of typographical or spelling errors that could be a source of ambiguity, letters and reports are not edited. Depending on transfer agreements, referee reports obtained elsewhere may or may not be included in this compilation. Referee reports are anonymous unless the Referee chooses to sign their reports.)

19th Sep 2022

RE: Manuscript MSB-2022-11267, A metabolic map of the DNA damage response identifies PRDX1 in nuclear ROS scavenging and aspartate synthesis

Dear Dr. Sdelci,

Thank you again for submitting your work to Molecular Systems Biology. I would like to apologize for the delay in sending you a decision, which was due to the late arrival of the referee reports. We have now heard back from two of the three referees who agreed to evaluate your study. Unfortunately, after a series of reminders we did not manage to obtain a report from reviewer #3 and in the interest of time we decided to proceed with making a decision based on the two available reports. As you will see below, the reviewers raise substantial concerns on your work, which unfortunately preclude its publication in Molecular Systems Biology.

Overall, the reviewers appreciate the large amount of data generated in the study. However, they point out that as it stands the study remains rather descriptive and does not offer sufficient novel insights. As such, the reviewers indicated that they do not support publication in Molecular Systems Biology.

Taken together, I am afraid I see no other choice than to return the manuscript with the message that we cannot offer to publish it. I am sorry that the review of your work did not result in a more favorable outcome on this occasion, but I hope that you will not be discouraged from submitting future work to Molecular Systems Biology. In any case, thank you for the opportunity to examine this work.

Yours sincerely,

Maria Polychronidou, PhD
Senior Editor
Molecular Systems Biology

Reviewer #1:

The authors present a high-throughput work on the metabolic changes that take place upon exposure of cells to etoposide. Their analysis shows that PRDX1 is a major DNA damage surveillance factor and that its lack contributes to genome instability.
Major criticism: Overall, the work is of potential interest and the approach is credible and in may ways unique. However, the study lacks meaning and focus as it summarizes a number of different data with no functional follow-up or a clear indication of what the novel aspects of these findings are. For instance, PRDX1 is known to play a role in the DDR (through various pathways). Likewise several of the identified proteins are already known to bind chromatin or have a role in the DNA repair and the DDR.
Minor comments:
1. A clear definition of what the authors mean with the term "metabolic factors" would help the readership understand the rational.
2. ROS are typically generated in the cytoplasm and the mitochondria and are only known to be transiently transferred to the nucleus. Therefore, I fail to see how an increase in ROS levels in the nucleus would imply that metabolic enzymes are associated with chromatin.
3. Etoposide forms covalent TOP2-DNA cleavage complexes affecting transcription, DNA replication and triggering some types of DNA damage. It may be an over-interpretation to define the response of cells to etoposide as "the DNA damage response".
4. ENO1 is known to bind to chromatin and to act as a transcription repressor. The role of PRDX1 on DDR is also well established for telomeric DNA damage and in prdx1 KO mice. The authors may wish to re-phrase or else highlight the novelty of their findings in view of what has already been published.
5. Lower aspartate-dependent metabolites in PRDX1 could be a indirect contributing factor to genome instability but the specificity of aspartate synthesis to PRDX1 per se upon DNA damage is not clearly shown.

Reviewer #3:

In this manuscript, using elegant CRISPR screens focusing on metabolic enzymes, a joint effort between the Loizou and Sdelci

labs reveals previously unidentified role for the oxidoreductase peroxiredoxin 1 (PRDX1) in scavenging nuclear ROS following DNA damage and aspartate synthesis.

The authors highlight some interesting features on several metabolic proteins and metabolites suggesting conceptually novel and interesting links to the DNA damage response worth investigating, but there is only little experimental evidence presented beyond the screens, with the notable exception of PRDX1. Thus, screen and metabolomics data are comprehensive and will provide a suitable resource for the field. Yet, the manuscript falls in my opinion short in firmly establishing a prominent role for PRDX1 in response to (exogenous) DNA damage.

• Different from what the microscopic images are indicating (Figure 1C), there is quite a high level of mitochondrial and nuclear (physiological) ROS in untreated cells, that is, on average, almost indistinguishable from those of Etoposide treated cells (Figure 1D), even though significant p-values seemingly claim otherwise. Can the authors comment on that? It would be interesting to see whether co-treatment of cells with antioxidants (e.g., ascorbic acid) would lead to a restoration of mitochondrial ROS after etoposide.
• To corroborate the phenotypes reported for PRDX1 depletion, I would strongly recommend the authors monitoring both etoposide-induced nuclear ROS and gH2AX levels and metabolites in cells inducibly-expressing PRDX1.
• I cannot really follow the authors reasoning of focusing on PRDX1 for their follow-up studies, as its increase on chromatin abundance following etoposide treatment is not statistically significant. Along the same lines, PRDX1 depletion (using shRNA) in untreated cells already results in an increase in ROS (and gH2X foci), comparable to that observed upon etoposide (Figures 3A and 3B). Consistent with this, and as shown in Figure 3C, PRDX1 does not form foci in response to DSB induction and accumulate in the nucleus at a timepoint where gH2AX have disappeared (Figure 3C). I do not understand how the authors see a clear correlation between PRDX1 and DNA damage levels, as stated on p. 8. In fact, there is a much stronger contribution of PRDX1 in scavenging ROS and influencing the metabolic state of cells in absence of exogenous DNA damage. Experiments that could help solving this issues: PRDX1-depleted cells hypersensitive to etoposide treatment (probably not given that PRDX1 is not SL with etoposide), or, vice versa, are PRDX1-overexpressing cells more resistant to DNA damage treatment?
• Establishing a pivotal role of PRDX1 in alleviating ROS-induced replication stress, I recommend the authors to examine replication fork phenotypes (e.g. stalling, reversal/restart, nascent strand) degradation) in unstressed cells and in response to DNA damage using classical DNA fiber spreading protocols.

Minor comments:
• Please describe in the materials section the CRISRP-Cas9 library (how many genes, how many sgRNAs/per gene)
• p.3: H2AX phosphorylation can no longer be considered as a (specific) DSB marker but rather as a general marker of DNA damage.
• In Figures 2B and 2C, please indicate in the volcano plots the threshold for 'significant hits.
• A proteomics approach is taken to investigate which metabolic enzymes are becoming enriched at damage chromatin. The analysis apparently revealed 11 metabolic enzymes that were significantly altered (enriched or depleted on chromatin, immediately after (no release) or 24h-post etoposide (Table S1). However, Table S1 does not list which of those 11 proteins belongs to which of the four different 'categories. Moreover, Rad18 and Bard1 are also included in that list (how comes?). I think there is in general quite some confusions in the text and/or in the Legend on what is displayed on Table S1 ("Gene lists belonging in each of the categories of S2F" or "11 hits"?) and Table S2.

** As a service to authors, EMBO Press offers the possibility to directly transfer declined manuscripts to another EMBO Press title or to the open access journal Life Science Alliance launched in partnership between EMBO Press, Rockefeller University Press and Cold Spring Harbor Laboratory Press. The full manuscript and if applicable, reviewers' reports, are automatically sent to the receiving journal to allow for fast handling and a prompt decision on your manuscript. For more details of this service, and to transfer your manuscript please click on Link Not Available. **

A metabolic map of the DNA damage response identifies PRDX1 in nuclear ROS scavenging and aspartate availability

Response to the Reviewers :

We thank the reviewers for stating that our work is of high quality, elegant, and unique. We appreciate it.

We are very grateful also for the criticisms, which allowed us to give our manuscript a different, more concrete, shape and to add experiments that we believe made the story worth being considered for publication in Molecular System Biology.

The major changes introduced in the manuscript in the past 2 months are the following:

1. As a follow-up of our survival screening we now show that the inhibition of ETC enzymes improves the survival of U2-OS cells treated with etoposide (Figure 1F), thus corroborating the survival screening outcome.

2. We state more clearly that the role of PRDX1 in the DNA damage response that we describe is independent of the role already described at telomeres (10.1016/j.celrep.2016.11.071) (Figure 3C and Supplementary Figure 5I). While we acknowledge these data referencing such discovery, we believe that the role of PRDX1 in the DNA damage response has not been exhaustively described and that our data provide a link between PRDX1-driven ROS scavenging and metabolism rewiring during DNA damage.

3. In this new version of the manuscript, we explain in more details why we decided to follow up PRDX1. Briefly, our decision is mainly based on the fact that despite we did not see a significant PRDX1 chromatin increase in our chromatin proteomics experiment (only a trend, Supplementary Figure 2I), we do see a clear PRDX1 nuclear increase in cells perturbed with etoposide (Figure 3E and 3F). This indicates that we are observing an etoposide-driven nuclear translocation rather than chromatin-specific relocalization.

4. Supporting this hypothesis, we now show that cells accumulate PRDX1 in the nucleus in G2 even in absence of DNA damage, using a customized FUCCI system. Interestingly, this observation is consistent with the proposed role of PRDX1 scavenging nuclear ROS given the fact that during G2/M there is an increased accumulation of cellular - and possibly nuclear - ROS (10.1016/j.cels.2019.01.005, 10.1016/j.devcel.2022.06.008) (Figure 3H and Supplementary Figure 3I-K).

5. Additionally, we show now that cells downregulated for PRDX1 are significantly more sensitive to etoposide treatment (Figure 3B), confirming our hypothesis of PRDX1 being required during DNA damage repair.

6. Further, we deeper analyzed our metabolomics data, which interestingly indicated that DNA damage induced *de novo* nucleotide synthesis (already mentioned in the previous version of the manuscript). However, in the absence of PRDX1, the aspartate required for nucleotide synthesis is directed towards GSH synthesis due to GSH over-oxidation into GSSG, making *de novo* nucleotide synthesis not possible and impairing DNA damage repair (Figure 3I-F and Supplementary Figure 4E-F). Thus, we now changed the title as follows:

A metabolic map of the DNA damage response identifies PRDX1 in nuclear ROS scavenging and aspartate **availability**

7. Finally, essentiality data (https://depmap.org/portal/achilles/) extracted from PRDX1 high and low-expressing cells (CCLE) corroborated our metabolomics experiment. Indeed, in low-expressing PRDX1 cells, genes related to nucleotide metabolism and GSH biosynthesis are much more essential, together with genes of DNA repair, especially homologous recombination and cell cycle checkpoints (Supplementary Figure 4G).

Reviewer #1:

The authors present a high-throughput work on the metabolic changes that take place upon exposure of cells to etoposide. Their analysis shows that PRDX1 is a major DNA damage surveillance factor and that its lack contributes to genome instability.

Major criticism: Overall, the work is of potential interest and the approach is credible and in many ways unique.

We thank the reviewer for these positive words.

However, the study lacks meaning and focus as it summarizes a number of different data with no functional follow-up or a clear indication of what the novel aspects of these findings are. For instance, PRDX1 is known to play a role in the DDR (through various pathways).

As explained before, we now explain that the role of PRDX1 in the DNA damage response that we describe is different from the one observed at telomeric regions (10.1016/j.celrep.2016.11.071) (Figure 3C and Supplementary Figure 5I). Moreover, the role of PRDX1 in aspartate availability has not been reported before. We hope that our explanation and the additional results included in the new version of our manuscript will convince the reviewer about the novelty of our work.

Likewise several of the identified proteins are already known to bind chromatin or have a role in the DNA repair and the DDR.

We thank the reviewer for this observation. Indeed, those factors are important controls that highlight the high quality and trustworthiness of our multiple datasets. Each time, we mention the relevant literature to indicate that these proteins have been identified in previous work.

Minor comments:

1. A clear definition of what the authors mean with the term "metabolic factors" would help the readership understand the rational.

We thank the reviewer for this observation. We have now clarified it in the text. Briefly, metabolic factors are retrieved from the metabolic CRISPR library and the MetabolicAtlas (https://metabolicatlas.org), which includes not only strict metabolic enzymes but also regulators of metabolism (e.g. transcription factors known to be involved in the activation of metabolic pathways).

2. ROS are typically generated in the cytoplasm and the mitochondria and are only known to be transiently transferred to the nucleus. Therefore, I fail to see how an increase in ROS levels in the nucleus would imply that metabolic enzymes are associated with chromatin.

We apologize for jumping to conclusions. It is very exciting for us to see that enzymes of the ETC localize on chromatin during DNA damage, but we certainly do not have any proof of causality, and this could be a consequence of some sort of mitochondrial dysfunction caused by etoposide treatment that allows ETC enzymes leak from the mitochondria. We are currently performing immunofluorescence targeting different ETC-proteins to see whether upon etoposide treatment we observe their nuclear translocation. This type of experiment requires time since we will need to validate each result with the downregulation of the respective ETC factor to exclude antibody unspecific binding. We are more than happy to include this experiment in the revised version of our manuscript. Additionally, we are planning to treat the cells with different DNA damage inducers to understand whether the effect is due to the etoposide treatment (which is known to increase mitochondrial mass https://doi.org/10.1038/srep34064), or due to the accumulation of DNA damage. We believe that these experiments can be completed in the next 3-4 months.

3. Etoposide forms covalent TOP2-DNA cleavage complexes affecting transcription, DNA replication and triggering some types of DNA damage. It may be an over-interpretation to define the response of cells to etoposide as "the DNA damage response".

We thank the reviewer for this observation. Even though etoposide has been classically used as a double strand break inducer, we agree that it provokes effects that are beyond DNA damage. As explained in the previous point, we are planning to use different DNA damage inducers. Therefore, we will recapitulate the key findings of our manuscript (nuclear ROS increase, PRDX1 nuclear translocation, Aspartate rescue) using those inhibitors. We expect to have these experiments concluded in the next 3-4 months.

4. ENO1 is known to bind to chromatin and to act as a transcription repressor. The role of PRDX1 on DDR is also well established for telomeric DNA damage and in prdx1 KO mice. The authors may wish to re-phrase or else highlight the novelty of their findings in view of what has already been published.

We thank the reviewer for the suggestion. While we do not follow up on ENO1, we clearly show that we do not observe colocalization of DNA damage and telomere markers in the 2 different PRDX1 KO cell lines used (Figure 3C and Supplementary Figure 5I), indicating an independent role of PRDX1 in DNA damage beyond telomeres, at least in our systems. Using PRDX1 KO mice, PRDX1 was shown to be involved in Pten-mediated tumour suppression in Ras-induced breast cancer. The role of PRDX1 in the breakdown of intracellular aspartate levels between GSH synthesis and *de novo* nucleotide metabolism, which we uncover in this study, is entirely novel. This equilibrium gets compromised during the DNA damage response due to the increased ROS levels, thus making PRDX1 an important protein in DNA repair. Additionally, the nuclear relocalisation of PRDX1 following DNA damage has not been shown before.

C/ Dr. Aiguader 88, 08003 Barcelona +34 316 01 00 sara.sdelci@crg.eu www.crg.es
Lazarettgasse 14, AKH BT 25.3, 1090 Vienna, Austria, Phone +43-1/40160-70058, joanna_loizou@hotmail.com www.cemm.at

5. Lower aspartate-dependent metabolites in PRDX1 could be a indirect contributing factor to genome instability but the specificity of aspartate synthesis to PRDX1 per se upon DNA damage is not clearly shown.

We thank the reviewer for this observation. As explained before, by digging into our metabolomics experiment we observed that in the absence of PRDX1, the aspartate required for nucleotide synthesis is directed towards GSH synthesis due to GSH over-oxidation into GSSG, making *de novo* nucleotide synthesis not possible and impairing DNA damage repair (Figure 3I-F and Supplementary Figure 4E-F).

Moreover, essentiality data (https://depmap.org/portal/achilles/) extracted from PRDX1 high and low-expressing cells (CCLE) corroborated our metabolomics experiment. Indeed, in low-expressing PRDX1 cells, genes related to nucleotide metabolism and GSH biosynthesis are much more essential, together with genes of DNA repair, especially homologous recombination and cell cycle checkpoints (Supplementary Figure 4G).

These new data link PRDX1 to aspartate availability towards *de novo* nucleotide synthesis. To further confirm this evidence, we are now inducing DNA damage in PRDX1 KO cells supplemented with aspartate (already in the manuscript), nucleosides/nucleotides and glutamine either alone or in combination with antioxidants (e.g., ascorbic acid) to see whether such treatments can rescue the DNA damage accumulation and the proliferation defect of those cells. We expect to have these experiments concluded in the next 3-4 months.

Reviewer #3:

In this manuscript, using elegant CRISPR screens focusing on metabolic enzymes, a joint effort between the Loizou and Sdelci labs reveals previously unidentified role for the oxidoreductase peroxiredoxin 1 (PRDX1) in scavenging nuclear ROS following DNA damage and aspartate synthesis.

We thank the reviewer for this positive comment.

The authors highlight some interesting features on several metabolic proteins and metabolites suggesting conceptually novel and interesting links to the DNA damage response worth investigating, but there is only little experimental evidence presented beyond the screens, with the notable exception of PRDX1. Thus, screen and metabolomics data are comprehensive and will provide a suitable resource for the field. Yet, the manuscript falls in my opinion short in firmly establishing a prominent role for PRDX1 in response to (exogenous) DNA damage.

We hope that the new results will convince the reviewer about the fact that the presence of PRDX1 is essential for DNA damage repair.

• Different from what the microscopic images are indicating (Figure 1C), there is quite a high level of mitochondrial and nuclear (physiological) ROS in untreated cells, that is, on average, almost indistinguishable from those of Etoposide treated cells (Figure 1D), even though significant p-values seemingly claim otherwise. Can the authors comment on that? It's true that in 1D it looks like ROS

levels are very close even if significantly lower for DMSO, while on the pictures in 1C it does look really lower, especially for mito ROS.

We thank the reviewer for this interesting comment. Statistical significance in experiments with numerous observations is achieved even for small differences if those are constant, which should be interpreted as a clear tendency for the two populations to differ from each other. In the commented experiment (as well as in the majority) we analyzed more than 500 cells in each condition, giving statistical power to detect small deviations from the control condition (DMSO). The images shown are meant to facilitate the observations we make about increased ROS 24hr after Etoposide, and they are accompanied by the quantification to show that the changes we observe are not dramatic, and certainly more pronounced in the nucleus, but significant.

It would be interesting to see whether co-treatment of cells with antioxidants (e.g., ascorbic acid) would lead to a restoration of mitochondrial ROS after etoposide.

We thank the reviewer for this suggestion. By digging into our metabolomics experiment we observed that in the absence of PRDX1, the aspartate required for nucleotide synthesis is directed towards GSH synthesis due to GSH over-oxidation into GSSG, making *de novo* nucleotide synthesis not possible and impairing DNA damage repair Figure 3I-F and Supplementary Figure 4E-F)

Moreover, essentiality data (https://depmap.org/portal/achilles/) extracted from PRDX1 high and low-expressing cells (CCLE) corroborated our metabolomics experiment. Indeed, in low-expressing PRDX1 cells, genes related to nucleotide metabolism and GSH biosynthesis are much more essential, together with genes of DNA repair, especially homologous recombination and cell cycle checkpoints (Supplementary Figure 4G).

These new data link PRDX1 to aspartate availability towards *de novo* nucleotide synthesis. To further confirm this evidence, we are now inducing DNA damage in PRDX1 KO cells supplemented with aspartate (already in the manuscript), nucleosides/nucleotides and glutamine either alone or in combination with antioxidants (e.g., ascorbic acid) to see whether such treatments can rescue the DNA damage accumulation and the proliferation defect of those cells. We expect to have these experiments concluded in the next 3-4 months.

• To corroborate the phenotypes reported for PRDX1 depletion, I would strongly recommend the authors monitoring both etoposide-induced nuclear ROS and gH2AX levels and metabolites in cells inducibly-expressing PRDX1.

We thank the reviewer for this suggestion. We are in the process of producing the cell line. We, however, tried already to overexpress PRDX1 with small success given the fact that the level of overexpression was minimal. We expect to have these experiments concluded in the next 3-4 months.

• I cannot really follow the authors reasoning of focusing on PRDX1 for their follow-up studies, as its increase on chromatin abundance following etoposide treatment is not statistically significant. Better explained. Along the same lines, PRDX1 depletion (using shRNA) in untreated cells already results in an increase in ROS (and gH2X foci), comparable to that observed upon etoposide (Figures 3A and 3B). Consistent with this, and as shown in Figure 3C, PRDX1 does not form foci in response to DSB induction and accumulate in the nucleus at a timepoint where gH2AX have disappeared (Figure 3C). I do not understand how the authors see a clear correlation between PRDX1 and DNA

C/ Dr. Aiguader 88, 08003 Barcelona +34 316 01 00 sara.sdelci@crg.eu www.crg.es
Lazarettgasse 14, AKH BT 25.3, 1090 Vienna, Austria, Phone +43-1/40160-70058, joanna_loizou@hotmail.com www.cemm.at

damage levels, as stated on p. 8. In fact, there is a much stronger contribution of PRDX1 in scavenging ROS and influencing the metabolic state of cells in absence of exogenous DNA damage.

We thank the reviewer for this criticism that made us brainstorm about our data quite a bit. We believe that the fact that PRDX1 accumulates in the nucleus when gH2AX has disappeared does not mean that PRDX1 does not play a role in the DNA damage response. Our experiments, both the chromatin proteomics and the metabolomics, show that despite the lack of gH2AX 24 hours after etoposide release, the DNA damage response is not yet over and the cells did not recover their basal state. In agreement with this, nuclear ROS levels reach a maximum at 24 hours after etoposide release, when PRDX1 is also recruited in the nucleus even though gH2AX is already gone.

It is well reported that PRDX1 has a role in scavenging ROS even in absence of DNA damage, but the fact that it localizes to the nucleus during the DNA damage response to scavenge etoposide-induced ROS indicates that this protein has a clear purpose in the DNA damage response. It is, therefore, expected that the lack of PRDX1 would lead, as we observe, to DNA damage accumulation in replicating cells, which constantly have to deal with some *physiological* DNA damage that might be not cleared properly in absence of PRDX1.

Experiments that could help solving this issues: PRDX1-depleted cells hypersensitive to etoposide treatment (probably not given that PRDX1 is not SL with etoposide), or, vice versa, are PRDX1-overexpressing cells more resistant to DNA damage treatment?

As explained before, the overexpression of PRDX1 did not result in an evident increase of the protein level, therefore results from the etoposide treatment were not conclusive, however, they were showing the expected trend (PRDX1 overexpression confers resistance to etoposide treatment). We are now trying to improve the overexpression of PRDX1 (maybe by changing the tagging, the one we used was Maroon) to repeat the experiment. We expect to have these experiments concluded in the next 3-4 months.

Moreover, we performed etoposide treatment on U2-OS PRDX1 depleted cells and we observed that they are significantly more sensitive to the treatment when compared to shControl cells (Figure 3B).

• Establishing a pivotal role of PRDX1 in alleviating ROS-induced replication stress, I recommend the authors to examine replication fork phenotypes (e.g. stalling, reversal/restart, nascent strand) degradation) in unstressed cells and in response to DNA damage using classical DNA fiber spreading protocols.

We acknowledge that DNA fiber spreading assay could give additional information about the effect of PRDX1 depletion directly on the replication fork. However, this goes beyond the scope of our study as our first aim is to emphasize the crosstalk between metabolism and the DNA damage response, for which we have used a variety of global and unbiased approaches.

Minor comments:

• Please describe in the materials section the CRISRP-Cas9 library (how many genes, how many sgRNAs/per gene).

We thank the reviewer for this observation, and we have now included this information.

C/ Dr. Aiguader 88, 08003 Barcelona +34 316 01 00 sara.sdelci@crg.eu www.crg.es
Lazarettgasse 14, AKH BT 25.3, 1090 Vienna, Austria, Phone +43-1/40160-70058, joanna_loizou@hotmail.com www.cemm.at

• p.3: H2AX phosphorylation can no longer be considered as a (specific) DSB marker but rather as a general marker of DNA damage.

We thank the reviewer for this observation, and we have now changed it in the text.

• In Figures 2B and 2C, please indicate in the volcano plots the threshold for 'significant hits. Statistically significant protein hits are those with p.adj values less than 0.05. We now specified it in the figure legend for clarity.

• A proteomics approach is taken to investigate which metabolic enzymes are becoming enriched at damage chromatin. The analysis apparently revealed 11 metabolic enzymes that were significantly altered (enriched or depleted on chromatin, immediately after (no release) or 24h-post etoposide (Table S1). However, Table S1 does not list which of those 11 proteins belongs to which of the four different 'categories. Moreover, Rad18 and Bard1 are also included in that list (how comes?)

We have now clarified it in the text. Briefly, metabolic factors are retrieved from the CRISPR library and the MetabolicAtlas (https://metabolicatlas.org), which includes not only strict metabolic enzymes but also metabolism regulators (e.g. transcription factors known to be involved in the activation of metabolic pathways).

I think there is in general quite some confusions in the text and/or in the Legend on what is displayed on Table S1 ("Gene lists belonging in each of the categories of S2F" or "11 hits"?)

We apologize for the confusion. For further clarity, the specific sheet of the Table S1 that shows the 11 metabolic enzymes significantly altered on chromatin is now referenced, and additional information regarding these proteins has been added in the table: “Among the significantly altered proteins, 11 were metabolic enzymes (Table S1 'chromatindiff_Metabolic' sheet).

C/ Dr. Aiguader 88, 08003 Barcelona +34 316 01 00 sara.sdelci@crg.eu www.crg.es
Lazarettgasse 14, AKH BT 25.3, 1090 Vienna, Austria, Phone +43-1/40160-70058, joanna_loizou@hotmail.com www.cemm.at

16th Dec 2022

Manuscript Number: MSB-2022-11267R-Q, A metabolic map of the DNA damage response identifies PRDX1 in nuclear ROS scavenging and aspartate availability

Dear Sara,

Thank you for your message related to our decision on your manuscript MSB-2022-11267. I apologize for the delay in getting back to you, which was due to my absence from the office (as I mentioned, I was attending a conference) in combination with the large number of submissions that we have been receiving before the upcoming holidays. I have now had the chance to read the manuscript and your point-by-point response to the reviewers' comments and I have discussed them with the team. As I will explain below, we would not be opposed to considering a revised and extended manuscript addressing the issues raised by the reviewers.

During the review of the initial study, the two reviewers appreciated the large amount of data generated in the study and thought that the presented findings seem potentially interesting. However, they indicated that the study remained rather descriptive and did not offer sufficiently conclusive novel insights. We think that the proposed additional analyses, as well as the follow up analyses and validations that have already been added to the manuscript, seem potentially promising for addressing the reviewers' concerns and for increasing the conclusiveness and the level of insight. As such, we would invite you to submit a revised manuscript, which will be sent back to the reviewers so that they can assess if their concerns have been satisfactorily addressed. Since, as you mention in your letter, the outcome of some ongoing analyses is unclear (e.g. examining the effect of etoposide treatment on ETC proteins by immunofluorescence, the analyses of additional DNA damage inducers beyond etoposide and the analyses upon PRDX1 overexpression) we would ask you to resubmit after these analyses have been performed and when the results can be included in the manuscript. This will allow the referees to get a complete picture of the validations etc. performed.

The revised manuscript will be subjected to peer review and as you probably understand, given that addressing the issues raised involves analyses with unclear outcome, we can give no guarantee about the eventual acceptability of the study. If you do decide to resubmit the extended/revised manuscript, we would ask you to enclose with your resubmission a point-by-point response to the points raised.

I apologise once again for the slow response and I hope that the comments above will be helpful in deciding how to further proceed with this work.

Kind regards,

Maria

Maria Polychronidou, PhD
Senior Editor
Molecular Systems Biology

We realize that it is difficult to revise to a specific deadline. In the interest of protecting the conceptual advance provided by the work, we recommend a revision within 3 months (16th Mar 2023). Please discuss the revision progress ahead of this time with the editor if you require more time to complete the revisions. Use the link below to submit your revision:

https://msb.msubmit.net/cgi-bin/main.plex

IMPORTANT: When you send your revision, we will require the following items:
1. the manuscript text in LaTeX, RTF or MS Word format
2. a letter with a detailed description of the changes made in response to the referees. Please specify clearly the exact places in the text (pages and paragraphs) where each change has been made in response to each specific comment given
3. three to four 'bullet points' highlighting the main findings of your study
4. a short 'blurb' text summarizing in two sentences the study (max. 250 characters)
5. a 'thumbnail image' (550px width and max 400px height, Illustrator, PowerPoint or jpeg format), which can be used as 'visual title' for the synopsis section of your paper.
6. Please include an author contributions statement after the Acknowledgements section (see https://www.embopress.org/page/journal/17444292/authorguide)
7. Please complete the CHECKLIST available at (https://bit.ly/EMBOPressAuthorChecklist).

Please note that the Author Checklist will be published alongside the paper as part of the transparent process (https://www.embopress.org/page/journal/17444292/authorguide#transparentprocess).
8. When assembling figures, please refer to our figure preparation guideline in order to ensure proper formatting and readability in print as well as on screen:
https://bit.ly/EMBOPressFigurePreparationGuideline
See also figure legend guidelines: https://www.embopress.org/page/journal/17444292/authorguide#figureformat
9. Please note that corresponding authors are required to supply an ORCID ID for their name upon submission of a revised manuscript (EMBO Press signed a joint statement to encourage ORCID adoption).
(https://www.embopress.org/page/journal/17444292/authorguide#editorialprocess)
Currently, our records indicate that the ORCID for your account is 0000-0003-1330-4364.

Please click the link below to modify this ORCID:
Link Not Available

10. At EMBO Press we ask authors to provide source data for the main manuscript figures. Our source data coordinator will contact you to discuss which figure panels we would need source data for and will also provide you with helpful tips on how to upload and organize the files.

The system will prompt you to fill in your funding and payment information. This will allow Wiley to send you a quote for the article processing charge (APC) in case of acceptance. This quote takes into account any reduction or fee waivers that you may be eligible for. Authors do not need to pay any fees before their manuscript is accepted and transferred to the publisher.

EMBO Press participates in many Publish and Read agreements that allow authors to publish Open Access with reduced/no publication charges. Check your eligibility: https://authorservices.wiley.com/author-resources/Journal-Authors/open-access/affiliation-policies-payments/index.html

As a matter of course, please make sure that you have correctly followed the instructions for authors as given on the submission website.

*** PLEASE NOTE *** As part of the EMBO Press transparent editorial process initiative (see our Editorial at https://dx.doi.org/10.1038/msb.2010.72), Molecular Systems Biology publishes online a Review Process File with each accepted manuscripts. This file will be published in conjunction with your paper and will include the anonymous referee reports, your point-by-point response and all pertinent correspondence relating to the manuscript. If you do NOT want this File to be published, please inform the editorial office at msb@embo.org within 14 days upon receipt of the present letter.

CRG Centre for Genomic Regulation

Ce—M—M— Research Center for Molecular Medicine of the Austrian Academy of Sciences

ÖAW

22.03.2023

A metabolic map of the DNA damage response identifies PRDX1 in the control of nuclear ROS scavenging and aspartate availability

Response to the Reviewers :

We thank the reviewers for stating that our work is of high quality, elegant, and unique. We appreciate it.

We are very grateful also for the criticisms, which allowed us to give our manuscript a different, more concrete, shape and to add experiments that we believe made the story worth being considered for publication in Molecular System Biology.

The major changes introduced in the manuscript are the following:

1. As a follow-up of our survival screening we now show that the inhibition of ETC enzymes improves the survival of U2-OS cells treated with etoposide (Figure S1I), thus corroborating the survival screening outcome.

2. To validate the results of chromatome proteomics, we performed confocal immunofluorescence of COX4 and discovered that COX4 can localize in the cellular nucleus. Quantification in the presence and absence of etoposide showed that COX4 increases in the nucleus 24 hours after etoposide release, in concomitance with nuclear ROS increase. Moreover, we show that the downregulation of PRDX1, which induces nuclear ROS increase, also triggers nuclear COX4 accumulation (Figure 3, S3, and S4).

2. We state more clearly that the role of PRDX1 in the DNA damage response that we show is independent of the role already described at telomeres (10.1016/j.celrep.2016.11.071) (Figure 4C-D and S7I). While we acknowledge these data referencing such discovery, we believe that the role of PRDX1 in the DNA damage response has not been exhaustively described and that our data provide a link between PRDX1-driven nuclear ROS scavenging and metabolism rewiring during DNA damage.

3. We, therefore, explain in more detail why we decided to follow up on PRDX1. Briefly, our decision is mainly based on the fact that despite we did not see a significant PRDX1 chromatin increase in our chromatin proteomics experiment (but a trend, Figure S2I), we do see a clear PRDX1 nuclear increase in cells perturbed with etoposide after 24 hours release (Figure 4E-F). These data show that upon DNA damage PRDX1 translocates in the nucleus rather than specifically associate with chromatin.

4. We observed that the nuclear translocation of PRDX1 happens in concomitance with nuclear ROS and COX4 increase also in carboplatin-treated cells. On the contrary, PRDX1 nuclear increase does not occur in NCS-treated cells since the treatment does not induce nuclear ROS increase at the tested time and concentration (Figure S5A-D).

5. Supporting this hypothesis, we now show that cells accumulate PRDX1 in the nucleus in G2 even in absence of DNA damage, using a customized FUCCI system (Figure 4G-J). Interestingly, this observation is consistent with the proposed role of PRDX1 scavenging nuclear ROS given the fact that during G2/M there is an increased accumulation of ROS (10.1016/j.cels.2019.01.005, 10.1016/j.devcel.2022.06.008).

6. Additionally, we show now that cells downregulated for PRDX1 are significantly more sensitive to etoposide and carboplatin treatment (Figure 4B).

7. Furthermore, we analyzed the cell cycle dynamics of cells depleted for PRDX1 and show that they have a G1 delay, which impairs the recovery upon etoposide treatment (Figure 6A-F). When performing a DNA replication fiber assay, we could observe that PRDX1-depleted cells are showing much shorter fibers upon etoposide treatment, supporting their reduced capability of recovery and corroborating their replicative stress phenotype (Figure 6G).

8. Further, we deeper analyzed our metabolomics data, which interestingly indicated that DNA damage induced *de novo* nucleotide synthesis (already mentioned in the previous version of the manuscript). However, in the absence of PRDX1, the aspartate required for nucleotide synthesis is directed towards GSH synthesis due to GSH over-oxidation into GSSG, making *de novo* nucleotide synthesis not possible and impairing DNA damage repair (Figure 5E-M and Figure S6E-F).

Thus, we now changed the title as follows:

A metabolic map of the DNA damage response identifies PRDX1 in **the control of** nuclear ROS scavenging and aspartate **availability**

9. Essentiality data (https://depmap.org/portal/achilles/) extracted from PRDX1 high and low-expressing cells (CCLE) corroborated our metabolomics experiment. Indeed, in low-expressing PRDX1 cells, genes related to nucleotide metabolism and GSH biosynthesis are much more essential, together with genes of DNA repair, especially homologous recombination and cell cycle checkpoints (Figure S6G).

10. Finally, we performed metabolite treatment and showed that ascorbic acid and aspartate decrease the basal amount of DNA damage observed in cells either PRDX1-depleted (Figure 6J) or -deficient (Figure S7L-M). Additionally, we showed that if PRDX1-depleted cells are treated with nucleotides prior to etoposide, they better recover from the DNA damage, conversely to what was observed for the control population (Figure 6K).

Reviewer #1:

The authors present a high-throughput work on the metabolic changes that take place upon exposure of cells to etoposide. Their analysis shows that PRDX1 is a major DNA damage surveillance factor and that its lack contributes to genome instability.

Major criticism: Overall, the work is of potential interest and the approach is credible and in many ways unique.

We thank the reviewer for these positive words.

However, the study lacks meaning and focus as it summarizes a number of different data with no functional follow-up or a clear indication of what the novel aspects of these findings are. For instance, PRDX1 is known to play a role in the DDR (through various pathways).

As explained above, we now explain that the role of PRDX1 in the DNA damage response that we describe is different from the one observed at telomeric regions (10.1016/j.celrep.2016.11.071) (Figure 4C-D and S5I). Moreover, the role of PRDX1 in aspartate availability has not been reported before. We hope that our explanation and the additional results included in the new version of our manuscript will convince the reviewer about the novelty of our work.

Likewise several of the identified proteins are already known to bind chromatin or have a role in the DNA repair and the DDR.

We thank the reviewer for this observation. Indeed, those factors are important controls that highlight the high quality and trustworthiness of our multiple datasets. Each time, we mention the relevant literature to indicate that these proteins have been identified in previous work.

Minor comments:

1. A clear definition of what the authors mean with the term "metabolic factors" would help the readership understand the rational.

We thank the reviewer for this observation. We have now clarified it in the text. Briefly, metabolic factors are retrieved from the metabolic CRISPR library and the MetabolicAtlas (https://metabolicatlas.org), which includes not only strict metabolic enzymes but also regulators of metabolism (e.g. transcription factors known to be involved in the activation of metabolic pathways).

2. ROS are typically generated in the cytoplasm and the mitochondria and are only known to be transiently transferred to the nucleus. Therefore, I fail to see how an increase in ROS levels in the nucleus would imply that metabolic enzymes are associated with chromatin.

We apologize for jumping to conclusions. It is very exciting for us to see that enzymes of the ETC localize on chromatin during DNA damage, but we certainly do not have any proof of causality, and this could be a consequence of some sort of mitochondrial dysfunction caused by etoposide treatment that allows ETC enzymes leak from the mitochondria. We now performed immunofluorescence targeting COX4 which was among the top chromatin-relocalizing enzymes following etoposide release (Figure S2J). We observed a clear increase of COX4 in the nucleus of the cells following such treatment (Figure 3A). Similar experiments have been conducted using NCS and carboplatin. While we observe a clear increase of nuclear COX4 (and nuclear ROS) with carboplatin, the same does not occur with NCS treatment. Interestingly, PRDX1 translocates to the nucleus only in etoposide- and carboplatin-treated cells, but not in NCS-treated (Figure S5A-D).

These data demonstrate that COX4 increase in the cellular nucleus in parallel to nuclear ROS, similar to PRDX1. Even though answering why COX4 translocates to the nucleus is certainly intriguing, we

believe that this goes beyond the scope of our work, which aims at offering a comprehensive characterization of the DNA damage response from a metabolic point of view and investigating the role of PRDX1 in it.

Interestingly, it has been recently published that mitochondrial ROS may not be responsible for DNA damage induction (doi: https://doi.org/10.1101/2023.03.29.534749), suggesting that localised ROS generation may be what really causes the damage. This finding may be the starting point to connect COX4 (and other ETC enzymes) nuclear localization to nuclear ROS production and DNA damage. Although extremely interesting, this aspect is beyond the scope of this manuscript and would require an entire full study.

3. Etoposide forms covalent TOP2-DNA cleavage complexes affecting transcription, DNA replication and triggering some types of DNA damage. It may be an over-interpretation to define the response of cells to etoposide as "the DNA damage response".

We thank the reviewer for this observation. Even though etoposide has been classically used as a double-strand break inducer, we agree that it provokes effects that are beyond DNA damage. As already mentioned, we have now used carboplatin and NCS to validate key results.

4. ENO1 is known to bind to chromatin and to act as a transcription repressor. The role of PRDX1 on DDR is also well established for telomeric DNA damage and in prdx1 KO mice. The authors may wish to re-phrase or else highlight the novelty of their findings in view of what has already been published.

We thank the reviewer for the suggestion. While we do not follow up on ENO1, we clearly show that we do not observe colocalization of DNA damage and telomere markers in the 2 different PRDX1 KO cell lines used (Figure 4C-D and Supplementary Figure 7I), indicating an independent role of PRDX1 in DNA damage beyond telomeres. Additionally, the role of PRDX1 in the breakdown of intracellular aspartate levels between GSH synthesis and *de novo* nucleotide metabolism, which we uncover in this study, is entirely novel. This equilibrium gets compromised during the DNA damage response due to the increased ROS levels, thus making PRDX1 an important protein in DNA repair. Similarly, the nuclear relocalization of PRDX1 following nuclear ROS increase has not been previously shown before.

5. Lower aspartate-dependent metabolites in PRDX1 could be a indirect contributing factor to genome instability but the specificity of aspartate synthesis to PRDX1 per se upon DNA damage is not clearly shown.

We thank the reviewer for this observation. As explained above, by digging into our metabolomics experiment we observed that in the absence of PRDX1, the aspartate required for nucleotide synthesis may be directed towards GSH synthesis due to GSH over-oxidation into GSSG, impairing *de novo* nucleotide synthesis (Figure 5E-M and S6E-F).

Moreover, essentiality data (https://depmap.org/portal/achilles/) extracted from PRDX1 high and low-expressing cells (CCLE) corroborated our metabolomics experiment. In low-expressing PRDX1 cells, genes related to nucleotide metabolism and GSH biosynthesis are much more essential, together with genes of DNA repair, especially homologous recombination and cell cycle checkpoints (Figure S6G).

These new data link PRDX1 to aspartate availability towards *de novo* nucleotide synthesis. To further confirm this evidence, we have now treated PRDX1-depleted cells with aspartate, ascorbic acid, and their combination and show that all treatments reduce the basal DNA damage levels of these cells (Figure 6J). Additionally, we challenged PRDX1-depleted cells with etoposide and observed that when cultured in presence of nucleotides they are much faster reducing γH2AX foci (Figure 6K). The metabolite reconstitution experiments corroborate the hypothesis arising from our metabolomics results.

Reviewer #3:

In this manuscript, using elegant CRISPR screens focusing on metabolic enzymes, a joint effort between the Loizou and Sdelci labs reveals previously unidentified role for the oxidoreductase peroxiredoxin 1 (PRDX1) in scavenging nuclear ROS following DNA damage and aspartate synthesis.

We thank the reviewer for this positive comment.

The authors highlight some interesting features on several metabolic proteins and metabolites suggesting conceptually novel and interesting links to the DNA damage response worth investigating, but there is only little experimental evidence presented beyond the screens, with the notable exception of PRDX1. Thus, screen and metabolomics data are comprehensive and will provide a suitable resource for the field. Yet, the manuscript falls in my opinion short in firmly establishing a prominent role for PRDX1 in response to (exogenous) DNA damage.

We hope that the new results will convince the reviewer about the fact that the presence of PRDX1 is essential for DNA damage repair.

• Different from what the microscopic images are indicating (Figure 1C), there is quite a high level of mitochondrial and nuclear (physiological) ROS in untreated cells, that is, on average, almost indistinguishable from those of Etoposide treated cells (Figure 1D), even though significant p-values seemingly claim otherwise. Can the authors comment on that? It's true that in 1D it looks like ROS levels are very close even if significantly lower for DMSO, while on the pictures in 1C it does look really lower, especially for mito ROS.

We thank the reviewer for this interesting comment. Statistical significance in experiments with numerous observations is achieved even for small differences if those are constant, which should be interpreted as a clear tendency for the two populations to differ from each other. In the commented experiment (as well as in the majority) we analyzed more than 500 cells in each condition, giving statistical power to detect small deviations from the control condition. The images shown are meant to facilitate the observations we make about increased ROS 24hr after Etoposide, and they are accompanied by the quantification to show that the changes we observe are not dramatic, but significant.

It would be interesting to see whether co-treatment of cells with antioxidants (e.g., ascorbic acid) would lead to a restoration of mitochondrial ROS after etoposide.

We thank the reviewer for this observation. As explained before, by digging into our metabolomics experiment we observed that in the absence of PRDX1, the aspartate required for nucleotide

synthesis may be directed towards GSH synthesis due to GSH over-oxidation into GSSG, impairing *de novo* nucleotide synthesis (Figure 5E-M and S6E-F).

Moreover, essentiality data (https://depmap.org/portal/achilles/) extracted from PRDX1 high and low-expressing cells (CCLE) corroborated our metabolomics experiment. In low-expressing PRDX1 cells, genes related to nucleotide metabolism and GSH biosynthesis are much more essential, together with genes of DNA repair, especially homologous recombination and cell cycle checkpoints (Figure S6G).

These new data link PRDX1 to aspartate availability towards *de novo* nucleotide synthesis. To further confirm this evidence, we have now treated PRDX1-depleted cells with aspartate, ascorbic acid, and their combination and show that all treatments reduce the basal DNA damage levels of these cells (Figure 6J). Additionally, we challenged PRDX1-depleted cells with etoposide and observed that when cultured in presence of nucleotides they are much faster in reducing γH2AX foci (Figure 6K). The metabolite reconstitution experiments corroborate the hypothesis arising from our metabolomics results.

• To corroborate the phenotypes reported for PRDX1 depletion, I would strongly recommend the authors monitoring both etoposide-induced nuclear ROS and γH2AX levels and metabolites in cells inducibly-expressing PRDX1.

We thank the reviewer for this suggestion. We tried to overexpress PRDX1 with small success given the fact that the level of overexpression was minimal. However, we conducted experiments using different DNA damage-inducing agents that corroborated our hypothesis of PRDX1 nuclear recruitment upon nuclear ROS accumulation. While we observed a clear increase of nuclear ROS also with carboplatin, the same does not occur with NCS treatment. Interestingly, PRDX1 translocates to the nucleus only in etoposide- and carboplatin-treated cells, but not in NCS-treated (Figure S5A-D). These data demonstrate that PRDX1 translocates to the nucleus when there is an increase in nuclear ROS. In agreement with this, we demonstrate that PRDX1 accumulates in the nucleus during the G2 phase of the cell cycle (Figure 4G-H), which corresponds to the phase where ROS increase the most (10.1016/j.cels.2019.01.005, 10.1016/j.devcel.2022.06.008).

• I cannot really follow the authors reasoning of focusing on PRDX1 for their follow-up studies, as its increase on chromatin abundance following etoposide treatment is not statistically significant. Better explained. Along the same lines, PRDX1 depletion (using shRNA) in untreated cells already results in an increase in ROS (and gH2X foci), comparable to that observed upon etoposide (Figures 3A and 3B). Consistent with this, and as shown in Figure 3C, PRDX1 does not form foci in response to DSB induction and accumulate in the nucleus at a timepoint where γH2AX have disappeared (Figure 3C). I do not understand how the authors see a clear correlation between PRDX1 and DNA damage levels, as stated on p. 8. In fact, there is a much stronger contribution of PRDX1 in scavenging ROS and influencing the metabolic state of cells in absence of exogenous DNA damage.

We thank the reviewer for this criticism that made us brainstorm about our data quite a bit. We believe that the fact that PRDX1 accumulates in the nucleus when γH2AX has disappeared does not mean that PRDX1 does not play a role in the DNA damage response. Our experiments, both the chromatin proteomics and the metabolomics, show that despite the lack of γH2AX 24 hours after etoposide release, the DNA damage response is not yet over and the cells did not recover their basal state. In agreement with this, nuclear ROS levels reach a maximum at 24 hours after etoposide release, when PRDX1 is also recruited in the nucleus even though γH2AX is already gone.

It is well reported that PRDX1 has a role in scavenging ROS, but the fact that it localizes to the nucleus during the DNA damage response to scavenge etoposide-induced ROS indicates that it has a clear purpose in the DNA damage response. It is, therefore, expected that the lack of PRDX1 would lead, as we observe, to DNA damage accumulation in replicating cells, which constantly have to deal with some *physiological* DNA damage that might be not cleared properly in absence of PRDX1.

Experiments that could help solving this issues: PRDX1-depleted cells hypersensitive to etoposide treatment (probably not given that PRDX1 is not SL with etoposide), or, vice versa, are PRDX1-overexpressing cells more resistant to DNA damage treatment?

The overexpression of PRDX1 did not result in an evident increase in protein levels. However, we now show that PRDX1-depleted cells are significantly more sensitive to DNA-inducing agents that provoke nuclear ROS increase (etoposide and carboplatin, Figure 4B).

• Establishing a pivotal role of PRDX1 in alleviating ROS-induced replication stress, I recommend the authors to examine replication fork phenotypes (e.g. stalling, reversal/restart, nascent strand) degradation) in unstressed cells and in response to DNA damage using classical DNA fiber spreading protocols.

Even though this experiment goes beyond the scope of our study, we performed a DNA fiber spreading assay and observed that PRDX1-depleted cells in presence of etoposide show a much-reduced fork progression velocity (Figure 6G), corroborating our hypothesis.

Minor comments:

• Please describe in the materials section the CRISRP-Cas9 library (how many genes, how many sgRNAs/per gene).

We thank the reviewer for this observation, and we have now included this information.

• p.3: H2AX phosphorylation can no longer be considered as a (specific) DSB marker but rather as a general marker of DNA damage.

We thank the reviewer for this observation, and we have now changed it in the text.

• In Figures 2B and 2C, please indicate in the volcano plots the threshold for 'significant hits.

Statistically significant protein hits are those with p.adj values less than 0.05. We now specified it in the figure legend for clarity.

• A proteomics approach is taken to investigate which metabolic enzymes are becoming enriched at damage chromatin. The analysis apparently revealed 11 metabolic enzymes that were significantly altered (enriched or depleted on chromatin, immediately after (no release) or 24h-post etoposide (Table S1). However, Table S1 does not list which of those 11 proteins belongs to which of the four different 'categories. Moreover, Rad18 and Bard1 are also included in that list (how comes?)

We have now clarified it in the text. Briefly, metabolic factors are retrieved from the CRISPR library and the MetabolicAtlas (https://metabolicatlas.org), which includes not only strict metabolic

enzymes but also metabolism regulators (e.g. transcription factors known to be involved in the activation of metabolic pathways).

I think there is in general quite some confusions in the text and/or in the Legend on what is displayed on Table S1 ("Gene lists belonging in each of the categories of S2F" or "11 hits"?)

We apologize for the confusion. For further clarity, the specific sheet of the Table S1 that shows the 11 metabolic enzymes significantly altered on chromatin is now referenced, and additional information regarding these proteins has been added in the table: “Among the significantly altered proteins, 11 were metabolic enzymes (Table S1 'chromatindiff_Metabolic' sheet).

C/ Dr. Aiguader 88, 08003 Barcelona +34 316 01 00 sara.sdelci@crg.eu www.crg.es
Lazarettgasse 14, AKH BT 25.3, 1090 Vienna, Austria, Phone +43-1/40160-70058, joanna_loizou@hotmail.com www.cemm.at

12th Apr 2023

Manuscript Number: MSB-2022-11267RR
Title: A metabolic map of the DNA damage response identifies PRDX1 in nuclear ROS scavenging and aspartate availability.

Dear Sara,

Thank you again for submitting your work to Molecular Systems Biology. We have now heard back from reviewer #3 who agreed to evaluate your study. Unfortunately, reviewer #1 was not available this time. As you will see below, the reviewer #3 is satisfied with the performed revisions and supports publication. They list however a couple of remaining concerns and recommend toning down a few statements. We would ask you to address these remaining concerns in a revision.

We would also ask you to address some editorial issues listed below.

- Our data editors have noticed some unclear or missing information in the figure legends, please see the attached .doc file. Please make all requested text changes using the attached file and *keeping the "track changes" mode* so that we can easily access the edits made.

- The keywords should be reduced to 5.

- Please include a "Disclosure & Competing Interests Statement" in the main text.

- All fields in the Author Checklist should be completed.

- There is a callout to Table S6, but such a Table does not exist. Please correct.

- Tables S1-S5 and Supplementary_Data1-3 should be changed to Dataset EV1-EV8. Pleas provide one file per dataset. Each xls file should contain a description of the EV Dataset in a separate tab.

- We have replaced Supplementary Information by the Expanded View (EV format). In this case all additional figures can be displayed as EV Figures. EV Figures should be provided as individual files and their legends should be included in the main text. For detailed instructions regarding Expanded View please refer to our Author Guidelines:
.

- Tables S1-S12 should be provided as Datasets EV1-EV12. Please provide one file per Dataset. Each file should contain a brief description of the Dataset in a separate tab of the .xls file.

- Please include 5 keywords.

- Please provide a "standfirst text" summarizing the study in one or two sentences (approximately 250 characters), three to four "bullet points" highlighting the main findings and a "synopsis image" (550px width and max 400px height, jpeg format) to highlight the paper on our homepage.

- All Materials and Methods need to be described in the main text. We would encourage you to use 'Structured Methods', our new Materials and Methods format. According to this format, the Material and Methods section should include a Reagents and Tools Table (listing key reagents, experimental models, software and relevant equipment and including their sources and relevant identifiers) followed by a Methods and Protocols section in which we encourage the authors to describe their methods using a step-by-step protocol format with bullet points, to facilitate the adoption of the methodologies across labs. More information on how to adhere to this format as well as downloadable templates (.doc or .xls) for the Reagents and Tools Table can be found in our author guidelines: . An example of a Method paper with Structured Methods can be found here:

- Currently the "reagents and tools table" only lists the antibodies. If you would like to use the Structured Methods format we would ask you to list all reagents in this table (see above).

- For data quantification: please specify the name of the statistical test used to generate error bars and P values, the number (n) of independent experiments (specify technical or biological replicates) underlying each data point and the test used to calculate p-values in each figure legend. The figure legends should contain a basic description of n, P and the test applied. Graphs must include a description of the bars and the error bars (s.d., s.e.m.).

Please resubmit your revised manuscript online, with a covering letter listing amendments and responses to each point raised by the referees. Please resubmit the paper **within one month** and ideally as soon as possible. If we do not receive the revised

manuscript within this time period, the file might be closed and any subsequent resubmission would be treated as a new manuscript. Please use the Manuscript Number (above) in all correspondence.

Click on the link below to submit your revised paper.

https://msb.msubmit.net/cgi-bin/main.plex

As a matter of course, please make sure that you have correctly followed the instructions for authors as given on the submission website.

Thank you for submitting this paper to Molecular Systems Biology.

Kind regards,

Maria

Maria Polychronidou, PhD
Senior Editor
Molecular Systems Biology

If you do choose to resubmit, please click on the link below to submit the revision online before 12th May 2023.

https://msb.msubmit.net/cgi-bin/main.plex

IMPORTANT:
Please note that corresponding authors are required to supply an ORCID ID for their name upon submission of a revised manuscript (EMBO Press signed a joint statement to encourage ORCID adoption).
(https://www.embopress.org/page/journal/17444292/authorguide#editorialprocess)
Currently, our records indicate that the ORCID for your account is 0000-0003-1330-4364.

Please click the link below to modify this ORCID:
Link Not Available

The system will prompt you to fill in your funding and payment information. This will allow Wiley to send you a quote for the article processing charge (APC) in case of acceptance. This quote takes into account any reduction or fee waivers that you may be eligible for. Authors do not need to pay any fees before their manuscript is accepted and transferred to the publisher.

As a matter of course, please make sure that you have correctly followed the instructions for authors as given on the submission website.

*** PLEASE NOTE *** As part of the EMBO Press transparent editorial process initiative (see our Editorial at https://dx.doi.org/10.1038/msb.2010.72 , Molecular Systems Biology will publish online a Review Process File to accompany accepted manuscripts. When preparing your letter of response, please be aware that in the event of acceptance, your cover letter/point-by-point document will be included as part of this File, which will be available to the scientific community. More information about this initiative is available in our Instructions to Authors. If you have any questions about this initiative, please contact the editorial office (msb@embo.org).

Reviewer #3:

The authors have satisfactorily addressed all my major concerns and provide new experimental data substantiating a role of PRDX1 in the DNA damage response.
Overall, I think that their combined findings are consistent with a role for PRDX1 in the control of nuclear ROS scavenging and aspartate availability (as indicated in the title). However, I am still not convinced that PPRDX1 has a "central role in DNA damage repair" or a "crucial role as a DNA damage surveillance factor" (as indicated in the abstract). For such a strong claim to made, the cellular sensitivity observed upon treatment with etoposide and cisplatin is too marginal (new Figure 4G), and I would recommend revising the abstract accordingly.
It would have been very interesting to analyse DDR phenotypes in PRRDX1-overexpressing cells (e.g. are they resistant to etoposide?). Do the authors have an explanation why it was not possible for the authors to create such a system?

08.05.2023

A metabolic map of the DNA damage response identifies PRDX1 in the control of nuclear ROS scavenging and aspartate availability

Response to the Reviewer #3:

The authors have satisfactorily addressed all my major concerns and provide new experimental data substantiating a role of PRDX1 in the DNA damage response.

Overall, I think that their combined findings are consistent with a role for PRDX1 in the control of nuclear ROS scavenging and aspartate availability (as indicated in the title). However, I am still not convinced that PPRDX1 has a "central role in DNA

damage repair" or a "crucial role as a DNA damage surveillance factor" (as indicated in the abstract). For such a strong claim to made, the cellular sensitivity observed upon treatment with etoposide and cisplatin is too marginal (new Figure 4G), and I would recommend revising the abstract accordingly.

It would have been very interesting to analyse DDR phenotypes in PRRDX1-overexpressing cells (e.g. are they resistant to etoposide?). Do the authors have an explanation why it was not possible for the authors to create such a system?

We thank the reviewer for their positive assessment. We are delighted that our data satisfied their initial concerns.

As suggested, we toned down the abstract when we talk about PRDX1.

Our initial plan was to test whether overexpression of PRDX1 makes cells resistant to treatment with etoposide or carboplatin. We transduced U2-OS cells with a virus encoding PRDX1-Maroon. In our hands, the level of overexpression was generally very low (< 20% increase over endogenous protein) and the signal in the nucleus was not resembling the one of endogenous PRDX1. We speculate that the tagging may be affecting the stability and nuclear translocation of the protein and therefore the results obtained with this system may not be reliable. We anyway tested carboplatin treatment in these cells and observed slightly lower sensitivity (Figure 1). However, the low levels of PRDX1-Maroon overexpression and its nuclear exclusion make it difficult to interpret these results conclusively. As the reviewer said, the new data provided strengthen the connection between PRDX1 and DNA damage repair, so we have decided not to include this experiment in the current manuscript. We are now planning to use a smaller tag, such as FLAG or HA, which would hopefully interfere less with the protein behavior. Future lines of research in the lab will include investigating the relationship between ETC enzymes' nuclear translocation, nuclear ROS, and the nuclear requirement of PRDX1, and, therefore, this tool will be needed.

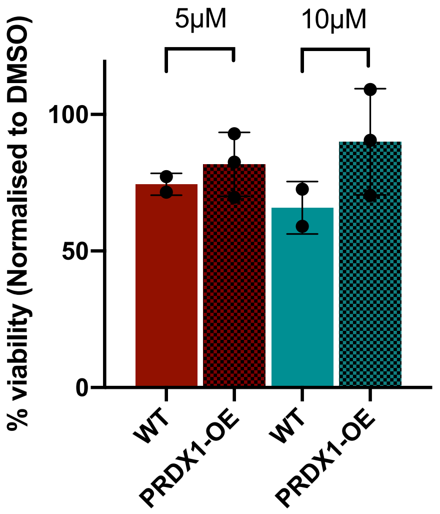

Figure 1. Carboplatin treatment (5 and 10 μM) of PRDX1 wild type cells and PRDX1-Maroon overexpressing cells for 72 hours. Cell count has been used to asses cell viability. Values have been normalized with the average of the DMSO treatments.

10th May 2023

Manuscript number: MSB-2022-11267RRR
Title: A metabolic map of the DNA damage response identifies PRDX1 in nuclear ROS scavenging and aspartate availability.

Dear Sara,

Thank you again for sending us your revised manuscript. We are now satisfied with the modifications made and I am pleased to inform you that your paper has been accepted for publication.

*** PLEASE NOTE *** As part of the EMBO Publications transparent editorial process initiative (see our Editorial at https://dx.doi.org/10.103/msb.2010.72), Molecular Systems Biology publishes online a Review Process File with each accepted manuscripts. This file will be published in conjunction with your paper and will include the anonymous referee reports, your point- by-point response and all pertinent correspondence relating to the manuscript. If you do NOT want this File to be published, please inform the editorial office at msb@embo.org within 14 days upon receipt of the present letter.

LICENSE AND PAYMENT:
All articles published in Molecular Systems Biology are fully open access: immediately and freely available to read, download and share.

Molecular Systems Biology charges an article processing charge (APC) to cover the publication costs. You, as the corresponding author for this manuscript, should have already received a quote with the article processing fee separately. Please let us know in case this quote has not been received.

Once your article is at Wiley for editorial production, you will receive an email from Wiley's Author Services system, which will ask you to log in and will present you with the publication license form for completion. Within the same system the publication fee can be paid by credit card, an invoice or pro forma can be requested.

Payment of the publication charge and the signed Open Access Agreement form must be received before the article can be published online.

Upon acceptance it is mandatory for you to return the completed payment form. Failure to send back the form may result in a delay in the publication of your paper.

Molecular Systems Biology articles are published under the Creative Commons licence CC BY, which facilitates the sharing of scientific information by reducing legal barriers, while mandating attribution of the source in accordance to standard scholarly practice.

Proofs will be forwarded to you within the next 2-3 weeks.

Thank you very much for submitting your work to Molecular Systems Biology.

Best wishes,

Maria

Maria Polychronidou, PhD
Senior Editor
Molecular Systems Biology

EMBO Press Author Checklist

Corresponding Author Name: Sara Sdelci	
Journal Submitted to: Molecular System Biology	
Manuscript Number: MSB-2022-11267RR	

USEFUL LINKS FOR COMPLETING THIS FORM
The EMBO Journal - Author Guidelines
EMBO Reports - Author Guidelines
Molecular Systems Biology - Author Guidelines
EMBO Molecular Medicine - Author Guidelines

Reporting Checklist for Life Science Articles (updated January
This checklist is adapted from Materials Design Analysis Reporting (MDAR) Checklist for Authors. MDAR establishes a minimum set of requirements in transparent reporting in the life sciences (see Statement of Task: 10.31222/osf.io/9sm4x). Please follow the journal's guidelines in preparing your
Please note that a copy of this checklist will be published alongside your article.

Abridged guidelines for figures
1. Data
The data shown in figures should satisfy the following conditions:
- ➞ the data were obtained and processed according to the field's best practice and are presented to reflect the results of the experiments in an accurate and unbiased manner.
- ➞ ideally, figure panels should include only measurements that are directly comparable to each other and obtained with the same assay.
- ➞ plots include clearly labeled error bars for independent experiments and sample sizes. Unless justified, error bars should not be shown for technical
- ➞ if n<5, the individual data points from each experiment should be plotted. Any statistical test employed should be justified.
- ➞ Source Data should be included to report the data underlying figures according to the guidelines set out in the authorship guidelines on Data

2. Captions
Each figure caption should contain the following information, for each panel where they are relevant:
- ➞ a specification of the experimental system investigated (eg cell line, species name).
- ➞ the assay(s) and method(s) used to carry out the reported observations and measurements.
- ➞ an explicit mention of the biological and chemical entity(ies) that are being measured.
- ➞ an explicit mention of the biological and chemical entity(ies) that are altered/varied/perturbed in a controlled manner.
- ➞ the exact sample size (n) for each experimental group/condition, given as a number, not a range;
- ➞ a description of the sample collection allowing the reader to understand whether the samples represent technical or biological replicates (including how many animals, litters, cultures, etc.).
- ➞ a statement of how many times the experiment shown was independently replicated in the laboratory.
- ➞ definitions of statistical methods and measures:
  - common tests, such as t-test (please specify whether paired vs. unpaired), simple χ2 tests, Wilcoxon and Mann-Whitney tests, can be unambiguously identified by name only, but more complex techniques should be described in the methods section;
  - are tests one-sided or two-sided?
  - are there adjustments for multiple comparisons?
  - exact statistical test results, e.g., P values = x but not P values < x;
  - definition of 'center values' as median or average;
  - definition of error bars as s.d. or s.e.m.

Please complete ALL of the questions below.
Select "Not Applicable" only when the requested information is not relevant for your study.

Materials

Newly Created Materials	Information included in the manuscript?	In which section is the information available? (Reagents and Tools Table, Materials and Methods, Figures, Data Availability Section)
New materials and reagents need to be available; do any restrictions apply?	Not Applicable	(no restriction applies)

Antibodies	Information included in the manuscript?	In which section is the information available? (Reagents and Tools Table, Materials and Methods, Figures, Data Availability Section)
For **antibodies** provide the following information:<br>- Commercial antibodies: RRID (if possible) or supplier name, catalogue number and or/clone number<br>- Non-commercial: RRID or citation	Yes	Reagents and Tools Table

DNA and RNA sequences	Information included in the manuscript?	In which section is the information available? (Reagents and Tools Table, Materials and Methods, Figures, Data Availability Section)
**Short novel DNA or RNA including primers, probes**: provide the sequences.	Yes	Materials and Methods

Cell materials	Information included in the manuscript?	In which section is the information available? (Reagents and Tools Table, Materials and Methods, Figures, Data Availability Section)
**Cell lines:** Provide species information, strain. Provide accession number in repository **OR** supplier name, catalog number, clone number, and/**OR** RRID.	Yes	Materials and Methods
**Primary cultures:** Provide species, strain, sex of origin, genetic modification status.	Not Applicable	
Report if the cell lines were recently **authenticated** (e.g., by STR profiling) and tested for mycoplasma contamination.	Yes	Materials and Methods

Experimental animals	Information included in the manuscript?	In which section is the information available? (Reagents and Tools Table, Materials and Methods, Figures, Data Availability Section)
**Laboratory animals or Model organisms:** Provide species, strain, sex, age, genetic modification status. Provide accession number in repository **OR** supplier name, catalog number, clone number, **OR** RRID.	Not Applicable	
**Animal observed in or captured from the field:** Provide species, sex, and age where possible.	Not Applicable	
Please detail **housing and husbandry conditions**.	Not Applicable	

Plants and microbes	Information included in the manuscript?	In which section is the information available? (Reagents and Tools Table, Materials and Methods, Figures, Data Availability Section)
**Plants:** provide species and strain, ecotype and cultivar where relevant, unique accession number if available, and source (including location for collected wild specimens).	Not Applicable	
**Microbes:** provide species and strain, unique accession number if available, and source.	Not Applicable	

Human research participants	Information included in the manuscript?	In which section is the information available? (Reagents and Tools Table, Materials and Methods, Figures, Data Availability Section)
If collected and within the bounds of privacy constraints report on age, sex and gender or ethnicity for all study participants.	Not Applicable	

Core facilities	Information included in the manuscript?	In which section is the information available? (Reagents and Tools Table, Materials and Methods, Figures, Data Availability Section)
If your work benefited from core facilities, was their service mentioned in the acknowledgments section?	Yes	Acknowledgements section

Design

Study protocol	Information included in the manuscript?	In which section is the information available? (Reagents and Tools Table, Materials and Methods, Figures, Data Availability Section)
If study protocol has been **pre-registered, provide DOI in the manuscript**. For clinical trials, provide the trial registration number **OR** cite DOI.	Not Applicable	
Report the **clinical trial registration number** (at ClinicalTrials.gov or equivalent), where applicable.	Not Applicable	

Laboratory protocol	Information included in the manuscript?	In which section is the information available? (Reagents and Tools Table, Materials and Methods, Figures, Data Availability Section)
Provide DOI OR other citation details if **external detailed step-by-step protocols** are available.	Not Applicable	

Experimental study design and statistics	Information included in the manuscript?	In which section is the information available? (Reagents and Tools Table, Materials and Methods, Figures, Data Availability Section)
Include a statement about **sample size** estimate even if no statistical methods were used.	Yes	figure legends and methods
Were any steps taken to minimize the effects of subjective bias when allocating animals/samples to treatment (e.g. **randomization procedure**)? If yes, have they been described?	Not Applicable	
Include a statement about **blinding** even if no blinding was done.	Yes	Materials and Methods
Describe **inclusion/exclusion criteria** if samples or animals were excluded from the analysis. Were the criteria pre-established?<br><br>If sample or data points were omitted from analysis, report if this was due to attrition or intentional exclusion and provide justification.	Not Applicable	
For every figure, are **statistical tests** justified as appropriate? Do the data meet the assumptions of the tests (e.g., normal distribution)? Describe any methods used to assess it. Is there an estimate of variation within each group of data? Is the variance similar between the groups that are being statistically compared?	Yes	figure legends

Sample definition and in-laboratory replication	Information included in the manuscript?	In which section is the information available? (Reagents and Tools Table, Materials and Methods, Figures, Data Availability Section)
In the figure legends: state number of times the experiment was **replicated** in laboratory.	Yes	figure legends
In the figure legends: define whether data describe **technical or biological replicates**.	Yes	figure legends

Ethics

Ethics	Information included in the manuscript?	In which section is the information available? (Reagents and Tools Table, Materials and Methods, Figures, Data Availability Section)
Studies involving **human participants**: State details of **authority granting ethics approval** (IRB or equivalent committee(s), provide reference number for approval.	Not Applicable	
Studies involving **human participants**: Include a statement confirming that **informed consent** was obtained from all subjects and that the experiments conformed to the principles set out in the WMA Declaration of Helsinki and the Department of Health and Human Services Belmont Report.	Not Applicable	
Studies involving **human participants:** For publication of **patient photos**, include a statement confirming that consent to publish was obtained.	Not Applicable	
Studies involving experimental **animals**: State details of **authority granting ethics approval** (IRB or equivalent committee(s), provide reference number for approval. Include a statement of compliance with ethical regulations.	Not Applicable	
Studies involving **specimen and field samples:** State if relevant **permits** obtained, provide details of authority approving study; if none were required, explain why.	Not Applicable	

Dual Use Research of Concern (DURC)	Information included in the manuscript?	In which section is the information available? (Reagents and Tools Table, Materials and Methods, Figures, Data Availability Section)
Could your study fall under dual use research restrictions? Please check biosecurity documents and list of **select agents and toxins** (CDC): https://www.selectagents.gov/sat/list.htm	Not Applicable	
If you used a select agent, is the security level of the lab appropriate and reported in the manuscript?	Not Applicable	
If a study is subject to dual use research of concern regulations, is the name of the **authority granting approval and reference number** for the regulatory approval provided in the manuscript?	Not Applicable	

Reporting

The MDAR framework recommends adoption of discipline-specific guidelines, established and endorsed through community initiatives. Journals have their own policy about requiring specific guidelines and recommendations to complement MDAR.

Adherence to community standards	Information included in the manuscript?	In which section is the information available? (Reagents and Tools Table, Materials and Methods, Figures, Data Availability Section)
State if relevant guidelines or checklists (e.g., **ICMJE, MIBBI, ARRIVE, PRISMA**) have been followed or provided.	Not Applicable	
For **tumor marker prognostic studies,** we recommend that you follow the **REMARK** reporting guidelines (see link list at top right). See author guidelines, under 'Reporting Guidelines'. Please confirm you have followed these guidelines.	Not Applicable	
For **phase II and III randomized controlled trials**, please refer to the **CONSORT** flow diagram (see link list at top right) and submit the CONSORT checklist (see link list at top right) with your submission. See author guidelines, under 'Reporting Guidelines'. Please confirm you have submitted this list.	Not Applicable	

Data Availability

Data availability	Information included in the manuscript?	In which section is the information available? (Reagents and Tools Table, Materials and Methods, Figures, Data Availability Section)
Have **primary datasets** been deposited according to the journal's guidelines (see 'Data Deposition' section) and the respective accession numbers provided in the Data Availability Section?	Yes	data availability section
Were **human clinical and genomic datasets** deposited in a public access-controlled repository in accordance to ethical obligations to the patients and to the applicable consent agreement?	Not Applicable	
Are **computational models** that are central and integral to a study available without restrictions in a machine-readable form? Were the relevant accession numbers or links provided?	Not Applicable	
If publicly available data were reused, provide the respective **data citations in the reference list**.	Yes	figure legend, manuscript text and reference list
